# Transcriptome Profiling Combined With Activities of Antioxidant and Soil Enzymes Reveals an Ability of *Pseudomonas* sp. CFA to Mitigate *p*-Hydroxybenzoic and Ferulic Acid Stresses in Cucumber

**DOI:** 10.3389/fmicb.2020.522986

**Published:** 2020-10-27

**Authors:** Yue Zhang, Chang-Xia Chen, Hui-Ping Feng, Xiu-Juan Wang, Ute Roessner, Robert Walker, Zeng-Yan Cheng, Yan-Qiu An, Binghai Du, Ji-Gang Bai

**Affiliations:** ^1^State Key Laboratory of Crop Biology, College of Life Sciences, Shandong Agricultural University, Tai’an, China; ^2^Shandong Engineering Research Center of Plant-Microbial Restoration for Saline-Alkali Land, College of Life Sciences, Shandong Agricultural University, Tai’an, China; ^3^School of BioSciences, Faculty of Science, The University of Melbourne, Parkville, VIC, Australia

**Keywords:** cucumber, ferulic acid, *p*-hydroxybenzoic acid, *Pseudomonas*, rate-limiting enzymes, small non-coding RNA

## Abstract

Continuous-cropping leads to obstacles in crop productivity by the accumulation of *p*-hydroxybenzoic acid (PHBA) and ferulic acid (FA). In this study, a strain CFA of *Pseudomonas* was shown to have a higher PHBA- and FA-degrading ability in media and soil and the mechanisms underlying this were explored. Optimal conditions for PHBA and FA degradation by CFA were 0.2 g/l of PHBA and FA, 37°C, and pH 6.56. Using transcriptome analysis, complete pathways that converted PHBA and FA to acetyl coenzyme A were proposed in CFA. When CFA was provided with PHBA and FA, we observed upregulation of genes in the pathways and detected intermediate metabolites including vanillin, vanillic acid, and protocatechuic acid. Moreover, 4-hydroxybenzoate 3-monooxygenase and vanillate *O*-demethylase were rate-limiting enzymes by gene overexpression. Knockouts of small non-coding RNA (*sRNA*) genes, including *sRNA 11*, *sRNA 14*, *sRNA 20*, and *sRNA 60*, improved the degradation of PHBA and FA. When applied to cucumber-planted soil supplemented with PHBA and FA, CFA decreased PHBA and FA in soil. Furthermore, a reduction of superoxide radical, hydrogen peroxide, and malondialdehyde in cucumber was observed by activating superoxide dismutase, catalase, glutathione peroxidase, ascorbate peroxidase, glutathione reductase, dehydroascorbate reductase, and monodehydroascorbate reductase in seedlings, increasing the reduced glutathione and ascorbate in leaves, and inducing catalase, urease, and phosphatase in the rhizosphere. CFA has potential to mitigate PHBA and FA stresses in cucumber and alleviate continuous-cropping obstacles.

## Introduction

Continuous cropping is a common practice in the intensive farming of many countries (Li et al., 2016). However, when monocrops, including cucumber, are cultivated in the same land annually without interruption, continuous-cropping obstacles can occur. These obstacles can lead to suppression of growth, development, and production of the crops (Qin et al., 2017). Meantime, phenolic compounds are widely distributed in plants, and they will accumulate in soil through root deposition due to continuous cropping (Rice, 1979). Low concentrations of phenolic compounds can be applied to mitigate heat (Zhang et al., 2012) and dehydration (Li et al., 2013) stresses in cucumber. High concentrations of these compounds have herbicidal activities and inhibit the germination and initial growth of weeds (Reigosa et al., 1999; Stupnicka-Rodzynkiewicz et al., 2006; Uddin et al., 2012). However, the accumulation of phenolic compounds in soil have allelopathic potential (Reigosa et al., 1999) and will also cause secondary oxidative stresses in many plants (Imatomi et al., 2013), being one important reason for continuous-cropping obstacles (Li et al., 2016). Therefore, degrading phenolic compounds in soil may contribute to mitigating continuous-cropping obstacles. *Pseudomonas aeruginosa* can efficiently degrade benzoic acid at pH 7 (Razika et al., 2010). According to Xu et al. (2008), in soil of cucumber continuously cropped for 7 years, *p*-hydroxybenzoic acid (PHBA, 4-hydroxybenzoic acid) and ferulic acid (FA) are the main phenolic compounds, while root diseases and continuous-cropping obstacles occur seriously; after *Phanerochaete chrysosporium* is applied to degrade these compounds, the occurrence of root diseases and continuous-cropping obstacles is reduced. Based on these results, a study by Wu et al. (2019) isolated a PHBA- and FA-degrading strain of *Streptomyces* and applied it into cucumber-planted soil, where PHBA and FA were added. They found that the strain mitigates PHBA and FA stresses in cucumber and has a potential to alleviate the continuous-cropping obstacle of this plant. Chen et al. (2015) reported that a strain CSY-P1 of *Pseudomonas* has a PHBA-degrading ability in soil and thereby mitigates PHBA stress in cucumber. However, *Pseudomonas* strains have not been applied to degrade the mixture of PHBA and FA in planted soil.

Using phenolic compound-degrading strains of *Pseudomonas*, both PHBA (Venturi et al., 1998) and FA (Narbad and Gasson, 1998) can be decomposed into protocatechuic acid (PA), and the enzymes involved in the metabolisms have been identified. Harwood and Parales (1996) reported the enzymes, which could convert PA to β-ketoadipate in *Pseudomonas putida*. We hypothesized that β-ketoadipate might be decomposed into acetyl coenzyme A (CoA) by *Pseudomonas*. To date, the complete pathways that convert PHBA and FA to acetyl CoA via PA and β-ketoadipate have not been proposed in *Pseudomonas*. On the other hand, rate-limiting steps and enzymes can determine the overall rate of a metabolic pathway (Lagace and Ridgway, 2005). Since PHBA and FA degradation by *Pseudomonas* might both have the β-ketoadipate pathway (Harwood and Parales, 1996; Narbad and Gasson, 1998; Venturi et al., 1998), the degradation rate of each phenolic compound in the mixture of PHBA and FA would depend on the metabolic steps where PHBA and FA are separately converted into PA. Although *O*-demethylation is a rate-limiting step, where vanillate *O*-demethylase catalyzes the *O*-demethylation of vanillic acid to PA (Buswell and Ribbons, 1988), all the rate-limiting steps and enzymes have not been identified when PHBA and FA are decomposed into PA by *Pseudomonas*. In addition, bacteria use small non-coding RNA (sRNA) that has complementarity with the target mRNA they regulate (Gottesman and Storz, 2011). Up to now, sRNA has not been reported to affect the PHBA and FA degradation in *Pseudomonas*.

**FIGURE 1:**
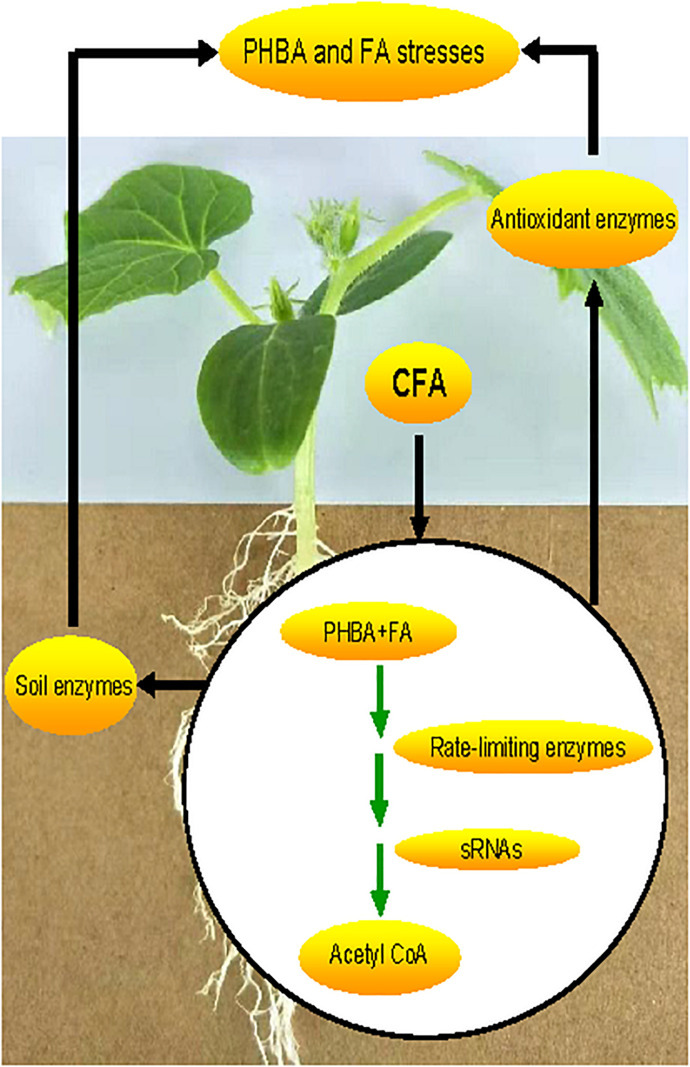
A hypothesis for CFA to mitigate *p*-hydroxybenzoic and ferulic acid stresses in cucumber.

In this study, a strain CFA was isolated from cucumber rhizospheric soil by separately using PHBA and FA as a sole carbon source. Then, the strain was identified to be *Pseudomonas* sp. CFA and applied to cucumber-planted soil, where the mixture of PHBA and FA was added. It was hypothesized that the strain CFA might have the PHBA- and FA-degrading abilities and would decompose the mixture of PHBA and FA in planted soil, thereby having a potential to mitigate this continuous-cropping obstacle of cucumber. To assess the mitigation effects of CFA on the stresses caused by the mixture of PHBA and FA, we determined the levels of superoxide radical (O_2_^⋅–^), hydrogen peroxide (H_2_O_2_), and malondialdehyde (MDA), which are markers for oxidative stress and accumulate in plants under conditions of phenolic compounds (Chen et al., 2015). To verify the level change of the three markers, we also measured the activities of antioxidant enzymes including superoxide dismutase (SOD), catalase (CAT), glutathione peroxidase (GSH-Px), ascorbate peroxidase (APX), monodehydroascorbate reductase (MDHAR), glutathione reductase (GR), and dehydroascorbate reductase (DHAR) and the contents of non-enzymic antioxidants such as reduced glutathione (GSH) and ascorbate (AsA), which can control the levels of O_2_^.–^ and H_2_O_2_ in plants and bacteria (Wu et al., 2019). Since soil enzymes including urease, phosphatase, and CAT are sensitive to phenolic compounds (Zhang et al., 2015) and can be indicators of the health and sustainability of one managed ecosystem (Kohler et al., 2009), their activities were assayed in this research when CFA was used to mitigate PHBA and FA stresses in cucumber. In order to elucidate one molecular mechanism underlying the PHBA and FA stress mitigation by CFA, the genome and transcriptome of this strain were sequenced in this study, and the genes involved in the complete pathways of PHBA and FA degradation were thereby isolated in *Pseudomonas*. Then, rate-limiting enzymes in the pathways were identified by using gene overexpression (Prelich, 2012), and *sRNA* gene knocked outs (Kim et al., 2015) to improve the PHBA and FA degradation. We hypothesized that, due to the PHBA and FA degradation that might be regulated by rate-limiting enzymes and *sRNA* genes, CFA would mitigate PHBA and FA stresses in cucumber by activating antioxidant and soil enzymes (Figure 1). The results obtained might provide a theoretical and practical basis for the application of CFA in alleviating the accumulation of PHBA and FA that leads to continuous-cropping obstacles.

## Materials and Methods

### Isolation and Identification of CFA

A strain CFA was isolated from cucumber rhizospheric soil according to Wu et al. (2019) by using M-9 medium supplemented with 0.5 g/l PHBA (Chen et al., 2015) and 0.5 g/l FA (Zhang et al., 2015), respectively. The PHBA- and FA-degrading abilities of CFA were promptly analyzed in medium by preliminarily determining the concentrations of PHBA and FA at 247 and 320 nm, respectively (Mantel and Stiller, 1976; Zhang et al., 2015). To preliminarily identify CFA, a fragment (GenBank accession number MH558575) of a 16S rRNA gene was amplified using the primers 1492R and 27F (Wang et al., 2011).

### Growth Conditions and Properties of CFA

To incubate CFA, 2 ml of strain cultures grown exponentially in Luria-Bertani (LB) liquid medium were inoculated into 100 ml of liquid M-9 medium supplemented with a mixture of PHBA and FA in the ratio of 42.82: 57.18 (Wu et al., 2018). Cell growth of CFA was measured at 600 nm (Mathew et al., 2007).

To promptly analyze the properties of CFA, the strain was incubated at 180 rpm for 6 h, and the percentages of degraded PHBA and FA in M-9 medium were preliminarily assessed at 247 and 320 nm, respectively. Based on the properties of CFA, Box-Behnken design was used to optimize the PHBA and FA degradation by CFA (Wu et al., 2018). The levels and codes of independent variables are given in Supplementary Table 1.

### High-Performance Liquid Chromatography (HPLC) Analysis of PHBA, FA and Their Degradation Products in Culture Solutions

When CFA cultures being centrifuged, the supernatants (10 ml) were freeze-dried at −84°C. Subsequently, the resulting residue was resuspended in 1 ml of methanol and subjected to HPLC analysis. The UV detector was operated at 254 nm, and a C18 column was used. The mobile phase consisted of methanol: acetic acid, and the flow rate was 0.2 ml/min. The elution profile was: 0.01 min 15% methanol, and 8 min 35% methanol; then, methanol was increased to 60% over 10 min, held for 15 min, and decreased to 15% over 16 min, held for 20 min.

### Degradation of the Mixture of PHBA and FA in Planted Soil

The CFA was incubated in LB liquid medium at 180 rpm and the optimized temperature (37°C) until OD_600_ = 1. Then, cells of the strain were collected into autoclaved water. Each cucumber seedling was cultivated in one pot containing 450 g of autoclaved soil (Wu et al., 2018). At the two-leaf stage, 32 cucumber seedlings were divided into four groups (8 plants per group). Each seedling in one group was watered with 25 ml of autoclaved water (control), cell suspension (8.04 log cfu/ml) of CFA (CFA treatment), solution containing PHBA (1.70 mg/ml) and FA (2.26 mg/ml) (PHBA + FA treatment), or solution containing CFA cells (8.04 log cfu/ml), PHBA (1.70 mg/ml), and FA (2.26 mg/ml) (CFA + PHBA + FA treatment). Subsequently, all seedlings were watered twice per day with autoclaved water (10 ml/plant each time) and cultivated at 25°C with a photoperiod of 12 h light (48,000 lux)/12 h dark. After 20 days of treatment, rhizospheric soil and the second leaves were collected from cucumber seedlings (Zhang et al., 2015). The concentrations of PHBA (Chen et al., 2015) and FA (Zhang et al., 2015) and the activities of CAT (Roberge, 1978), alkaline phosphatase (Anupama et al., 2008), and urease (Douglas and Bremner, 1971) were measured in rhizospheric soil. The levels of O_2_^⋅–^ (Elstner and Heupel, 1976), H_2_O_2_ (Bernt and Bergmeyer, 1974), and MDA (Dhindsa et al., 1981), the contents of AsA (Kampfenkel et al., 1995) and GSH (Guri, 1983), and the activities of SOD (Hwang et al., 1999), CAT (Pereira et al., 2002), APX (Zhu et al., 2004), GSH-Px (Xue et al., 2001), DHAR (Hoque et al., 2007), and MDHAR (Doulis et al., 1997) were assayed in cucumber leaves.

### Determination of Antioxidant Enzyme Activities in CFA

The strain CFA was inoculated into M-9 medium supplemented with the optimized concentration (0.2 g/l) of the mixture of PHBA and FA and incubated at 180 rpm and the optimized temperature (37°C). As controls, the strain was inoculated into M-9 medium supplemented with 85.6 mg/l PHBA, 114.4 mg/l FA, or 306.88 mg/l glucose (Wu et al., 2018). The activities of SOD (Hwang et al., 1999), CAT (Pereira et al., 2002), and DHAR (Hoque et al., 2007) were determined in CFA.

### Degradation Products of PHBA and FA by CFA

When being inoculated into M-9 medium supplemented with 0.2 g/l mixture of PHBA and FA, 85.6 mg/l PHBA, or 114.4 mg/l FA, CFA were incubated at 180 rpm and 37°C. The concentrations of degradation products of PHBA and FA were determined by using HPLC.

### RNA Sequencing

The CFA were inoculated into M-9 medium supplemented with 0.2 g/l mixture of PHBA and FA, 85.6 mg/l PHBA, 114.4 mg/l FA, and 306.88 mg/l glucose, respectively. When cells of the strain were incubated at 180 rpm and 37°C for 1.5 h, they were sent to LC Sciences (Hangzhou, China) for complete genome and mRNA sequencing. The complete genome sequence of CFA was generated by application of the Pacific Biosciences RS II (PacBio RS II) single-molecule real-time (SMRT) high-resolution sequence technology and assembly with the Hierarchical Genome Assembly Process (HGAP). RNA libraries of the strain were constructed using the Illumina Truseq RNA sample prep Kit. Then, samples were clustered (cBot Truseq PE Cluster Kit v3-cBot-HS) followed by sequencing (150 bp, Truseq SBS Kit v3-HS 200 cycles) on a Hiseq2000. Gene expression levels were calculated with the reads per kilobase of transcript per million reads mapped (RPKM) method. We used a negative binomial distribution to model the reads count. *p*-Value was estimated by using null hypothesis and Fisher exact test. A corrected *p*-value (*q*-value) was calculated using the false discovery rate (FDR). mRNAs with *q*-value < 0.05 and log_2_ fold-change (FC) > 1 were considered as differentially expressed. sRNAs were obtained by using a Rockhopper program. The sequence data of complete genome and transcriptome of CFA have been submitted to the GenBank database under accession number CP044546-CP044547 and SRR10267033-SRR10267044, respectively.

### Transcript Levels of Genes in the Pathways of PHBA and FA Degradation

The CFA was inoculated into M-9 medium supplemented with 0.2 g/l mixture of PHBA and FA, 85.6 mg/l PHBA, 114.4 mg/l FA, or 306.88 mg/l glucose and incubated at 180 rpm and 37°C. Transcript levels of genes in the pathways of PHBA and FA degradation were estimated using reverse transcription quantitative PCR (RT-qPCR) according to Wu et al. (2018). Primers of these genes are listed in Supplementary Table 2. The gene of RNA polymerase sigma factor (*rpoD*) was selected as an internal control. Three biological and three technical replicates were maintained in the real-time RT-PCR analysis.

### Gene Overexpression

Genes of feruloyl-CoA-synthetase (*fcs*), enoyl-CoA hydratase/aldolase (*ech*), vanillin dehydrogenase (*vdh*), vanillate *O*-demethylase (*vanAB*), and 4-hydroxybenzoate 3-monooxygenase (*pobA*) were amplified from CFA genomic DNA using the primers listed in Supplementary Table 2. Then, they were, respectively, inserted into pME6032 vector (Doberenz et al., 2017). After validation by colony PCR using the primers listed in Supplementary Table 2, the constructs were separately electrotransformed into CFA. To estimate whether the introduced constructs in CFA had the same copies, the transformed strains (6.61 log cfu) were used as templates, and quantitative PCR was performed with the primers listed in Supplementary Table 2. Subsequently, these strains overexpressing two copies of foreign *fcs*, *ech*, *vdh*, *vanAB*, or *pobA* were, respectively, inoculated into M-9 medium, where 1 mM IPTG was added and 0.2 g/l mixture of PHBA and FA was supplemented. The percentages of degraded PHBA and FA in medium were determined by using HPLC.

### sRNA Knockout

Target mRNAs of five sRNA genes (including *sRNA 8*, *sRNA11*, *sRNA 14*, *sRNA 20*, and *sRNA 60*) were predicted in http://rna.informatik.uni-freiburg.de/IntaRNA/Input.jsp. The upstream and downstream sequences of the sRNA genes were combined by overlap extension PCR using the primers listed in Supplementary Table 2. Following this, they were, respectively, inserted into pK18mobsacB (Roberts et al., 2012). The constructs were separately cloned into *Escherichia coli* S17-1 and conjugated into the recipient strain CFA. The recombination events in the recipient strain were selected by using 10% sucrose and validated by colony PCR with the primers listed in Supplementary Table 2. The mutant recipients, where *sRNA 8*, *sRNA 11*, *sRNA 14*, *sRNA 20*, or *sRNA 60* had been knocked out, were inoculated into M-9 medium supplemented with 0.2 g/l mixture of PHBA and FA. Then, they were incubated at 180 rpm and 37°C. The percentages of degraded PHBA and FA were determined using HPLC.

### Statistical Analysis

One-way analysis of variance (ANOVA) and the least significant difference (LSD) were conducted to analyze differences among treatments. *P*-values less than 0.05 were considered significant.

## Results

### Identification and Properties of CFA

Based on phylogenetic analysis of 16S rRNA gene sequences (Supplementary Figure 1) and sequence alignment of complete genomes, the strain CFA exhibited sequence identity with *Pseudomonas* and was identified as *Pseudomonas* sp. CFA. According to Supplementary Figure 2, CFA had PHBA- and FA-degrading abilities, and 0.5 g/l PHBA was completely degraded by the strain at 6 h. The highest percentages of degraded PHBA and FA by CFA were under conditions of 0.2–0.4 g/l mixture of PHBA and FA, 36–37°C, 0.75–1.5 g/l NH_4_C1, and pH 6 (Supplementary Figure 3). The strain CFA could also decompose the two phenolic acids at pH 7-9. Coefficient estimates of quadratic polynomial models for CFA (Supplementary Table 3) showed that the initial concentration of the mixture of PHBA and FA, temperature, and pH were variables correlated with the percentages of degraded PHBA and FA. Response surface plots (Supplementary Figure 4) indicated that the optimal values of the three variables were 0.2 g/l mixture of PHBA and FA, 37°C, and pH 6.56. When incubating CFA under the optimized conditions for 6 h, the percentages of degraded PHBA and FA by the strain were precisely analyzed using HPLC and were 100 and 63.77%, respectively.

### PHBA and FA Degradation in Soil and Their Effects on the Activities of Soil Enzymes in Rhizosphere and Antioxidant Enzymes in Cucumber

**FIGURE 2:**
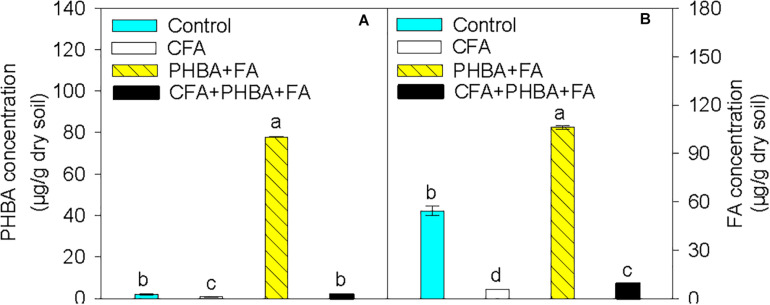
Effects of CFA and the mixture of PHBA and FA on the concentrations of PHBA **(A)** and FA **(B)** in cucumber rhizospheric soil. Control, supplemented with water; CFA, supplemented with CFA; PHBA + FA, supplemented with the mixture of PHBA and FA; CFA + PHBA + FA, supplemented with CFA and the mixture of PHBA and FA. Bars represent standard errors of triplicate experiments. Values with the different letters are significantly different at *P* < 0.05.

Compared to control conditions, the concentrations of PHBA (Figure 2A) and FA (Figure 2B) in rhizospheric soil were significantly decreased in the CFA treatment and obviously higher in the PHBA + FA treatment. In the CFA + PHBA + FA treatment in comparison to the PHBA + FA treatment, the concentrations of PHBA and FA in rhizospheric soil were significantly decreased.

**FIGURE 3:**
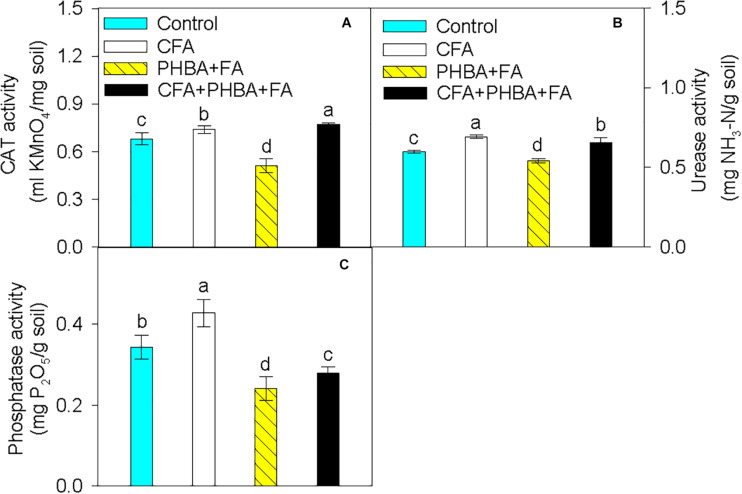
Effects of CFA and the mixture of PHBA and FA on the activities of CAT **(A)**, urease **(B)**, and phosphatase **(C)** in cucumber rhizospheric soil. Control, supplemented with water; CFA, supplemented with CFA; PHBA + FA, supplemented with the mixture of PHBA and FA; CFA + PHBA + FA, supplemented with CFA and the mixture of PHBA and FA. Bars represent standard errors of triplicate experiments. Values with the different letters are significantly different at *P* < 0.05.

In comparison to control conditions, the activities of CAT (Figure 3A), urease (Figure 3B), and phosphatase (Figure 3C) in rhizospheric soil were obviously increased in the CFA treatment and significantly decreased in the PHBA + FA treatment. When compared the CFA + PHBA + FA treatment with the PHBA + FA treatment, the activities of CAT, urease, and phosphatase were obviously increased in rhizospheric soil.

**FIGURE 4:**
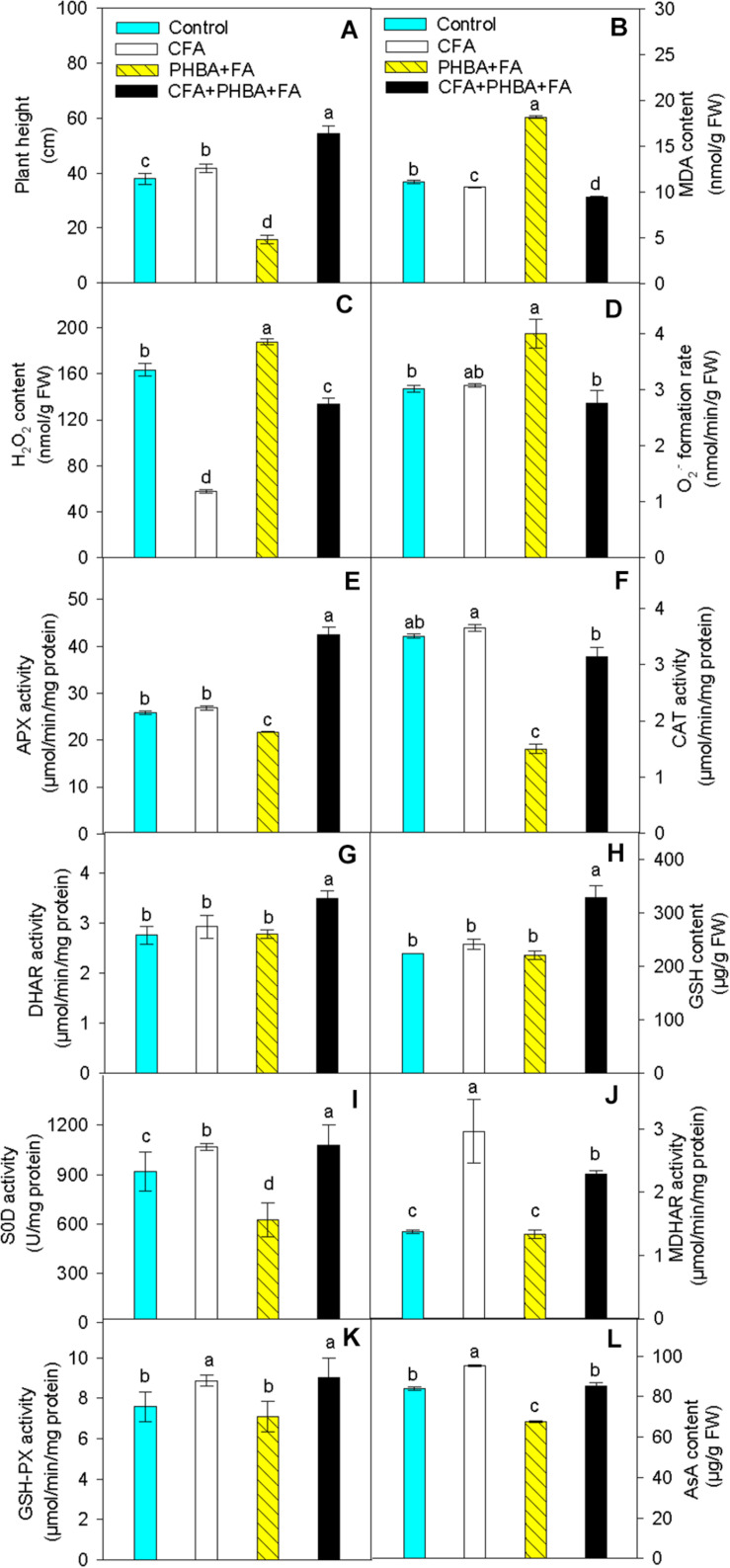
Effects of CFA and the mixture of PHBA and FA on plant height **(A)**, the levels of MDA **(B)**, H_2_O_2_ **(C)**, and O_2_^⋅–^ **(D)**, the activities of APX **(E)**, CAT **(F)**, DHAR **(G)**, the content of GSH **(H)**, the activities of SOD **(I)**, MDHAR **(J)**, and GSH-Px **(K)**, and the level of AsA **(L)** in cucumber leaves. Control, supplemented with water; CFA, supplemented with CFA; PHBA + FA, supplemented with the mixture of PHBA and FA; CFA + PHBA + FA, supplemented with CFA and the mixture of PHBA and FA. Bars represent standard errors of triplicate experiments. Values with the different letters are significantly different at *P* < 0.05.

In seedlings of the CFA treatment in comparison to control, the plant height of cucumber (Figure 4A) was higher, and the contents of MDA (Figure 4B) and H_2_O_2_ (Figure 4C) in leaves were significantly decreased. The level of O_2_^⋅–^ (Figure 4D), the activities of APX (Figure 4E), CAT (Figure 4F), and DHAR (Figure 4G), and the content of GSH (Figure 4H) in leaves did not change, while the activities of SOD (Figure 4I), MDHAR (Figure 4J), and GSH-Px (Figure 4K) and the level of AsA (Figure 4L) were obviously increased in leaves. When compared the PHBA + FA treatment with control, the plant height of cucumber, the content of AsA, and the activities of APX, CAT, and SOD in leaves were significantly decreased, and the levels of MDA, H_2_O_2_, and O_2_^⋅–^ were obviously increased in leaves, while the activities of DHAR and GSH-Px and the content of GSH in leaves had no significant difference. In comparison to the PHBA + FA treatment, the plant height of cucumber, the contents of AsA and GSH, and the activities of APX, CAT, DHAR, SOD, MDHAR, and GSH-Px in leaves were obviously increased in the CFA + PHBA + FA treatment, and the levels of MDA, H_2_O_2_, and O_2_^⋅–^ in leaves were obviously decreased.

### Effects of PHBA and FA on Antioxidant Enzymes in CFA

At 1.5 h, compared to the glucose treatment, the PHBA + FA treatment obviously decreased the activity of SOD (Supplementary Figure 5A), significantly increased the activity of CAT (Supplementary Figure 5B), and did not change the activity of DHAR (Supplementary Figure 5C) in CFA. The PHBA treatment significantly decreased the activities of SOD and DHAR and obviously increased the activity of CAT when compared to the glucose treatment, and the FA treatment significantly decreased the activity of SOD and obviously increased the activities of CAT and DHAR. At 2 h, the treatments of PHBA + FA, PHBA, and FA significantly increased the activities of SOD, CAT, and DHAR in comparison to the glucose treatment.

### Genome-Wide Transcriptome Profiles in Response to PHBA and FA

The genome of CFA consisted of a circular 5,935,153 bp chromosome (Supplementary Figure 6) and a circular 201,121 bp plasmid. Twenty-two rRNA genes, 75 tRNA genes, 241 ncRNA genes, and 5916 protein-coding genes were identified in the strain (Supplementary Table 4). Based on the RNA sequencing, transcriptome analysis revealed that 474, 546, and 334 genes in CFA being incubated for 1.5 h were differentially expressed in response to the mixture of PHBA + FA, PHBA, and FA, respectively (Supplementary Table 5). Compared to the glucose treatment at 1.5 h, genes of *fcs*, *ech*, *vdh*, and vanillate *O*-demethylase subunits (*vanA* and *vanB*) were upregulated in the treatments of PHBA + FA and FA, and the gene of *pobA* was upregulated in the treatments of PHBA + FA and PHBA (Table 1). The genes of protocatechuate 3,4-dioxygenase subunit (*pcaG* and *pcaH*), 3-oxoadipate enol-lactonase (*pcaD*), 3-oxoadipate CoA-transferase subunits (*pcaI* and *pcaJ*), beta-ketoadipyl CoA thiolase (*pcaF*), and acetyl-CoA acetyltransferase (*ACAT*) were upregulated in the FA treatment.

**TABLE 1:** Parts of differentially expressed genes in response to the mixture of PHBA and FA (PHBA + FA), PHBA, and FA by transcriptome sequencing.

Gene name	In response to PHBA + FA				In response to PHBA			In response to FA	
	Regulation type	Log_2_FC	FDR	Regulation type	Log_2_FC	FDR	Regulation type	Log_2_FC	FDR
Feruloyl-CoA-synthetase (*fcs*)	Up regulated	3.47	7.61E-30	–			Up regulated	3.21	9.63E-27
Enoyl-CoA hydratase/aldolase (*ech*)	Up regulated	5.42	5.23E-59	–			Up regulated	5.32	8.71E-58
Vanillin dehydrogenase (*vdh*)	Up regulated	4.53	1.40E-45	–			Up regulated	4.36	1.07E-43
Vanillate *O*-demethylase subunit (*vanA*)	Up regulated	3.11	5.20E-25	–			Up regulated	3.97	6.43E-36
Vanillate *O*-demethylase subunit (*vanB*)	Up regulated	3.07	1.87E-22	–			Up regulated	3.63	2.31E-31
4-Hydroxybenzoate 3-monooxygenase (*pobA*)	Up regulated	1.18	1.69E-04	Up regulated	1.01	3.62E-03	–		
Protocatechuate 3,4-dioxygenase subunit (*pcaG*)	–			–			Up regulated	1.62	3.53E-07
Protocatechuate 3,4-dioxygenase subunit (*pcaH*)	–			–			Up regulated	1.74	1.61E-08
3-Oxoadipate enol-lactonase (*pcaD*)	–			–			Up regulated	1.09	1.86E-03
3-Oxoadipate CoA-transferase subunit (*pcaI*)	–			–			Up regulated	1.34	4.03E-05
3-Oxoadipate CoA-transferase subunit (*pcaJ*)	–			–			Up regulated	1.14	1.01E-03
Beta-ketoadipyl CoA thiolase (*pcaF*)	–			–			Up regulated	1.42	5.84E-06
Acetyl-CoA acetyltransferase (*ACAT*)	–			–			Up regulated	2.18	5.05E-13

*Log_2_ FC, log_2_ fold-change. FDR, false discovery rate.*

To confirm the reliability of the transcriptome results, genes of *fcs*, *ech*, *vdh*, *vanA*, *vanB*, and *pcaD* were chosen. Their expression levels in CFA after incubation for 1.5 h were analyzed by RT-qPCR (Supplementary Figure 7). In comparison to the glucose treatment, the expression levels of *fcs*, *ech*, *vdh*, *vanA*, and *vanB* were higher in the treatments of PHBA + FA and FA, and the expression level of *pcaD* was increased in the FA treatment.

**FIGURE 5:**
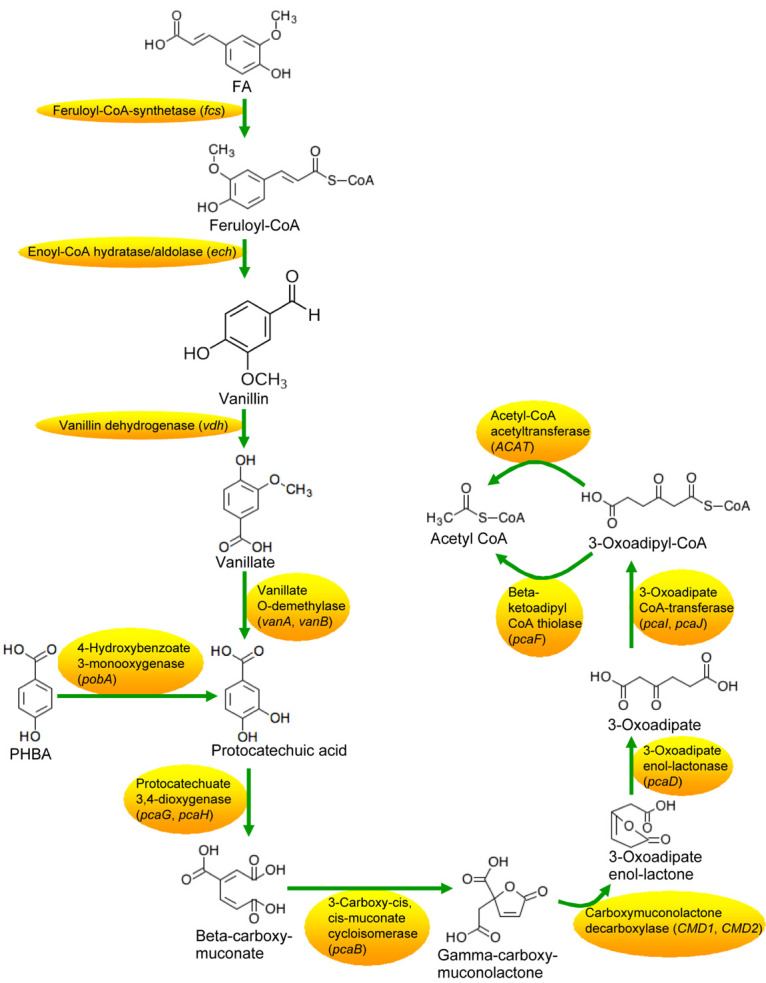
Proposed pathways of PHBA and FA degradation in CFA.

**FIGURE 6:**
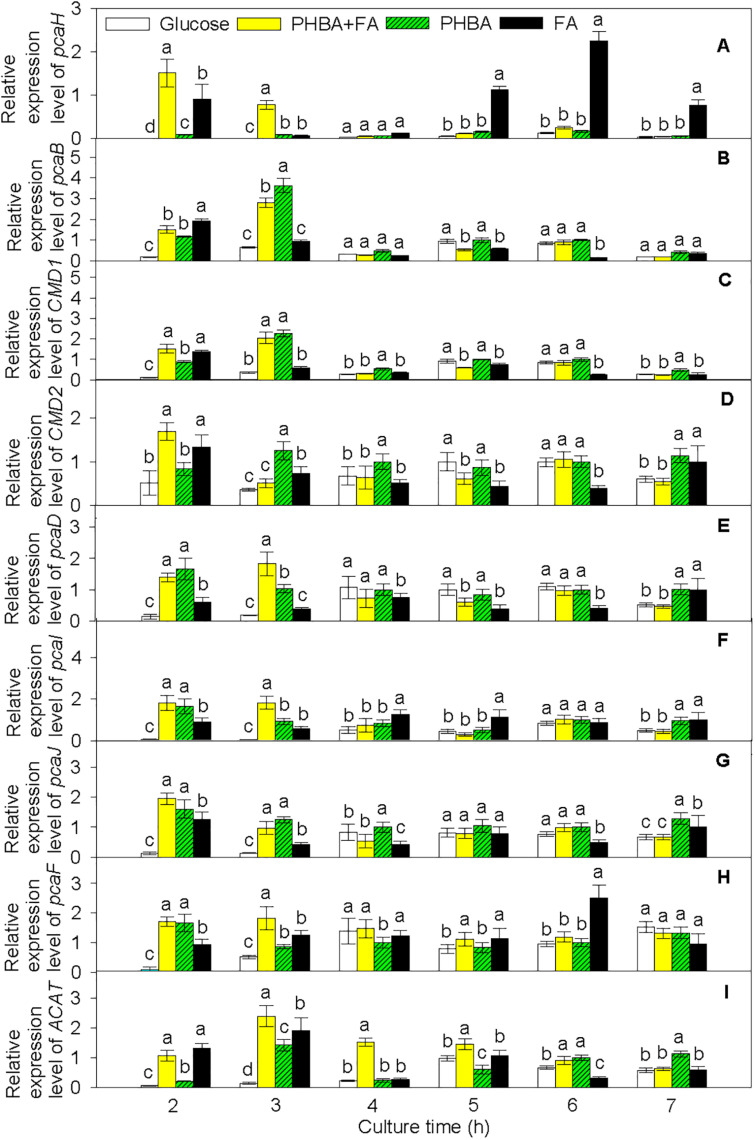
Relative expression levels of *pcaH* **(A)**, *pcaB* **(B)**, *CMD1* **(C)**, *CMD2* **(D)**, *pcaD* **(E)**, *pcaI* **(F)**, *pcaJ* **(G)**, *pcaF* **(H)**, and *ACAT* **(I)** in CFA. Glucose, cultured in glucose; PHBA + FA, cultured in the mixture of PHBA and FA; PHBA, cultured in PHBA; FA, cultured in FA. Bars represent standard errors of triplicate experiments. At each treatment time, values with the different letters are significantly different at *P* < 0.05.

Based on the transcriptome results, pathways of PHBA and FA degradation in CFA were proposed (Figure 5) and were then verified by RT-qPCR in the strain being incubated for 2–7 h (Figure 6 and Supplementary Figure 8). At 2 h, compared to the glucose treatment, the expression levels of *pcaH*, 3-carboxy-*cis*,*cis*-muconate cycloisomerase (*pcaB*), *pcaD*, *pcaI*, *pcaJ*, *pcaF*, *ACAT*, and carboxymuconolactone decarboxylase (*CMD1* and *CMD2*) were higher not only in the FA treatment, but also in the treatments of PHBA + FA and PHBA. The expression levels of *fcs*, *ech*, *vdh*, *vanA*, and *vanB* were significantly increased in the treatments of PHBA + FA and FA, and the expression level of *pobA* was obviously enhanced in the treatments of PHBA + FA and PHBA.

**FIGURE 7:**
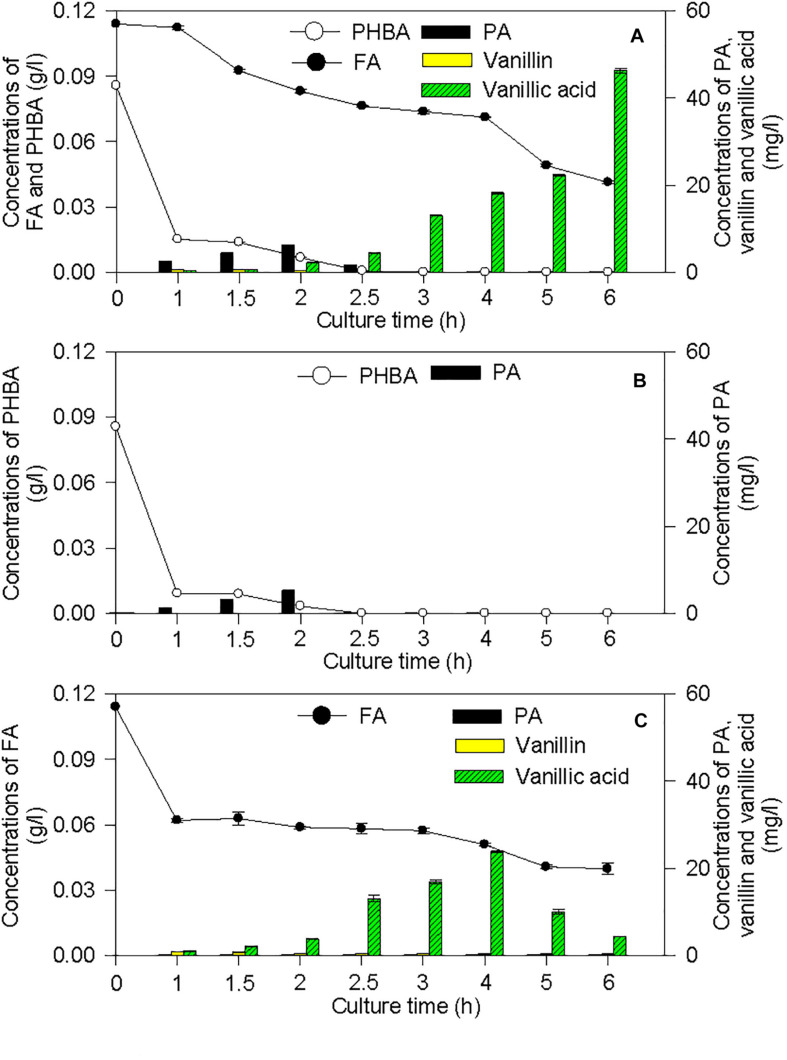
Bioconversion products from the mixture of PHBA and FA **(A)**, PHBA **(B)**, and FA **(C)** by CFA. Bars represent standard errors of triplicate experiments.

The proposed pathways of PHBA and FA degradation in CFA were also confirmed by the degradation products of PHBA and FA (Figure 7). The bioconversion products of the mixture of PHBA and FA contained vanillin, vanillic acid, and PA. According to Figures 7B,C, PA is a common product of PHBA and FA degradation. The metabolites of FA also had vanillin and vanillic acid. During the PHBA or FA degradation, the concentrations of PA, vanillin, and vanillic acid increased at first and then decreased. Accordingly, at 2.5 h, 99.29% PHBA was degraded by CFA, while 35.07% FA was degraded.

### Identification of Rate-Limiting Enzymes for the Bioconversion of PHBA and FA Into PA

**FIGURE 8:**
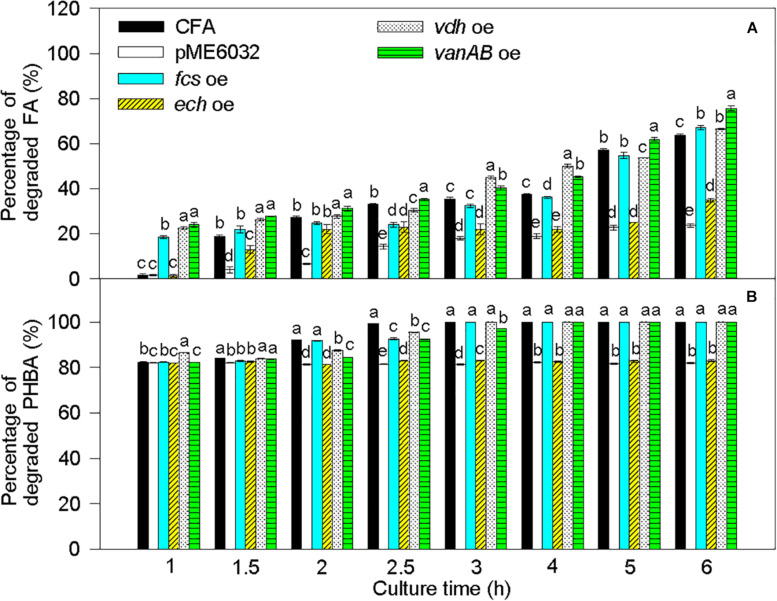
Percentages of degraded FA **(A)** and PHBA **(B)** in the mixture of PHBA and FA after overexpressing *fcs*, *ech*, *vdh*, and *vanAB*. CFA, wild type strain. pME6032, transformed with pME6032. *fcs* oe, transformed with pME6032-*fcs* and overexpressing *fcs*. *ech* oe, transformed with pME6032-*ech* and overexpressing *ech*. *vdh* oe, transformed with pME6032-*vdh* and overexpressing *vdh*. *vanAB* oe, transformed with pME6032-*vanA-vanB* and overexpressing *vanA* and *vanB*. Bars represent standard errors of triplicate experiments. At each treatment time, values with the different letters are significantly different at *P* < 0.05.

**FIGURE 9:**
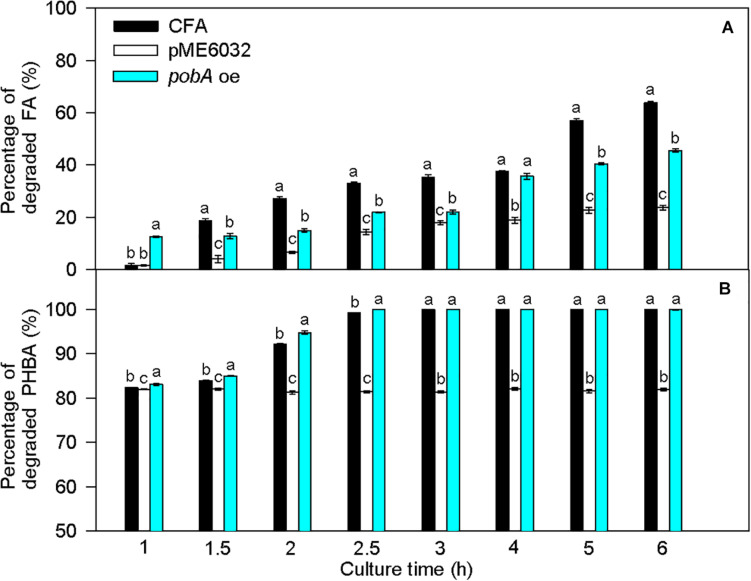
Percentages of degraded FA **(A)** and PHBA **(B)** in the mixture of PHBA and FA after overexpressing *pobA*. CFA, wild type strain. pME6032, transformed with pME6032. *pobA* oe, transformed with pME6032-*pobA* and overexpressing *pobA*. Bars represent standard errors of triplicate experiments. At each treatment time, values with the different letters are significantly different at *P* < 0.05.

Compared to untransformed CFA, the percentage of degraded FA was significantly increased by *fcs* overexpression at 1 and 6 h, by *vdh* overexpression at 1, 1.5, 2, 3, 4, and 6 h, and by *vanAB* overexpression at 1–6 h, and the percentage of degraded PHBA was obviously enhanced by *vdh* overexpression at 1 h and was not increased by overexpression of *fcs*, *ech*, and *vanAB* (Figure 8). Overexpression of *pobA* significantly increased the percentage of degraded FA at 1 h, and obviously elevated the percentage of degraded PHBA at 1–2.5 h (Figure 9). After being incubated for 3 h, both untransformed and *pobA*-overexpressed CFA made the percentage of degraded PHBA to be 100%. Therefore, vanAB and pobA are rate-limiting enzymes for the bioconversion of FA and PHBA, respectively.

### Effects of sRNA Knockout on PHBA and FA Degradation

**FIGURE 10:**
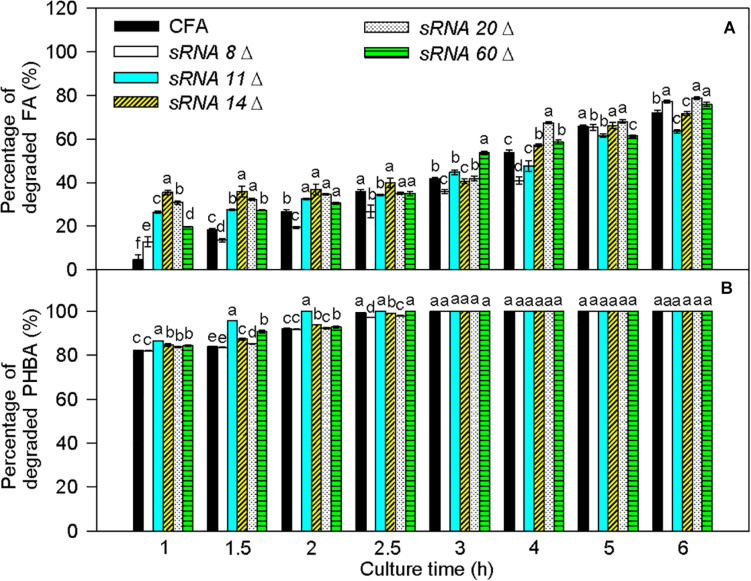
Percentages of degraded FA **(A)** and PHBA **(B)** in the mixture of PHBA and FA after knocking out *sRNA 8*, *sRNA 11*, *sRNA14*, *sRNA 20*, and *sRNA 60*. CFA, wild type strain without knocking out genes. *sRNA 8*Δ, knocking out *sRNA 8*. *sRNA 11*Δ, knocking out *sRNA 11*. *sRNA 14*Δ, knocking out *sRNA 14*. *sRNA 20*Δ, knocking out *sRNA 20*. *sRNA 60*Δ, knocking out *sRNA 60*. Bars represent standard errors of triplicate experiments. At each treatment time, values with the different letters are significantly different at *P* < 0.05.

Transcriptome results showed that sRNA 8, sRNA11, sRNA 14, sRNA 20, and sRNA 60 were small non-coding RNAs. Their sequences are listed in Supplementary Table 6, and they were predicted to interact with some mRNAs in the proposed PHBA and FA degradation pathways of CFA (Supplementary Figure 9). In comparison to untransformed CFA, the percentage of degraded FA was obviously increased by *sRNA 8* knockout at 1 and 6 h, by *sRNA 11* knockout at 1–2 h, by *sRNA 14* knockout at 1–2.5 and 4 h, by *sRNA 20* knockout at 1–2, 4, and 6 h, and by *sRNA 60* knockout at 1–2, 3–4, and 6 h (Figure 10). Meanwhile, the percentage of degraded PHBA was significantly elevated by *sRNA 11* knockout, *sRNA 16* knockout, and *sRNA 60* knockout at 1–2 h, obviously enhanced by *sRNA 20* knockout at 1–1.5 h, and did not change by *sRNA 8* knockout at 1–2 h. Therefore, knockout of *sRNA 8*, *sRNA 11*, *sRNA 14*, *sRNA 20*, or *sRNA 60* increases the percentage of degraded FA, and knockout of *sRNA11*, *sRNA 14*, *sRNA 20*, or *sRNA 60* elevates the percentage of degraded PHBA, especially at the early stage of PHBA and FA degradation.

## Discussion

### PHBA- and FA-Degrading Abilities of CFA

A strain CSY-P1 of *Pseudomonas* degrades 0.5 g/l PHBA in 8 h (Chen et al., 2015). Under the same culture conditions of CSY-P1, the strain CFA of *Pseudomonas* in this study decomposed the same amount of PHBA in 6 h, thus having higher PHBA-degrading ability. In the control group of this research, the FA concentration in cucumber rhizospheric soil reached 54.34 μg/g dry soil. The reason may be that the original soil collected for this study might contain high concentration of FA. When cucumber seedlings of this research were planted into this soil, roots could secrete this phenolic acid (Lu et al., 2019) and the concentration of FA increased further in the control. On the other hand, *Pseudomonas* sp. CFA, which was isolated from rhizospheric soil in the current experiment, could decompose a mixture of PHBA and FA in both medium and soil. As a result, the FA concentrations in the treatments of CFA and CFA + PHBA + FA were much lower than the control of this study. Similarly, a soil bacterium designated *Pseudomonas fluorescens* AN103 degrades FA in medium (Mohamed et al., 2020). These results suggest that CFA in this paper might have the potential for remediation of PHBA- and FA-contaminated soil, and it could be combined with phenolic acid-degrading strains reported by Razika et al. (2010) and other researchers to develop a bioremediation agent for mitigating continuous-cropping obstacles. Since 99.29% PHBA and 35.07% FA in 0.2 g/l mixture of PHBA and FA of this study were decomposed by CFA at 2.5 h, we propose that the strain CFA degrades PHBA preferentially. Moreover, pH values affect the degradation of benzoic acid and phenol by *P. aeruginosa* (Razika et al., 2010). In this study, CFA of *Pseudomonas* could decompose PHBA and FA at pH 6–9. These indicate that CFA can be recommended to soils having wide pH range. Using the strain CFA, we just obtained promising PHBA- and FA-degrading results in *in vitro* and pot culture experiments under controlled conditions. To utilize CFA effectively, a field experiment with lot of variables will be laid out to detect the phenolic acid-degrading ability of the strain in the future.

### Pathways of PHBA and FA Degradation by CFA

*Pseudomonas fluorescens* AN103 degrades FA into PA via feruloyl-CoA, vanillin, and vanillic acid (Narbad and Gasson, 1998). *P. putida* WCS35819 can decompose PHBA into PA directly (Venturi et al., 1998). PA can be decomposed into beta-carboxy-*cis*,*cis*-muconic acid (beta-carboxy-muconate) by *Pseudomonas* sp. HR199 (Overhage et al., 1999) and converted to β-ketoadipate by *P. putida* (Harwood and Parales, 1996). In this study, the complete pathways of PHBA and FA degradation were first proposed that CFA degraded PHBA and FA into PA as described by Narbad and Gasson (1998) and Venturi et al. (1998) and then decomposed PA to acetyl CoA via beta-carboxy-muconate and β-ketoadipate. However, results of transcriptome and RT-PCR in the current experiment showed that, during PHBA degradation by CFA, *pobA* has been transcribed at 1.5 h, while genes of *pcaH*, *pcaB*, *CMD1*, *CMD2*, *pcaD*, *pcaI*, *pcaJ*, *pcaF*, and *ACAT* did not express at 1.5 h and were transcribed at 2 h. When CFA in this study degrading PHBA, the concentration of PA increased at first and reached a peak at 2 h. These results indicate that CFA of this paper might result in transient accumulation of PA at the early stage of PHBA degradation. During PHBA or FA degradation by CFA in this study, the concentrations of PA, vanillin, and vanillic acid increased at first, but then decreased. Thus, we propose that no phenolic products will accumulate to a certain concentration that are toxic to plants (Jung et al., 2004) when CFA in the current experiment is applied to degrade the mixture of PHBA and FA in planted soil.

### Effects of Rate-Limiting Enzymes and sRNA on PHBA and FA Degradation by CFA

When PHBA and FA being degraded into PA by CFA in this study, pobA and vanAB were rate-limiting enzymes, respectively. This can be supported by the report that vanAB is a rate-limiting enzyme to catalyze vanillic acid to PA (Buswell and Ribbons, 1988). Overexpression of *pobA* and *vanAB* separately promoted the degradation of PHBA and FA in the current experiment. Similarly, Takemura et al. (2010) improve alkaloid productivity by overexpressing rate-limiting enzymes. In addition, *sRNA* knockout also increased the percentages of degraded PHBA and FA in this study, especially at the early stage of PHBA and FA degradation. Kim et al. (2015) found the similar result that knockout of Hfq-associated sRNA modulates the antibiotic susceptibility of bacteria. So rate-limiting enzymes and sRNA were first found to affect PHBA and FA degradation in CFA.

### Effect of Antioxidant Enzymes in CFA on PHBA and FA Degradation by the Strain

During PHBA and FA degradation by CFA in this study, the strain was exposed to PHBA and FA conditions. Since phenolic compounds will cause secondary oxidative stresses in cells (Imatomi et al., 2013), CFA might be subjected to this kind of stress caused by PHBA and FA in medium. To respond to the oxidative stress, the antioxidant enzymes (including SOD, CAT, and DHAR) in CFA of the current experiment were induced at 2 h by the PHBA + FA, PHBA, and FA treatments. Similarly, the activities of multiple antioxidant enzymes in nicotine-degrading *Pseudomonas* sp. HF-1 are increased under oxidative stress caused by nicotine (Shao et al., 2009). The activated antioxidant enzymes in CFA of this study might enhance the PHBA and FA tolerance of the strain and contribute to the degradation of the two phenolic compounds by CFA (Chen et al., 2015; Zhang et al., 2015). It is similar to the report that antioxidant compounds in *Burkholderia xenovorans* improve polychlorobiphenyl degradation by the strain (Ponce et al., 2011).

### PHBA and FA Stress Mitigation in Cucumber by CFA

Stress environments induce the production of reactive oxygen species (ROS) including H_2_O_2_, and O_2_^⋅–^ in cells, while the overproduction of ROS can damage lipid membrane (Sun et al., 2012) and inhibit plant growth (Shah et al., 2020). Meantime, MDA is a product of membrane lipid peroxidation (Qu et al., 2020), and its content is increased under stresses (Li et al., 2013). In the PHBA + FA treatment in comparison to control of this study, the levels of MDA, H_2_O_2_, and O_2_^⋅–^ in leaves were increased and the plant height of seedlings was decreased. The reason may be that the mixture of PHBA and FA in soil causes oxidative stress in cucumber (Imatomi et al., 2013) by inducing ROS such as H_2_O_2_ and O_2_^⋅–^ (Wu et al., 2019). As a result, MDA is produced and plant height is inhibited. On the other hand, when being inoculated into PHBA- and FA-supplemented soil, CFA in the current experiment decreased the PHBA and FA concentrations in soil, increased the plant height of cucumber, and reduced the levels of MDA, H_2_O_2_, and O_2_^⋅–^ in leaves. These results suggest that PHBA- and FA-caused oxidative stress (Imatomi et al., 2013) is mitigated in cucumber due to PHBA and FA degradation by CFA, thus decreasing MDA content and increasing plant height.

### Effect of Antioxidant Enzymes in Cucumber on PHBA and FA Stress Mitigation by CFA

The antioxidant enzymes APX, CAT, and GSH-Px remove H_2_O_2_, and SOD dismutates O_2_^⋅–^ into H_2_O_2_. Meantime, AsA acts as a substrate of APX and is regenerated by MDHAR or GSH-dependent DHAR (Sun et al., 2012). In the PHBA + FA treatment in comparison to control in this study, the activities of APX, CAT, and SOD were decreased in leaves and activities of DHAR, MDHAR, and GSH-Px did not change, which was different from the results that PHBA and FA in medium induced antioxidant enzymes in CFA. The reason may be that the stress caused by PHBA and FA in soil leads to more severe oxidative damage to antioxidant enzymes in cucumber, thus, the activities of enzymes begin to reduce gradually (Bharwana et al., 2013). When CFA was applied into PHBA- and FA-supplemented soil in this paper, we obtained the enhanced activities of APX, CAT, DHAR, SOD, MDHAR, and GSH-Px in leaves. These results were proved by the increased contents of AsA and GSH in seedlings in the CFA + PHBA + FA treatment in comparison to the PHBA + FA treatment of this study, in accordance with the reduced levels of O_2_^⋅–^ and H_2_O_2_ in leaves, consistent with the decreased concentrations of PHBA and FA in soil, and in conjunction with the improved plant height. Wu et al. (2018) also observed the increased activities of antioxidant enzymes in PHBA- and FA-stressed cucumber by using a PHBA- and FA-degrading strain of *Acinetobacter*. Jain et al. (2020) found that plant growth-promoting bacteria induce antioxidant enzymes and improve plant growth under zinc conditions. These suggest that CFA in this study degrades PHBA and FA in soil and reduces the PHBA- and FA-caused oxidative damage to antioxidant enzymes, thus increasing the activities of antioxidant enzymes in leaves, decreasing ROS levels, improving plant growth, and mitigating PHBA and FA stresses in cucumber.

### Effect of Soil Enzymes on PHBA and FA Stress Mitigation by CFA

The activity of soil enzyme is a sensitive indicator of soil health (Dussault et al., 2008). In heavy metal-polluted soil, the activities of soil enzymes such as CAT, urease, and phosphatase were inhibited (Hu et al., 2014), showing the toxic impact of heavy metal contaminats (Dussault et al., 2008). In PHBA- and FA-contaminated soil in this study, the decreased activities of CAT, urease, and phosphatase were observed, suggesting that PHBA and FA might also have adverse effects on soil health. When CFA in this paper being inoculated into PHBA- and FA-contaminated soil, the activities of CAT, urease, and phosphatase in rhizosphere were increased. One of the reasons might be that the concentrations of PHBA and FA in soil were decreased due to the PHBA- and FA-degrading abilities of CFA, hence, the adverse effects of PHBA and FA on soil health were reduced. Similarly, PHBA- and FA-degrading *Streptomyces canus* GLY-P2 enhances soil enzyme activities in cucumber soil, where PHBA and FA are added (Wu et al., 2019). Moreover, soil health affects plant growth (Pulavarty et al., 2020). Inoculation with *P. fluorescens* and *Bacillus cereus* activates soil enzymes and improves growth of *Taxus chinensis* (Bai et al., 2020). The enhanced activities of soil enzymes caused by CFA in this study were in accordance with the reduced levels of MDA in cucumber when compared the CFA + PHBA + FA treatment to the PHBA + FA treatment and consistent with the increased plant height. We thereby propose that, due to having the PHBA- and FA-degrading abilities, CFA in the current experiment can improve plant growth and mitigate PHBA and FA stresses in cucumber by inducing soil enzymes in rhizosphere.

## Conclusion

Using transcriptome analysis, CFA converts PHBA and FA to PA and then to acetyl CoA. *pobA* and *vanAB* are rate-limiting enzyme genes for the bioconversion of PHBA and FA, respectively. Knockout of *sRNA 11*, *sRNA 14*, *sRNA 20*, or *sRNA 60* elevates the percentages of degraded PHBA and FA, and sRNA affects the PHBA and FA degradation. When being applied to cucumber-planted soil where PHBA and FA are supplemented, CFA mitigates PHBA and FA stresses in cucumber by activating antioxidant enzymes in leaves and inducing soil enzymes in rhizosphere as the strain decomposes PHBA and FA in soil.

## Data Availability Statement

The datasets generated for this study can be found in the GenBank accession number MH558575, CP044546-CP044547, and SRR10267033-SRR10267044.

## Author Contributions

J-GB defined the research theme and designed the methods and experiments. H-PF wrote the manuscript. YZ, C-XC, and X-JW co-designed the experiments and discussed analyses. UR and RW revised the manuscript. Z-YC, Y-QA, and BD co-worked on associated data collection and their interpretation. All authors contributed to the article and approved the submitted version.

## Conflict of Interest

The authors declare that the research was conducted in the absence of any commercial or financial relationships that could be construed as a potential conflict of interest.
