# Supplementary material for: Transcriptome Profiling Combined With Activities of Antioxidant and Soil Enzymes Reveals an Ability of Pseudomonas sp. CFA to Mitigate p-Hydroxybenzoic and Ferulic Acid Stresses in Cucumber
Source: Front Microbiol. 2020 Oct 27;11:522986. doi: 10.3389/fmicb.2020.522986 (PMC7652996; doi:10.3389/fmicb.2020.522986)
Supplement: Supplementary file 1 [file Data_Sheet_1.PDF]

Supplementary materials

TABLE S1 Levels and codes of independent variables for the Box-Behnken design

Original factors	Symbol		Coded levels		
	Uncoded	Coded	-1	0	1
Initial concentration of the mixture of FA and PHBA (g/l)	X_1	x_1	0.1	0.4	0.7
Temperature (℃)	X_2	x_2	35	37	39
pH	X_3	x_3	5.5	6.5	7.5

TABLE S2 Primers used in this study

Gene name	Primer sequence (5'-3')	Annealing temperatures (℃)
**For quantitative PCR assays**		
fcs	F: GAGCTTGGCCTGATGTTC	55
	R: CCCGACACTGACGAATAC	
ech	F: CCTCCAGGAAAAGATTCGT	55
	R: ATGGTCGGTTTGGCATAC	
vdh	F: CAACTGGTATGGCTTCAAC	55
	R: GTGATCTGCGTGGTCATG	
vanA	F: GACAACCTCATGGACCTC	55
	R: TGCTTCGTCGATCTCCTT	
vanB	F: TTCTCACCGAAGAGGAAC	55
	R: TCAGATATCCAGCACCAG	
pcaH	F: ATATCCACTTCGCCATCA	55
	R: TTGACGATAGGGCACATC	
pcaB	F: TTGAAGCACGCCGATAC	55
	R: TTTCATGCCCAGGGTCA	
CMD1	F: GATCTACTGCGGCATTCC	55
	R: TCTACGCCAAGTTCATCC	
CMD2	F: AGCAATGAAAAGTACGAAAAG	55
	R: AATTCATCTGCGTTCTGG	
pcaD	F: ACATTCAAAAGGCTCACTTC	55
	R: CTCACCTGCATGGATACC	
pcaI	F: CAAGGAAACCCGTGAGAT	55
	R: CTTGATCAGTGCGAAGTC	

pcaJ	F: TACTGGAAGTGACACCTGAA	55
	R: CACGCCACTGAGTTTCTG	
pcaF	F: CGCAACATGAAGCTGGAAG	55
	R: CACACCGTACTGGCTCTTC	
ACAT	F: GGGTTCAAGGACTTTCTCT	55
	R: GTTTTCCGCAGTACCGAT	
pobA	F: ATGAATTGAAAGGCGAGAAG	55
	R: GATATAGTCGCAGTCAATCC	
rpoD	F: TTCGGTATCGACATGAACAC	55
	R: TCGCTTCGATCTGACGGAT	
**For overexpression vector construction of genes**		
fcs	F: CGGAATTCGTGAATAACGAAGCCCGCTC	68.0
	R: GAAGATCTTCAAGGCCGCACCTTGGCG	
ech	F: CGGAATTCATGAGCAAATATGAAGGCCG	64.0
	R: CCCTCGAGTCAGCGCTTGTAGGCCT	
vdh	F: CGGAATTCATGTTGCAGGTGCCTTTGCT	64.0
	R: CCCTCGAGCTAGATGGGATAGTGACGCG	
vanAB	F: CGGAATTCATGCACCCCAAAAACACCTGGTACG	69.0
	R: CCCTCGAGTCAGATATCCAGCACCAGCA	
pobA	F: CGGAATTCATGAAAACTCAGGTTGCA	59.0
	R: GAAGATCTTCAGGTCACTTCTTCGAACGG	
**For validation of overexpressed genes**		
fcs	F: GTGTGGAATTGTGAGCGGATAACAATTT	59.0
	R: AGCATCTGCACGTAGCTGATCGAA	
ech	F: GTGTGGAATTGTGAGCGGATAACAATTT	58.0
	R: CTTTTCCTGGAGGATTTCCGGG	

vdh	F: GTGTGGAATTGTGAGCGGATAACAATTT	58.0
	R: CCAACTTCACGTTGAAGCCATACCA	
vanAB	F: GTGTGGAATTGTGAGCGGATAACAATTT	59.0
	R: CCAGGTTGCCGTCCTCGACATA	
pobA	F: GTGTGGAATTGTGAGCGGATAACAATTT	58.0
	R: CAGGGGCTTTGAGATCCAGACGCT	
**For knockout vector construction of *sRNA* genes**		
Left arm of *sRNA8*	F: CGGAATTCCTACATTTGTTTCGGGCCAGTC	64.0
	R: CGGCCATGCGCTGCGGGAGGGGTTTTTTTTGTGTTTTTGT	
Right arm of *sRNA8*	F: ACAAAAACACAAAAAAAACCCCTCCCGCAGCGCATGGCCG	68.0
	R: GCTCTAGAGTTGCCCATACGCACGCCA	
Left arm of *sRNA11*	F: CGGAATTCGCAGGCAGTCGGCATATTC	65.0
	R: CGCGGCAAGCGAACCATGTGTCATGGGGTCGGCTTGACAT	
Right arm of *sRNA11*	F: ATGTCAAGCCGACCCCATGACACATGGTTCGCTTGCCGCG	68.0
	R: GCTCTAGACTCCATCTCCGGTGCGACGATC	
Left arm of *sRNA14*	F: GCTCTAGATCGACCTGCCGGGCTATGGT	68.0
	R: ATGGCTGGTGAAGCCCTTCGATTTGCCGGATGGCTTGTAC	
Right arm of *sRNA14*	F: GTACAAGCCATCCGGCAAATCGAAGGGCTTCACCAGCCAT	65.0
	R: CCCAAGCTTCGGACTATTCACAGATCGAGC	
Left arm of *sRNA20*	F: GCTCTAGAAACGTGCGGTTCATCGATTC	62.0
	R: CTTCATTAGCCTGAAGAGCCAATGAATTTTCGCTAATCCG	
Right arm of *sRNA20*	F: CGGATTAGCGAAAATTCATTGGCTCTTCAGGCTAATGAAG	63.0
	R: CCCAAGCTTTTACAGGCATGTTGAAGCG	
Left arm of *sRNA60*	F: CGGAATTCCTGAGTCTTGGATCACGTCTA	62.0
	R: CGTTGTCCTCATGTGCTCGCCTTGTTTGACAGCAAGGTTG	
Right arm of *sRNA60*	F: CAACCTTGCTGTCAAACAAGGCGAGCACATGAGGACAACG	66.0

	R: GCTCTAGAACAACGCCGCAACCGGATTTG	
**For validating knockout vector of sRNA genes**		
sRNA8	F: CGGGTTCTGGGGAGGAATC	59.0
	R: GTTGCCCATACGCACGCC	
sRNA11	F: GCAGGCAGTCGGCATATTCGT	58.0
	R: ACCCTCGTATTATTGTGTTTTGGGTTGA	
sRNA14	F: TCCGGGCAAAAAAAACCCC	57.0
	R: CGGACTATTCACAGATCGAGCTGC	
sRNA20	F: AACGTGCGGTTCATCGATT	52.0
	R: AAAAAAAAGACCCGGCAAAAAA	
sRNA60	F: CTGAGTCTTGGATCACGTCTAGCAAG	59.0
	R: TTTCATGGCGGCTGTACGTG	

TABLE S3 Analysis of variance (ANOVA) for the quadratic model evaluation of the percentages of degraded PHBA and FA

Source	Coefficient	Sum of squares	Mean square	F-value	Prob > F
**For percentage of degraded PHBA**					
Model		10692.58	1188.06	70.51	< 0.0001**
Intercept	81.34				
X_1 (Initial concentration of the mixture of PHBA and FA)	-17.62	2483.93	2483.93	147.41	< 0.0001**
X_2 (Temperature)	15.69	1970.65	1970.65	116.95	< 0.0001**
X_3 (pH)	-8.00	511.49	511.49	30.36	0.0009**
X_1X_2	11.64	542.28	542.28	32.18	0.0008**
X_1X_3	-2.67	28.47	28.47	1.69	0.2348
X_2X_3	0.83	2.77	2.77	0.16	0.6974
X_1^2	-2.47	25.66	25.66	1.52	0.2570*
X_2^2	-31.52	4182.35	4182.35	248.21	< 0.0001**
X_3^2	-12.68	676.85	676.85	40.17	0.0004**
Lack of fit		97.37	2.968E-003	6.31	0.0536
**For percentage of degraded FA**					
Model		10748.28	1194.25	52.51	< 0.0001**
Intercept	79.85				
X_1 (Initial concentration of the mixture of PHBA and FA)	-17.91	2566.38	2566.38	112.83	< 0.0001**
X_2 (Temperature)	6.28	315.76	315.76	13.18	0.0074*
X_3 (pH)	-8.86	628.54	628.54	27.63	0.0012*
X_1X_2	-0.44	0.76	0.76	0.033	0.8602
X_1X_3	-9.07	328.72	328.72	14.45	0.0067*
X_2X_3	12.24	599.12	599.12	26.34	0.0014*
X_1^2	-21.83	2006.26	2006.26	88.21	< 0.0001**

X_2^2	-25.90	2824.03	2824.03	124.16	< 0.0001**
X_3^2	-14.28	858.51	858.51	37.74	0.0005**
Lack of fit		105.06	35.02	2.59	0.1905

For percentage of degraded PHBA, R^2 = 0.9891; Adj R^2 = 0.9751; Pred R^2 = 0.8529.

For percentage of degraded FA, R^2 = 0.9854; Adj R^2 = 0.9666; Pred R^2 = 0.8381.

*, significant at the 5% level; **, significant at the 1% level.

TABLE S4 Genome statistics

Attribute	Value	% of Total
Genome size (bp)	6136274	100.00
DNA G + C (bp)	3785706	61.69
DNA coding (bp)	5313939	86.60
Total genes	6254	100.00
Protein-coding genes	5916	94.60
RNA genes	338	5.40
rRNA genes	22	0.35
tRNA genes	75	1.20
ncRNA genes	241	3.85
TRF repeats	276	-
SSR repeats	34	-
CRISPR repeats	16	-
Genes with function prediction	5025	84.94
Genes assigned to NR	3785	63.98
Genes assigned to Swiss-prot	4006	67.71
Genes assigned to KEGG	3102	52.43
Genes assigned to GO	3085	52.15
Genes assigned to COGs	4874	82.39
N^6-Methyladenosine (m6A)	759	-
N^4-methylcytosine (m4C)	328993	-
Genes involved in restriction-modification systems	185	3.68
Histidine protein kinase	59	1.17
Response regulator protein	85	1.69
Genes with signal peptides	523	10.41
Genes with transmembrane structure	1297	25.81

TABLE S5 Differentially expressed genes in response to the mixture of PHBA and FA, PHBA, and FA by transcriptome sequencing

Sequence name	Log_2FC	p-Value	FDR	Regulated	Function
**In response to the mixture of PHBA and FA**					
orf05164-3526	5.415523	8.73E-63	5.23E-59	up	Enoyl-CoA hydratase/aldolase
orf05167-3527	4.525267	4.67E-49	1.40E-45	up	Vanillin dehydrogenase
orf05140-3507	-4.2426	1.55E-43	3.10E-40	down	_
orf08123-5571	-3.78333	2.92E-36	4.38E-33	down	_
orf03474-2355	3.632814	2.28E-34	2.73E-31	up	_
orf08122-5570	-3.6439	4.10E-34	4.10E-31	down	_
orf05168-3528	3.471504	8.90E-33	7.61E-30	up	Feruloyl-CoA-synthetase
orf03477-2357	3.465188	3.30E-32	2.47E-29	up	_
orf04369-2968	3.325175	3.46E-30	2.13E-27	up	Hypothetical protein, conserved
orf08126-5573	-3.35805	3.56E-30	2.13E-27	down	Transcriptional regulator
orf06277-4326	3.284907	8.00E-30	4.36E-27	up	_
orf04372-2969	3.262541	1.92E-28	9.57E-26	up	_
orf06515-4494	3.260153	8.82E-28	4.06E-25	up	GGDEF domain/EAL domain protein
orf06284-4331	3.278175	1.19E-27	5.10E-25	up	Putative periplasmic binding protein
orf06510-4491	3.114602	1.30E-27	5.20E-25	up	Vanillate O-demethylase subunit
orf05139-3506	-3.17148	5.90E-27	2.21E-24	down	D-glycerate dehydrogenase
orf08124-5572	-3.07243	1.46E-26	5.15E-24	down	_
orf08138-5580	-3.04494	3.98E-26	1.32E-23	down	Sugar ABC transporter substrate-binding protein
orf08130-5576	-3.0647	4.77E-26	1.50E-23	down	ABC transporter substrate-binding protein
orf08129-5575	-3.01432	1.85E-25	5.55E-23	down	_
orf05255-3600	3.492376	2.28E-25	6.49E-23	up	_

orf06511-4492	3.074927	6.87E-25	1.87E-22	up	Vanillate O-demethylase subunit
orf07989-5471	2.951098	3.97E-24	1.03E-21	up	Membrane protein
orf02689-1835	2.812722	5.94E-23	1.48E-20	up	3-Hydroxyisobutyrate dehydrogenase
orf08145-5585	-2.79229	9.01E-23	2.16E-20	down	Phosphogluconate dehydratase
orf00378-256	2.789055	2.92E-22	6.71E-20	up	_
orf05135-3503	-2.89594	3.11E-22	6.90E-20	down	AP endonuclease
orf05181-3537	2.793482	4.62E-22	9.88E-20	up	Diguanylate cyclase
orf06882-4763	2.773481	6.03E-22	1.24E-19	up	_
orf06276-4325	2.695159	3.71E-21	7.41E-19	up	Aldehyde dehydrogenase
orf02688-1834	2.662148	4.22E-21	8.14E-19	up	_
orf01381-949	-2.84554	4.86E-21	9.10E-19	down	Hypothetical protein YSA_05068
orf07566-5196	2.620922	1.79E-20	3.24E-18	up	Acetyl-coenzyme A synthetase 1
orf03476-2356	2.765074	5.43E-20	9.57E-18	up	_
orf07009-4845	2.572585	1.26E-19	2.16E-17	up	Uncharacterised protein
orf06514-4493	2.617807	1.44E-19	2.40E-17	up	Vanillate O-demethylase subunit
orf02275-1552	2.56611	1.76E-19	2.85E-17	up	_
orf02759-1883	-2.61544	1.84E-19	2.89E-17	down	_
orf06946-4801	2.533981	3.14E-19	4.82E-17	up	_
orf05174-3532	2.484041	7.49E-19	1.12E-16	up	Protein of unknown function
orf04347-2952	2.524497	9.75E-19	1.42E-16	up	Citrate synthase/methylcitrate synthase
orf04546-3087	2.508484	1.24E-18	1.77E-16	up	Monosaccharide-transporting ATPase
orf06944-4800	2.518212	1.91E-18	2.66E-16	up	Gamma-carboxygeranoyl-CoA hydratase
orf00729-504	-2.47496	2.03E-18	2.76E-16	down	50S ribosomal protein L10
orf05467-3753	2.551502	2.69E-18	3.57E-16	up	3-Oxoacid CoA-transferase subunit B
orf05136-3504	-2.49728	5.21E-18	6.78E-16	down	_
orf06283-4330	2.645655	7.16E-18	9.12E-16	up	Amino acid ABC transporter substrate-binding protein

orf06943-4799	2.395716	1.82E-17	2.27E-15	up	Propionyl-CoA carboxylase
orf00704-485	-2.40054	2.14E-17	2.62E-15	down	_
orf05170-3529	2.377758	2.70E-17	3.24E-15	up	Acetyl-CoA acetyltransferase
orf08299-5687	2.414021	2.81E-17	3.30E-15	up	_
orf00727-503	-2.4306	3.11E-17	3.58E-15	down	_
orf07128-4922	-2.33754	4.94E-17	5.59E-15	down	_
orf00703-484	-2.34673	1.29E-16	1.43E-14	down	50S ribosomal protein L24
orf00702-483	-2.30244	1.68E-16	1.83E-14	down	_
orf00947-643	2.273224	2.59E-16	2.76E-14	up	_
orf00705-486	-2.32481	4.34E-16	4.56E-14	down	_
orf07126-4921	-2.25162	6.67E-16	6.88E-14	down	Succinate--CoA ligase subunit alpha
orf03165-2145	2.514647	9.22E-16	9.36E-14	up	_
orf05131-3500	-2.26695	1.21E-15	1.21E-13	down	_
orf02760-1884	-2.26812	1.55E-15	1.52E-13	down	Membrane protein
orf06281-4329	2.251825	3.60E-15	3.47E-13	up	_
orf00701-482	-2.2182	6.57E-15	6.25E-13	down	30S ribosomal protein S14
orf07130-4924	-2.1559	7.89E-15	7.38E-13	down	_
orf05468-3754	2.204787	1.08E-14	9.97E-13	up	_
orf00926-629	-2.18401	1.18E-14	1.07E-12	down	_
orf00706-487	-2.17838	1.53E-14	1.36E-12	down	50S ribosomal protein L29
orf01738-1196	2.139326	2.03E-14	1.79E-12	up	Putative adenosylmethionine-8-amino-7–oxo-nonanoate aminotransferase
orf04482-3044	2.24808	2.64E-14	2.29E-12	up	_
orf00708-489	-2.12303	2.69E-14	2.30E-12	down	_
orf01367-939	2.09566	3.11E-14	2.60E-12	up	MFS transporter
orf05004-3411	2.530233	3.13E-14	2.60E-12	up	Hypothetical protein, conserved
orf02866-1947	2.127433	3.91E-14	3.20E-12	up	_

orf05036-3431	-2.07699	8.66E-14	7.01E-12	down	_
orf07570-5199	2.123122	8.95E-14	7.15E-12	up	_
orf05177-3534	2.03389	1.22E-13	9.61E-12	up	_
orf05128-3498	-2.19861	1.58E-13	1.23E-11	down	GMC family oxidoreductase
orf07129-4923	-2.03304	1.80E-13	1.38E-11	down	_
orf07525-5168	2.058669	2.01E-13	1.52E-11	up	_
orf06941-4798	2.055603	2.21E-13	1.65E-11	up	Isovaleryl-CoA dehydrogenase
orf00718-497	-2.01346	2.25E-13	1.66E-11	down	Elongation factor Tu
orf07010-4846	2.028431	2.41E-13	1.76E-11	up	Hypothetical protein DW66_3957
orf08127-5574	-2.06751	2.47E-13	1.78E-11	down	_
orf07312-5038	2.162655	3.77E-13	2.69E-11	up	_
orf00707-488	-2.03075	4.12E-13	2.90E-11	down	_
orf04381-2976	2.12344	4.41E-13	3.06E-11	up	_
orf01981-1357	-2.00356	4.45E-13	3.06E-11	down	Glutamine synthetase
orf04548-3088	2.023752	5.30E-13	3.59E-11	up	ABC transporter related protein
orf08143-5584	-2.0268	5.34E-13	3.59E-11	down	_
orf08139-5581	-2.00389	8.55E-13	5.69E-11	down	_
orf07522-5167	1.99215	1.21E-12	7.97E-11	up	Branched-chain alpha-keto acid dehydrogenase subunit E2
orf06857-4747	1.974634	1.47E-12	9.57E-11	up	Isocitrate dehydrogenase
orf06456-4455	1.96368	1.68E-12	1.08E-10	up	Hydroperoxidase, partial
orf00730-505	-1.94289	2.07E-12	1.32E-10	down	_
orf00781-539	1.953933	2.09E-12	1.32E-10	up	_
orf00732-506	-1.93948	2.40E-12	1.50E-10	down	_
orf06280-4328	1.959206	2.70E-12	1.66E-10	up	_
orf08134-5578	-2.21793	3.10E-12	1.89E-10	down	_
orf06885-4764	1.950934	4.04E-12	2.44E-10	up	Zn-dependent hydrolase

orf00709-490	-1.94831	5.49E-12	3.29E-10	down	50S ribosomal protein L22
orf05178-3535	1.908178	5.95E-12	3.53E-10	up	_
orf01368-940	1.889976	8.13E-12	4.78E-10	up	_
orf05129-3499	-1.90715	8.27E-12	4.81E-10	down	_
orf04750-3240	1.915171	1.08E-11	6.21E-10	up	Hypothetical protein
orf02867-1948	1.993551	1.20E-11	6.84E-10	up	Hypothetical protein DW66_4785
orf02668-1822	-1.85255	1.40E-11	7.89E-10	down	_
orf04508-3061	2.00282	1.47E-11	8.24E-10	up	Hypothetical protein
orf02260-1542	-1.85034	1.75E-11	9.71E-10	down	Membrane protein
orf03827-2601	1.922368	1.88E-11	1.03E-09	up	_
orf00710-491	-1.86559	2.62E-11	1.42E-09	down	_
orf02276-1553	2.118757	2.89E-11	1.56E-09	up	_
orf06286-4333	1.835067	3.36E-11	1.79E-09	up	Quinoprotein ethanol dehydrogenase PedE
orf07132-4925	-1.81003	3.39E-11	1.80E-09	down	Hypothetical protein AW09_001448
orf00365-246	-1.86088	3.46E-11	1.82E-09	down	Ribosomal protein L21
orf01479-1015	-1.8094	3.99E-11	2.08E-09	down	Pyruvate carboxylase
orf03451-2337	1.835576	4.30E-11	2.22E-09	up	_
orf08147-5586	-1.82152	4.59E-11	2.31E-09	down	Glyceraldehyde-3-phosphate dehydrogenase
orf05172-3530	1.814457	4.60E-11	2.31E-09	up	_
orf00711-492	-1.80235	4.62E-11	2.31E-09	down	50S ribosomal protein L2
orf07794-5340	-1.91381	4.63E-11	2.31E-09	down	_
orf05137-3505	-1.83861	4.77E-11	2.36E-09	down	Putative 2-ketogluconate kinase
orf00927-630	-1.80699	4.89E-11	2.38E-09	down	Dihydrolipoamide acetyltransferase
orf04349-2953	1.813815	4.89E-11	2.38E-09	up	Fe/S-dependent 2-methylisocitrate dehydratase AcnD
orf00206-137	-2.03919	5.94E-11	2.85E-09	down	_
orf05003-3410	1.789917	5.94E-11	2.85E-09	up	Hypothetical protein YSA_11298

orf07782-5333	-1.80622	6.13E-11	2.91E-09	down	Malate:quinone oxidoreductase
orf03511-2381	1.832408	8.59E-11	4.05E-09	up	Pyrroloquinoline quinone (Coenzyme PQQ) biosynthesis protein C-like protein
orf02257-1540	-1.78381	8.91E-11	4.17E-09	down	_
orf08136-5579	-1.78917	1.08E-10	5.01E-09	down	_
orf00376-255	1.998925	1.16E-10	5.33E-09	up	Hypothetical protein YSA_06429
orf00952-646	1.74996	1.32E-10	5.99E-09	up	_
orf06285-4332	1.769511	1.32E-10	5.99E-09	up	_
orf00925-628	-1.75075	1.42E-10	6.38E-09	down	_
orf05074-3460	-1.82351	1.43E-10	6.38E-09	down	_
orf00169-117	-1.75407	1.69E-10	7.50E-09	down	Cytochrome ubiquinol oxidase subunit I
orf00719-498	-1.73666	1.78E-10	7.85E-09	down	Elongation factor G
orf00209-139	-1.79651	1.80E-10	7.86E-09	down	1-Phosphofructokinase
orf00311-211	-1.75798	2.21E-10	9.57E-09	down	_
orf00018-14	-1.73575	2.72E-10	1.17E-08	down	_
orf06458-4456	1.805217	2.79E-10	1.19E-08	up	Hydroperoxidase, partial
orf00248-170	1.743749	3.19E-10	1.35E-08	up	_
orf02690-1836	1.852538	3.65E-10	1.54E-08	up	Cupin
orf00810-558	1.730577	3.74E-10	1.57E-08	up	_
orf01477-1014	-1.70602	4.74E-10	1.97E-08	down	_
orf04884-3329	1.730462	4.80E-10	1.98E-08	up	_
orf00949-645	1.722668	5.64E-10	2.31E-08	up	Sarcosine oxidase subunit gamma
orf04378-2974	1.800629	5.97E-10	2.43E-08	up	_
orf00720-499	-1.69322	6.49E-10	2.63E-08	down	30S ribosomal protein S7
orf04407-2992	1.744767	7.49E-10	3.01E-08	up	_
orf05072-3459	-1.69823	9.66E-10	3.86E-08	down	_
orf04557-3094	1.804315	1.13E-09	4.46E-08	up	Hypothetical protein

orf04552-3090	1.715074	1.27E-09	5.01E-08	up	_
orf07986-5470	1.726373	1.33E-09	5.20E-08	up	_
orf08133-5577	-1.73254	1.34E-09	5.20E-08	down	Hypothetical protein RHECNPAF_1360097
orf03578-2426	-1.73584	1.37E-09	5.28E-08	down	_
orf02742-1871	1.687265	1.41E-09	5.42E-08	up	_
orf00724-501	-1.64065	1.57E-09	6.00E-08	down	Uncharacterised protein
orf02258-1541	-1.70666	1.83E-09	6.95E-08	down	30S ribosomal protein S18
orf01737-1195	1.65135	2.06E-09	7.76E-08	up	Glutamine synthetase, partial
orf00214-141	-1.63436	2.10E-09	7.86E-08	down	PTS fructose transporter subunit IIA
orf02261-1543	-1.6547	2.16E-09	8.04E-08	down	_
orf05126-3497	-1.68857	2.18E-09	8.08E-08	down	_
orf04346-2951	1.700251	2.30E-09	8.42E-08	up	Methylisocitrate lyase
orf00713-494	-1.63046	2.31E-09	8.42E-08	down	_
orf01063-726	1.692493	2.48E-09	8.98E-08	up	LysM domain/BON superfamily protein
orf00882-599	-1.65641	2.73E-09	9.84E-08	down	Biotin synthase
orf05737-3933	-1.65227	3.50E-09	1.26E-07	down	Saccharopine dehydrogenase
orf05433-3728	1.698287	3.60E-09	1.28E-07	up	_
orf03159-2141	1.61743	4.53E-09	1.61E-07	up	_
orf07571-5200	1.724928	4.60E-09	1.62E-07	up	_
orf00380-258	1.651777	5.03E-09	1.76E-07	up	_
orf01414-969	-1.5886	5.80E-09	2.02E-07	down	ATP synthase F1 sector subunit beta
orf08161-5596	1.609687	5.88E-09	2.04E-07	up	_
orf02478-1699	-1.85026	5.99E-09	2.06E-07	down	_
orf06938-4796	1.785595	7.60E-09	2.60E-07	up	_
orf00166-115	-1.68394	8.76E-09	2.98E-07	down	_
orf05173-3531	1.628003	8.82E-09	2.98E-07	up	_

orf04211-2861	1.603999	9.41E-09	3.16E-07	up	Acetyl-coenzyme A synthetase
orf00714-495	-1.56095	9.69E-09	3.24E-07	down	_
orf00207-138	-1.56854	1.05E-08	3.50E-07	down	PTS system fructose-specific transporter subunits IIBC
orf03132-2124	-1.55891	1.28E-08	4.23E-07	down	_
orf06179-4252	1.55399	1.38E-08	4.54E-07	up	Uncharacterised protein
orf04553-3091	1.609072	1.42E-08	4.65E-07	up	_
orf01412-968	-1.55915	1.46E-08	4.74E-07	down	_
orf05449-3739	1.617508	1.57E-08	5.09E-07	up	_
orf00691-473	-1.55382	1.60E-08	5.15E-07	down	_
orf04383-2977	1.629258	1.71E-08	5.49E-07	up	_
orf04355-2957	-1.54307	1.76E-08	5.59E-07	down	_
orf00998-681	1.534744	1.78E-08	5.65E-07	up	_
orf04209-2860	1.561804	1.88E-08	5.93E-07	up	Acetyl-CoA acetyltransferase
orf00722-500	-1.55539	1.98E-08	6.22E-07	down	Hypothetical protein K151_2921
orf06892-4768	1.553781	2.28E-08	7.11E-07	up	Dihydropyrimidine dehydrogenase subunit B
orf07823-5363	-1.53007	2.35E-08	7.30E-07	down	_
orf04256-2893	1.639158	2.81E-08	8.66E-07	up	_
orf02481-1702	1.671201	2.86E-08	8.77E-07	up	_
orf00693-475	-1.49313	3.67E-08	1.12E-06	down	_
orf05641-3863	1.667676	3.75E-08	1.14E-06	up	Hypothetical protein
orf03615-2456	-1.62958	3.88E-08	1.17E-06	down	_
orf00712-493	-1.52372	4.16E-08	1.25E-06	down	_
orf03286-2222	1.538475	4.32E-08	1.29E-06	up	S-formylglutathione hydrolase
orf01296-891	1.650987	4.42E-08	1.32E-06	up	Hypothetical protein YSA_05171
orf01911-1314	-1.49528	4.47E-08	1.32E-06	down	_
orf00167-116	-1.54295	4.77E-08	1.41E-06	down	Cytochrome o ubiquinol oxidase

orf05222-3570	1.567792	5.24E-08	1.54E-06	up	_
orf00364-245	-1.56477	5.35E-08	1.56E-06	down	_
orf03599-2443	-1.50655	5.37E-08	1.56E-06	down	_
orf05270-3610	1.480973	5.45E-08	1.58E-06	up	_
orf00577-395	1.475727	5.55E-08	1.60E-06	up	Aldehyde dehydrogenase B (wide specificity)
orf00687-470	-1.47243	5.61E-08	1.61E-06	down	_
orf00689-471	-1.47644	5.86E-08	1.67E-06	down	_
orf04953-3376	1.500945	5.98E-08	1.70E-06	up	_
orf05069-3456	1.732232	6.28E-08	1.77E-06	up	Hypothetical protein T1E_5212
orf02972-2017	-1.4688	6.81E-08	1.91E-06	down	Pyruvate kinase
orf08160-5595	1.470738	7.28E-08	2.04E-06	up	_
orf02405-1639	1.764405	7.35E-08	2.05E-06	up	_
orf03284-2221	1.484122	8.09E-08	2.24E-06	up	_
orf03167-2146	1.464938	8.89E-08	2.45E-06	up	Spermidine/putrescine ABC transporter substrate- binding protein
orf00726-502	-1.43917	1.02E-07	2.80E-06	down	DNA-directed RNA polymerase subunit beta
orf06889-4766	1.457316	1.08E-07	2.96E-06	up	_
orf03314-2245	1.524178	1.10E-07	2.99E-06	up	_
orf07521-5166	1.481219	1.11E-07	2.99E-06	up	2-Oxoisovalerate dehydrogenase subunit beta
orf01930-1326	-1.45023	1.17E-07	3.16E-06	down	_
orf00690-472	-1.44247	1.23E-07	3.31E-06	down	_
orf00736-509	-1.42786	1.29E-07	3.44E-06	down	Elongation factor Tu
orf03133-2125	-1.46677	1.29E-07	3.44E-06	down	_
orf07266-5007	1.485338	1.55E-07	4.09E-06	up	_
orf06296-4342	1.447504	1.86E-07	4.90E-06	up	Hypothetical protein PPS11_11936
orf03598-2442	-1.42079	1.90E-07	5.00E-06	down	_
orf02281-1557	1.464439	2.04E-07	5.34E-06	up	_

orf00250-171	1.418512	2.11E-07	5.49E-06	up	Hypothetical protein
orf01586-1094	1.419162	2.36E-07	6.13E-06	up	Aldehyde dehydrogenase
orf06275-4324	1.555442	2.49E-07	6.42E-06	up	_
orf01528-1050	1.46184	2.68E-07	6.86E-06	up	Integration host factor
orf07541-5179	-1.41256	2.68E-07	6.86E-06	down	Aspartate kinase
orf04351-2954	1.427847	2.96E-07	7.52E-06	up	_
orf05447-3738	1.441075	2.97E-07	7.52E-06	up	_
orf07316-5041	1.440198	3.10E-07	7.84E-06	up	_
orf07564-5195	2.26427	3.17E-07	7.98E-06	up	_
orf05037-3432	-1.60511	3.72E-07	9.32E-06	down	Hypothetical protein, conserved
orf00686-469	-1.42082	3.90E-07	9.74E-06	down	_
orf04155-2825	1.363141	4.00E-07	9.93E-06	up	_
orf07041-4866	-1.39252	4.23E-07	1.05E-05	down	_
orf00036-26	1.382043	4.38E-07	1.08E-05	up	Dipeptide ABC transporter periplasmic dipeptide- binding protein
orf03131-2123	-1.38944	4.52E-07	1.11E-05	down	_
orf01726-1187	1.4902	4.58E-07	1.12E-05	up	Hypothetical protein
orf01744-1199	1.389695	4.89E-07	1.19E-05	up	_
orf08030-5507	-1.37045	5.49E-07	1.33E-05	down	_
orf01491-1023	-1.37118	5.57E-07	1.34E-05	down	_
orf04206-2858	1.366065	6.55E-07	1.57E-05	up	AMP-binding protein
orf04882-3328	1.415362	6.57E-07	1.57E-05	up	LysR family transcriptional regulator
orf07040-4865	-1.35925	7.20E-07	1.72E-05	down	NADH:ubiquinone oxidoreductase subunit M
orf02611-1788	-1.38807	8.07E-07	1.92E-05	down	_
orf06292-4338	1.356056	8.16E-07	1.93E-05	up	_
orf04550-3089	1.356294	8.24E-07	1.94E-05	up	_
orf00694-476	-1.33907	8.33E-07	1.96E-05	down	_

orf03158-2140	1.466607	8.48E-07	1.98E-05	up	DNA repair protein HhH-GPD
orf06940-4797	1.327157	9.14E-07	2.13E-05	up	_
orf07315-5040	1.350746	9.44E-07	2.19E-05	up	Hypothetical protein ALO46_102909
orf06742-4658	-1.34071	9.88E-07	2.28E-05	down	Hypothetical protein
orf02896-1969	-1.33467	1.12E-06	2.58E-05	down	_
orf00716-496	-1.3385	1.16E-06	2.65E-05	down	30S ribosomal protein S10
orf06289-4336	1.577621	1.16E-06	2.65E-05	up	Hypothetical protein, conserved
orf01409-966	-1.33477	1.16E-06	2.65E-05	down	_
orf07265-5006	1.347814	1.18E-06	2.67E-05	up	Cytochrome c oxidase, cbb3-type subunit I
orf03529-2391	-1.3041	1.27E-06	2.88E-05	down	_
orf04032-2737	1.31134	1.32E-06	2.98E-05	up	Aconitate hydratase
orf01230-839	-1.38633	1.34E-06	3.01E-05	down	_
orf00809-557	1.326031	1.36E-06	3.04E-05	up	Hypothetical protein
orf03833-2605	-1.3887	1.37E-06	3.05E-05	down	Hypothetical protein RK21_03449
orf01009-688	1.446083	1.38E-06	3.05E-05	up	Hypothetical protein YSA_05583
orf06891-4767	1.319432	1.47E-06	3.24E-05	up	Dihydropyrimidine dehydrogenase subunit A
orf00150-5826	1.542627	1.55E-06	3.41E-05	up	_
orf03406-2307	-1.46194	1.64E-06	3.59E-05	down	Methyl-accepting chemotaxis sensory transducer
orf07960-5453	1.306448	1.65E-06	3.60E-05	up	_
orf02283-1558	1.342737	1.66E-06	3.60E-05	up	_
orf02243-1531	-1.30614	1.66E-06	3.60E-05	down	_
orf07311-5037	1.306276	1.66E-06	3.60E-05	up	_
orf07461-5130	-1.32585	1.71E-06	3.69E-05	down	_
orf06750-4665	1.346585	1.79E-06	3.85E-05	up	_
orf06518-4496	1.477751	1.86E-06	3.97E-05	up	Hypothetical protein RK21_01659
orf02566-1759	1.299974	1.97E-06	4.19E-05	up	L-lactate permease

orf03522-2387	-1.30437	2.09E-06	4.44E-05	down	P-protein
orf00692-474	-1.34851	2.14E-06	4.52E-05	down	_
orf04208-2859	1.335585	2.26E-06	4.77E-05	up	_
orf00558-382	1.268751	2.27E-06	4.78E-05	up	_
orf04379-2975	1.338762	2.32E-06	4.86E-05	up	_
orf01410-967	-1.27721	2.42E-06	5.05E-05	down	_
orf02326-1585	-1.27912	2.50E-06	5.19E-05	down	_
orf05654-3873	-1.42928	2.59E-06	5.37E-05	down	RNA polymerase subunit sigma-70
orf05460-3746	1.33758	2.60E-06	5.37E-05	up	_
orf02045-1400	1.280593	2.61E-06	5.38E-05	up	Poly(hydroxyalkanoate) granule-associated protein
orf03244-2196	-1.27169	2.87E-06	5.88E-05	down	Elongation factor Ts
orf05049-3442	1.342191	3.04E-06	6.22E-05	up	Thiazole biosynthesis protein ThiJ
orf05599-3836	-1.26798	3.23E-06	6.58E-05	down	_
orf07310-5036	1.279631	3.34E-06	6.77E-05	up	Xanthine dehydrogenase small subunit
orf06814-4715	1.261901	3.38E-06	6.84E-05	up	_
orf03526-2389	-1.25759	3.52E-06	7.10E-05	down	_
orf06856-4746	1.512046	3.56E-06	7.16E-05	up	_
orf00953-647	1.308397	3.64E-06	7.30E-05	up	Sarcosine oxidase subunit delta
orf01295-890	1.234163	3.67E-06	7.33E-05	up	Cytochrome b559 subunit alpha
orf02872-1951	1.314914	3.72E-06	7.40E-05	up	_
orf02246-1533	1.252507	3.96E-06	7.86E-05	up	_
orf07051-4873	1.322219	4.14E-06	8.18E-05	up	_
orf04109-2788	-1.38091	4.38E-06	8.62E-05	down	_
orf04555-3092	1.349059	4.46E-06	8.75E-05	up	_
orf03304-2236	1.261997	5.02E-06	9.83E-05	up	_
orf04450-3020	1.282005	6.14E-06	0.00012	up	_

orf02280-1556	1.238388	6.26E-06	0.000122	up	Branched-chain amino acid ABC transporter permease
orf05592-3832	1.250196	6.32E-06	0.000123	up	_
orf00227-5882	1.330082	6.34E-06	0.000123	up	_
orf00038-28	1.455147	6.82E-06	0.000131	up	_
orf04777-3259	1.248358	7.15E-06	0.000137	up	Amino acid ABC transporter
orf03242-2194	-1.21173	7.38E-06	0.000141	down	_
orf04323-2939	-1.22786	7.54E-06	0.000143	down	Phospho-2-dehydro-3-deoxyheptonate aldolase
orf00956-649	1.213505	7.56E-06	0.000143	up	_
orf00807-556	1.207842	7.56E-06	0.000143	up	_
orf06302-4347	1.203121	8.48E-06	0.00016	up	Putative two-component sensor
orf05253-3599	1.206186	8.62E-06	0.000162	up	Acetyl-CoA acetyltransferase
orf07373-5078	-1.24164	8.88E-06	0.000167	down	_
orf04645-3164	-1.23058	9.02E-06	0.000169	down	Transcriptional regulator
orf04878-3325	1.176774	9.06E-06	0.000169	up	4-Hydroxybenzoate 3-monooxygenase
orf06301-4346	1.272147	9.43E-06	0.000175	up	_
orf00697-478	-1.19307	9.70E-06	0.000179	down	_
orf05258-3603	1.352295	9.71E-06	0.000179	up	PaaH
orf04862-3314	-1.21315	9.78E-06	0.00018	down	Hybrid sensor histidine kinase/response regulator
orf04952-3375	1.215482	1.03E-05	0.000188	up	Acyl-CoA dehydrogenase
orf03130-2122	-1.24734	1.03E-05	0.000189	down	RNA-binding protein
orf00007-5703	-1.32578	1.05E-05	0.000191	down	_
orf08142-5583	-1.22383	1.09E-05	0.000198	down	_
orf00971-662	1.1674	1.11E-05	0.000201	up	Electron transfer flavoprotein subunit alpha
orf00695-477	-1.21183	1.12E-05	0.000201	down	_
orf08163-5597	1.259287	1.12E-05	0.000201	up	Acetyl-CoA carboxylase
orf02301-1570	1.307136	1.12E-05	0.000201	up	_

orf01004-685	-1.22573	1.12E-05	0.000202	down	Imidazole glycerol phosphate synthase subunit HisH
orf02700-1843	1.192285	1.18E-05	0.00021	up	Gamma-glutamyltranspeptidase
orf05843-4008	1.206168	1.18E-05	0.00021	up	_
orf01416-970	-1.22511	1.18E-05	0.00021	down	_
orf02737-1869	1.297748	1.23E-05	0.000217	up	Diguanylate phosphodiesterase
orf02055-1406	1.191278	1.25E-05	0.00022	up	_
orf03301-2234	1.253265	1.37E-05	0.000241	up	_
orf06288-4335	1.20218	1.41E-05	0.000248	up	Hypothetical protein PPUTLS46_000315
orf00449-307	1.167242	1.42E-05	0.000249	up	Chaperone protein ClpB
orf03857-2620	-1.18647	1.43E-05	0.00025	down	_
orf05459-3745	1.225066	1.50E-05	0.000261	up	_
orf03685-2505	1.239182	1.54E-05	0.000267	up	OsmC family protein
orf02967-2014	1.24485	1.55E-05	0.000269	up	_
orf05259-3604	1.114038	1.72E-05	0.000297	up	_
orf01749-1202	1.188682	1.76E-05	0.000302	up	Hypothetical protein PPUTLS46_018104
orf07876-5400	1.203582	1.76E-05	0.000302	up	_
orf01908-1312	-1.20649	1.86E-05	0.000318	down	Ribosomal protein L31
orf07037-4864	-1.16085	1.97E-05	0.000337	down	_
orf00556-381	1.172738	2.08E-05	0.000355	up	Fis family GAF modulated sigma54 specific transcriptional regulator
orf01927-1325	-1.14132	2.31E-05	0.000392	down	_
orf07267-5008	1.303123	2.33E-05	0.000395	up	_
orf05134-3502	-1.24157	2.42E-05	0.000409	down	_
orf06858-4748	-1.13577	2.49E-05	0.000419	down	Isocitrate dehydrogenase
orf00210-140	-1.23859	2.59E-05	0.000434	down	1-Phosphofructokinase
orf06566-4528	1.189476	2.69E-05	0.000449	up	_
orf07892-5410	-1.13059	2.75E-05	0.000459	down	Ribonucleotide-diphosphate reductase subunit alpha

orf00666-455	1.167251	2.81E-05	0.000468	up	_
orf00524-360	1.183286	2.94E-05	0.000488	up	_
orf00579-396	1.143561	2.95E-05	0.000488	up	_
orf00592-403	-1.145	2.99E-05	0.000493	down	_
orf00275-187	-1.23198	3.07E-05	0.000505	down	_
orf02125-1454	1.132083	3.08E-05	0.000505	up	Two-component system response regulator
orf03751-2550	1.128868	3.23E-05	0.000528	up	Acyl-CoA dehydrogenase
orf05736-3932	-1.16111	3.32E-05	0.000542	down	_
orf01747-1201	1.149203	3.47E-05	0.000565	up	Putrescine/spermidine ABC transporter permease
orf07011-4847	1.205758	3.50E-05	0.000568	up	Short-chain dehydrogenase
orf05141-3508	-1.202	3.63E-05	0.000587	down	_
orf00165-114	-1.14275	3.64E-05	0.000588	down	_
orf07036-4863	-1.22271	3.74E-05	0.000602	down	NADH-quinone oxidoreductase subunit K
orf00976-664	1.11766	3.81E-05	0.000612	up	_
orf05742-3936	1.204051	3.95E-05	0.000633	up	_
orf02580-1769	1.101241	3.99E-05	0.000637	up	_
orf00955-648	1.103176	4.03E-05	0.000643	up	Hypothetical protein ALO70_04556
orf03581-2428	-1.12496	4.05E-05	0.000643	down	_
orf02606-1785	-1.10294	4.19E-05	0.000664	down	Translation initiation factor IF-2
orf07749-5315	1.246137	4.40E-05	0.000695	up	_
orf04157-2826	1.098363	4.45E-05	0.000701	up	Formate dehydrogenase subunit alpha
orf06931-4792	1.10821	4.64E-05	0.000729	up	Uncharacterised protein
orf03837-2609	-1.10614	4.66E-05	0.00073	down	Aspartate-semialdehyde dehydrogenase
orf00698-479	-1.11065	4.67E-05	0.000731	down	_
orf03528-2390	-1.11142	4.69E-05	0.000732	down	_
orf03157-2139	1.122097	4.70E-05	0.000732	up	_

orf01742-1198	1.119226	4.76E-05	0.000739	up	_
orf06297-4343	1.125811	4.81E-05	0.000744	up	_
orf07519-5165	1.1159	4.86E-05	0.00075	up	_
orf05163-3525	1.111244	5.15E-05	0.000791	up	_
orf05672-3886	1.553195	5.15E-05	0.000791	up	Short-chain dehydrogenase
orf07603-5220	-1.15575	5.38E-05	0.000823	down	Spy-related protein
orf02279-1555	1.149417	5.40E-05	0.000825	up	_
orf05441-3734	1.1252	6.03E-05	0.000919	up	_
orf04213-2862	1.125643	6.09E-05	0.000925	up	Enoyl-CoA hydratase
orf05257-3602	1.057758	6.21E-05	0.000941	up	Phenylacetate-CoA oxygenase subunit PaaA
orf01142-780	1.147537	6.42E-05	0.000971	up	Histidine kinase
orf02404-1638	1.149397	6.47E-05	0.000976	up	_
orf01984-1359	-1.07832	6.75E-05	0.001015	down	_
orf00968-659	1.082263	6.83E-05	0.001024	up	_
orf00979-666	1.07258	6.89E-05	0.001032	up	Hypothetical protein PSA5_11350
orf03854-2619	-1.08	6.96E-05	0.001039	down	Amidophosphoribosyltransferase
orf06304-4348	1.056597	7.13E-05	0.001063	up	_
orf06462-4459	-1.09761	7.22E-05	0.001072	down	_
orf02876-1955	-1.07533	7.26E-05	0.001075	down	_
orf05434-3729	1.105813	7.43E-05	0.001099	up	_
orf00970-661	1.044891	7.65E-05	0.001128	up	Electron transfer flavoprotein subunit beta
orf07983-5468	1.152688	7.81E-05	0.001146	up	_
orf05718-3920	1.093701	7.82E-05	0.001146	up	_
orf01006-686	-1.11195	7.82E-05	0.001146	down	Imidazoleglycerol-phosphate dehydratase
orf06887-4765	1.04625	7.97E-05	0.001164	up	_
orf06751-4666	1.197832	8.04E-05	0.001172	up	_

orf05005-3412	1.067509	8.12E-05	0.00118	up	Quinohemoprotein amine dehydrogenase maturase
orf00699-480	-1.05938	8.18E-05	0.001186	down	50S ribosomal protein L6
orf05149-3514	-1.073	8.34E-05	0.001203	down	_
orf02895-1968	-1.06581	8.34E-05	0.001203	down	50S ribosomal protein L13
orf03683-2504	-1.07073	8.46E-05	0.001218	down	_
orf08158-5594	1.052543	8.96E-05	0.001287	up	_
orf01449-996	1.073458	8.99E-05	0.001288	up	Cyclopropane-fatty-acyl-phospholipid synthase
orf04255-2892	1.079537	9.15E-05	0.001308	up	_
orf05256-3601	1.052905	9.90E-05	0.001412	up	_
orf06142-4225	-1.07609	9.93E-05	0.001413	down	_
orf02325-1584	-1.05261	0.000103	0.001467	down	_
orf05008-3414	1.039059	0.000117	0.001655	up	_
orf01383-950	-1.03572	0.000122	0.001716	down	_
orf04384-2978	1.054603	0.000124	0.00175	up	_
orf01321-908	1.172322	0.000141	0.001983	up	_
orf07797-5343	-1.04896	0.000145	0.002028	down	_
orf03524-2388	-1.04035	0.000151	0.002114	down	Prephenate dehydratase
orf07801-5346	-1.02993	0.000159	0.00221	down	Dihydrodipicolinate synthase
orf00995-679	1.018216	0.000166	0.002305	up	_
orf00672-460	1.015217	0.000167	0.00231	up	_
orf08154-5591	1.073054	0.000171	0.002366	up	_
orf03832-2604	-1.01427	0.000178	0.002461	down	3-Isopropylmalate dehydratase large subunit
orf06915-4780	1.084719	0.00018	0.002478	up	Hypothetical protein T1E_3306
orf07133-4926	-1.01598	0.00018	0.002478	down	_
orf07788-5336	-1.03028	0.000181	0.002478	down	_
orf00369-250	-1.03429	0.000187	0.002547	down	Peptidyl-prolyl cis-trans isomerase

orf07488-5145	-1.02308	0.000187	0.002547	down	_
orf04585-3117	1.092887	0.000187	0.002547	up	GTP cyclohydrolase I
orf03277-2217	-1.00852	0.000192	0.002601	down	Enolase, partial
orf07790-5337	-1.011	0.000192	0.002601	down	_
orf07119-4915	1.001533	0.000194	0.00262	up	Molecular chaperone HtpG
orf06568-4530	-1.07582	0.000197	0.002653	down	_
orf00889-604	1.047236	0.000213	0.002866	up	_
orf01340-923	-1.08164	0.000217	0.002911	down	Hypothetical protein
orf04774-3257	1.077005	0.000219	0.002926	up	Amino acid ABC transporter permease
orf08103-5560	-1.00082	0.000223	0.002977	down	GMP synthase
orf03449-2336	1.002475	0.000241	0.003189	up	_
orf02608-1786	-1.0513	0.000255	0.003361	down	tRNA pseudouridine synthase B
orf07695-5280	-1.0294	0.000258	0.003398	down	_
orf02617-1792	1.066413	0.000263	0.003435	up	_
orf00665-454	1.022769	0.000263	0.003435	up	_
orf04775-3258	1.031273	0.000263	0.003435	up	_
orf05046-3440	1.017513	0.000263	0.003435	up	_
orf07034-4862	-1.02215	0.000268	0.003483	down	NADH:ubiquinone oxidoreductase subunit J
orf03618-2458	1.012758	0.000273	0.003549	up	_
orf05779-3964	-1.04455	0.000284	0.003685	down	Hypothetical protein
orf05601-3837	-1.01541	0.00029	0.003738	down	_
orf07845-5379	-1.02279	0.000305	0.003923	down	_
orf01575-1085	-1.04936	0.000311	0.003984	down	Hypothetical protein
orf01568-1079	-1.01799	0.000326	0.004131	down	Orotate phosphoribosyltransferase
orf04144-2816	1.00041	0.000342	0.004334	up	_
orf04947-3372	-1.05439	0.000401	0.004998	down	Hypothetical protein

orf04067-2760	1.028922	0.000426	0.005251	up	Uncharacterized protein ALO76_04692
orf00667-456	1.044282	0.000484	0.005884	up	Formate dehydrogenase-N subunit beta
orf03629-2466	-1.09141	0.000572	0.006811	down	_
orf04143-2815	1.018241	0.00059	0.007009	up	Hypothetical protein
orf05807-3982	1.078826	0.000636	0.007478	up	_
orf00571-391	1.027382	0.00066	0.007754	up	_
orf06659-4594	1.017765	0.000951	0.010723	up	_
orf02357-1605	-1.01672	0.001059	0.01179	down	Hypothetical protein YSA_03756
orf05271-3611	1.060291	0.00168	0.017409	up	_
orf07635-5243	1.036684	0.001977	0.020198	up	Hypothetical protein T1E_4094
orf04532-3076	1.050041	0.00211	0.021232	up	Hypothetical protein L321_02567
**In response to PHBA**					
orf06997-4838	4.754611	3.46E-50	2.08E-46	up	_
orf07010-4846	4.416138	2.05E-47	6.14E-44	up	Hypothetical protein DW66_3957
orf02867-1948	4.56599	1.11E-46	2.22E-43	up	Hypothetical protein DW66_4785
orf04372-2969	4.004917	2.91E-39	4.37E-36	up	_
orf04508-3061	3.79963	1.55E-33	1.86E-30	up	Hypothetical protein
orf06882-4763	3.584481	3.68E-33	3.68E-30	up	_
orf00711-492	-3.55791	4.80E-32	4.11E-29	down	50S ribosomal protein L2
orf00732-506	-3.50679	2.70E-31	2.02E-28	down	_
orf08123-5571	-3.49069	5.66E-31	3.77E-28	down	_
orf00708-489	-3.62064	9.00E-31	4.93E-28	down	_
orf07312-5038	3.592474	9.04E-31	4.93E-28	up	_
orf03165-2145	3.769153	2.79E-30	1.39E-27	up	_
orf00704-485	-3.57902	3.04E-30	1.40E-27	down	_

orf05140-3507	-3.78819	6.32E-30	2.71E-27	down	_
orf02260-1542	-3.22705	8.05E-29	3.22E-26	down	Membrane protein
orf00710-491	-3.53595	8.72E-29	3.27E-26	down	_
orf00713-494	-3.38217	1.33E-28	4.69E-26	down	_
orf00380-258	3.289871	1.49E-28	4.96E-26	up	_
orf00702-483	-3.34444	2.86E-28	9.01E-26	down	_
orf00729-504	-3.44308	1.37E-27	3.99E-25	down	50S ribosomal protein L10
orf07132-4925	-3.18676	1.40E-27	3.99E-25	down	Hypothetical protein AW09_001448
orf00701-482	-3.52857	1.71E-27	4.66E-25	down	30S ribosomal protein S14
orf02276-1553	3.603505	1.93E-27	5.02E-25	up	_
orf08145-5585	-3.05665	3.67E-27	9.17E-25	down	Phosphogluconate dehydratase
orf06915-4780	3.255362	5.19E-27	1.24E-24	up	Hypothetical protein T1E_3306
orf00707-488	-3.25959	2.40E-26	5.53E-24	down	_
orf07130-4924	-3.11896	2.79E-26	6.20E-24	down	_
orf00703-484	-3.43669	4.47E-26	9.58E-24	down	50S ribosomal protein L24
orf07126-4921	-3.17846	6.35E-26	1.31E-23	down	Succinate--CoA ligase subunit alpha
orf08126-5573	-3.22864	1.40E-25	2.79E-23	down	Transcriptional regulator
orf00712-493	-3.26737	2.41E-25	4.66E-23	down	_
orf07128-4922	-3.07991	3.25E-25	6.09E-23	down	_
orf08138-5580	-2.975	3.60E-25	6.54E-23	down	Sugar ABC transporter substrate-binding protein
orf08122-5570	-3.25603	4.19E-25	7.39E-23	down	_
orf05004-3411	3.678291	4.84E-25	8.29E-23	up	Hypothetical protein, conserved
orf00705-486	-3.40821	5.28E-25	8.80E-23	down	_
orf07129-4923	-3.02763	7.95E-25	1.29E-22	down	_
orf00579-396	2.952043	1.33E-24	2.10E-22	up	_
orf04750-3240	2.984627	1.98E-24	3.04E-22	up	Hypothetical protein

orf00706-487	-3.48408	3.28E-24	4.92E-22	down	50S ribosomal protein L29
orf00719-498	-2.9353	5.52E-24	8.06E-22	down	Elongation factor G
orf03529-2391	-2.86484	5.06E-23	7.22E-21	down	_
orf00714-495	-3.00381	5.43E-23	7.58E-21	down	_
orf00730-505	-3.02367	6.24E-23	8.50E-21	down	_
orf00925-628	-2.83373	1.54E-22	2.06E-20	down	_
orf00722-500	-3.07281	2.37E-22	3.09E-20	down	Hypothetical protein K151_2921
orf00718-497	-2.79981	2.54E-22	3.24E-20	down	Elongation factor Tu
orf00690-472	-2.95923	3.43E-22	4.29E-20	down	_
orf00927-630	-2.81436	4.71E-22	5.77E-20	down	Dihydrolipoamide acetyltransferase
orf08129-5575	-2.79249	5.95E-22	7.13E-20	down	_
orf08124-5572	-2.81731	1.08E-21	1.27E-19	down	_
orf00720-499	-2.91378	1.55E-21	1.78E-19	down	30S ribosomal protein S7
orf02258-1541	-3.19867	2.73E-21	3.09E-19	down	30S ribosomal protein S18
orf07823-5363	-2.84467	4.84E-21	5.38E-19	down	_
orf02668-1822	-2.77296	4.96E-21	5.40E-19	down	_
orf00686-469	-2.97972	7.08E-21	7.46E-19	down	_
orf02968-2015	-2.85883	7.09E-21	7.46E-19	down	_
orf05139-3506	-2.92695	1.69E-20	1.74E-18	down	D-glycerate dehydrogenase
orf04882-3328	2.782096	2.09E-20	2.12E-18	up	LysR family transcriptional regulator
orf00700-481	-2.69926	2.68E-20	2.68E-18	down	_
orf03132-2124	-2.84456	2.97E-20	2.92E-18	down	_
orf00716-496	-2.89847	3.58E-20	3.46E-18	down	30S ribosomal protein S10
orf03477-2357	2.617694	4.03E-20	3.83E-18	up	_
orf02257-1540	-2.85975	4.35E-20	4.08E-18	down	_
orf00709-490	-3.22347	4.81E-20	4.44E-18	down	50S ribosomal protein L22

orf08130-5576	-2.62941	1.09E-19	9.92E-18	down	ABC transporter substrate-binding protein
orf02611-1788	-2.99813	1.34E-19	1.19E-17	down	_
orf00727-503	-3.03725	1.35E-19	1.19E-17	down	_
orf00687-470	-2.69077	1.40E-19	1.22E-17	down	_
orf00693-475	-2.63341	1.76E-19	1.51E-17	down	_
orf02261-1543	-2.72989	2.28E-19	1.93E-17	down	_
orf00691-473	-2.86502	6.25E-19	5.21E-17	down	_
orf01414-969	-2.5966	7.88E-19	6.47E-17	down	ATP synthase F1 sector subunit beta
orf05135-3503	-2.65386	9.45E-19	7.65E-17	down	AP endonuclease
orf05255-3600	3.113772	1.28E-18	1.03E-16	up	_
orf00689-471	-2.64741	2.31E-18	1.82E-16	down	_
orf01409-966	-2.5635	2.35E-18	1.83E-16	down	_
orf00695-477	-2.81998	6.65E-18	5.11E-16	down	_
orf02896-1969	-2.65316	1.09E-17	8.28E-16	down	_
orf00698-479	-2.69015	1.29E-17	9.67E-16	down	_
orf03131-2123	-2.64896	1.48E-17	1.10E-15	down	_
orf00692-474	-2.98535	2.14E-17	1.56E-15	down	_
orf07522-5167	2.422298	2.36E-17	1.71E-15	up	Branched-chain alpha-keto acid dehydrogenase subunit E2
orf03242-2194	-2.56668	2.44E-17	1.74E-15	down	_
orf00645-440	-2.55931	2.80E-17	1.98E-15	down	Outer membrane protein W
orf00697-478	-2.49542	6.99E-17	4.87E-15	down	_
orf07011-4847	2.603604	7.89E-17	5.44E-15	up	Short-chain dehydrogenase
orf02969-2016	-2.33625	1.21E-16	8.21E-15	down	Molecular chaperone GroEL
orf00365-246	-2.62012	1.96E-16	1.32E-14	down	Ribosomal protein L21
orf00311-211	-2.50083	2.88E-16	1.92E-14	down	_
orf04144-2816	2.351922	3.10E-16	2.05E-14	up	_

orf07133-4926	-2.45083	4.30E-16	2.79E-14	down	_
orf07848-5381	2.277854	4.33E-16	2.79E-14	up	Hypothetical protein YSA_07493
orf01407-965	-2.34009	5.35E-16	3.41E-14	down	_
orf00699-480	-2.40022	5.53E-16	3.49E-14	down	50S ribosomal protein L6
orf03244-2196	-2.46399	7.26E-16	4.53E-14	down	Elongation factor Ts
orf01479-1015	-2.35427	8.88E-16	5.49E-14	down	Pyruvate carboxylase
orf00364-245	-2.6629	1.01E-15	6.19E-14	down	_
orf00169-117	-2.25108	2.29E-15	1.39E-13	down	Cytochrome ubiquinol oxidase subunit I
orf05136-3504	-2.2167	2.42E-15	1.45E-13	down	_
orf06284-4331	2.359767	2.70E-15	1.60E-13	up	Putative periplasmic binding protein
orf07134-4927	-2.26503	2.84E-15	1.67E-13	down	Succinate dehydrogenase flavoprotein subunit
orf06652-4589	2.269885	3.42E-15	1.98E-13	up	_
orf04566-3101	-2.29019	3.44E-15	1.98E-13	down	_
orf02606-1785	-2.20048	4.23E-15	2.42E-13	down	Translation initiation factor IF-2
orf02690-1836	2.412393	5.11E-15	2.89E-13	up	Cupin
orf05069-3456	2.583122	5.45E-15	3.05E-13	up	Hypothetical protein T1E_5212
orf00724-501	-2.20267	5.68E-15	3.15E-13	down	Uncharacterised protein
orf00736-509	-2.232	7.89E-15	4.34E-13	down	Elongation factor Tu
orf01410-967	-2.25494	1.14E-14	6.20E-13	down	_
orf00694-476	-2.3627	1.39E-14	7.49E-13	down	_
orf00018-14	-2.17806	1.91E-14	1.02E-12	down	_
orf00726-502	-2.16042	1.98E-14	1.05E-12	down	DNA-directed RNA polymerase subunit beta
orf08030-5507	-2.33247	2.18E-14	1.14E-12	down	_
orf05131-3500	-2.15342	2.21E-14	1.15E-12	down	_
orf03528-2390	-2.18808	2.24E-14	1.16E-12	down	_
orf01726-1187	2.332722	3.01E-14	1.54E-12	up	Hypothetical protein

orf03683-2504	-2.48234	4.14E-14	2.10E-12	down	_
orf03133-2125	-2.55334	4.22E-14	2.13E-12	down	_
orf02243-1531	-2.11228	6.57E-14	3.28E-12	down	_
orf04557-3094	2.287219	7.48E-14	3.71E-12	up	Hypothetical protein
orf01477-1014	-2.18011	9.38E-14	4.61E-12	down	_
23s_rRNA-4	2.03697	1.11E-13	5.39E-12	up	_
orf03790-2577	-2.24015	3.02E-13	1.46E-11	down	Acyl carrier protein
orf05074-3460	-2.24589	3.61E-13	1.73E-11	down	_
orf01908-1312	-2.2452	4.11E-13	1.95E-11	down	Ribosomal protein L31
orf01981-1357	-2.06771	4.84E-13	2.28E-11	down	Glutamine synthetase
orf05036-3431	-2.08161	1.07E-12	5.02E-11	down	_
orf04354-2956	-1.98155	2.32E-12	1.08E-10	down	Bifunctional aconitate hydratase 2/2-methylisocitrate dehydratase
orf00166-115	-2.29874	2.38E-12	1.10E-10	down	_
orf04777-3259	1.973502	3.06E-12	1.40E-10	up	Amino acid ABC transporter
orf05128-3498	-1.85044	3.54E-12	1.60E-10	down	GMC family oxidoreductase
23s_rRNA-3	1.897842	3.58E-12	1.60E-10	up	_
23s_rRNA-1	1.89784	3.58E-12	1.60E-10	up	_
23s_rRNA-2	1.893967	3.93E-12	1.74E-10	up	_
23s_rRNA-6	1.893864	3.94E-12	1.74E-10	up	_
23s_rRNA-5	1.882661	5.18E-12	2.27E-10	up	_
23s_rRNA-7	1.88155	5.32E-12	2.31E-10	up	_
orf06276-4325	1.940534	5.85E-12	2.52E-10	up	Aldehyde dehydrogenase
orf05129-3499	-1.81377	6.41E-12	2.75E-10	down	_
orf07009-4845	1.919772	7.04E-12	2.99E-10	up	Uncharacterised protein
orf07257-5001	-1.97061	7.10E-12	3.00E-10	down	_
orf07521-5166	1.939012	8.66E-12	3.63E-10	up	2-Oxoisovalerate dehydrogenase subunit beta

orf01412-968	-2.0934	1.18E-11	4.90E-10	down	_
orf04884-3329	1.919222	1.29E-11	5.35E-10	up	_
orf06277-4326	1.887175	1.53E-11	6.27E-10	up	_
orf06858-4748	-1.93545	1.67E-11	6.82E-10	down	Isocitrate dehydrogenase
orf02895-1968	-2.13134	1.75E-11	7.11E-10	down	50S ribosomal protein L13
orf02759-1883	-2.00442	1.86E-11	7.48E-10	down	_
orf03257-2205	-1.94224	2.22E-11	8.89E-10	down	_
orf07797-5343	-1.85821	2.27E-11	9.02E-10	down	_
orf05434-3729	1.926602	2.37E-11	9.37E-10	up	_
orf04286-2914	-1.98445	2.65E-11	1.04E-09	down	Trigger factor
orf01230-839	-2.27142	2.72E-11	1.06E-09	down	_
orf04094-2778	-1.95042	3.02E-11	1.17E-09	down	_
orf02760-1884	-1.9501	3.66E-11	1.41E-09	down	Membrane protein
orf01009-688	1.974344	4.21E-11	1.61E-09	up	Hypothetical protein YSA_05583
orf08301-5688	-1.969	4.53E-11	1.72E-09	down	Superoxide dismutase
orf04369-2968	1.837623	4.84E-11	1.82E-09	up	Hypothetical protein, conserved
orf08143-5584	-1.89498	4.97E-11	1.86E-09	down	_
orf06938-4796	2.067663	5.40E-11	2.01E-09	up	_
orf07525-5168	1.842344	6.04E-11	2.24E-09	up	_
orf03311-2243	-1.96667	8.13E-11	2.99E-09	down	_
orf07135-4928	-2.00848	9.39E-11	3.43E-09	down	Succinate dehydrogenase
orf02866-1947	1.813108	1.25E-10	4.55E-09	up	_
orf02725-1862	1.851994	1.31E-10	4.72E-09	up	_
orf07025-4857	-1.76595	1.50E-10	5.40E-09	down	_
orf02270-1549	-1.81762	1.52E-10	5.44E-09	down	_
orf02590-1774	-1.84205	1.91E-10	6.75E-09	down	Transcription elongation factor GreA

orf04565-3100	-2.02827	1.91E-10	6.75E-09	down	_
orf07119-4915	-1.81954	1.99E-10	6.98E-09	down	Molecular chaperone HtpG
orf07353-5065	1.930595	2.00E-10	6.98E-09	up	_
orf07541-5179	-1.83959	2.18E-10	7.52E-09	down	Aspartate kinase
orf03857-2620	-1.76823	2.18E-10	7.52E-09	down	_
orf04407-2992	1.827672	2.23E-10	7.63E-09	up	_
orf04294-2919	-1.85113	3.17E-10	1.08E-08	down	Transcriptional regulator
orf04562-3098	-1.77166	3.72E-10	1.26E-08	down	Threonine/alanine tRNA ligase second additional domain protein
orf06750-4665	1.802097	4.08E-10	1.37E-08	up	_
orf07259-5002	-1.78612	5.86E-10	1.96E-08	down	_
orf04564-3099	-1.90968	5.92E-10	1.97E-08	down	_
orf07292-5025	-1.98116	6.07E-10	2.01E-08	down	_
orf01154-788	1.73436	7.75E-10	2.55E-08	up	TIGR02444 family protein
orf02689-1835	1.697718	8.02E-10	2.63E-08	up	3-Hydroxyisobutyrate dehydrogenase
orf01143-781	1.743449	8.20E-10	2.67E-08	up	Hypothetical protein
orf08134-5578	-1.67948	9.39E-10	3.04E-08	down	_
orf03598-2442	-1.67518	1.00E-09	3.23E-08	down	_
orf01582-1090	-1.97568	1.18E-09	3.78E-08	down	50S ribosomal protein L28
orf06676-4607	-1.74538	1.24E-09	3.96E-08	down	_
orf07149-4938	-1.72919	1.31E-09	4.14E-08	down	_
orf08147-5586	-1.91088	1.37E-09	4.31E-08	down	Glyceraldehyde-3-phosphate dehydrogenase
orf07892-5410	-1.67218	1.37E-09	4.32E-08	down	Ribonucleotide-diphosphate reductase subunit alpha
orf08154-5591	1.737347	1.79E-09	5.60E-08	up	_
orf02613-1789	-1.71073	1.98E-09	6.15E-08	down	Polyribonucleotide nucleotidyltransferase
orf00781-539	1.675098	2.25E-09	6.95E-08	up	_
orf00873-593	-1.67209	2.72E-09	8.38E-08	down	Acyl-CoA dehydrogenase

orf01749-1202	1.673707	2.83E-09	8.66E-08	up	Hypothetical protein PPUTLS46_018104
orf03277-2217	-1.73048	4.15E-09	1.26E-07	down	Enolase, partial
orf08005-5486	1.65596	4.84E-09	1.46E-07	up	DEAD/DEAH box helicase
orf01930-1326	-1.61911	5.00E-09	1.51E-07	down	_
orf06660-4595	-1.68068	5.14E-09	1.54E-07	down	_
orf07983-5468	1.715424	5.33E-09	1.59E-07	up	_
orf01614-1114	1.624692	5.42E-09	1.61E-07	up	_
orf05605-3840	1.664474	6.07E-09	1.79E-07	up	_
orf01524-1046	1.649679	6.09E-09	1.79E-07	up	_
orf03158-2140	1.737484	6.14E-09	1.80E-07	up	DNA repair protein HhH-GPD
orf02021-1385	1.742846	6.38E-09	1.85E-07	up	Diguanylate phosphodiesterase
orf04161-2829	1.653506	6.38E-09	1.85E-07	up	_
orf07864-5392	1.626306	7.30E-09	2.10E-07	up	_
orf07040-4865	-1.63568	7.34E-09	2.11E-07	down	NADH:ubiquinone oxidoreductase subunit M
orf02604-1784	-1.63373	8.10E-09	2.31E-07	down	Transcription termination factor NusA
orf02608-1786	-1.42865	9.11E-09	2.59E-07	down	tRNA pseudouridine synthase B
orf07041-4866	-1.61445	1.20E-08	3.38E-07	down	_
orf02275-1552	1.600226	1.22E-08	3.45E-07	up	_
orf07315-5040	1.585597	1.30E-08	3.63E-07	up	Hypothetical protein ALO46_102909
orf03785-2573	-1.94932	1.43E-08	3.98E-07	down	50S ribosomal protein L32
orf06289-4336	1.708579	1.49E-08	4.15E-07	up	Hypothetical protein, conserved
orf03474-2355	1.60494	1.53E-08	4.22E-07	up	_
orf03519-2386	-1.54891	1.58E-08	4.34E-07	down	Hypothetical protein
orf07022-4855	-1.65981	1.62E-08	4.45E-07	down	_
orf05137-3505	-1.57865	1.71E-08	4.67E-07	down	Putative 2-ketogluconate kinase
orf03784-2572	-1.76347	1.96E-08	5.31E-07	down	_

orf01911-1314	-1.62708	1.97E-08	5.33E-07	down	_
orf00106-70	-1.74831	2.27E-08	6.11E-07	down	Nucleoside-diphosphate kinase
orf04645-3164	-1.7082	2.63E-08	7.05E-07	down	Transcriptional regulator
orf07801-5346	-1.58145	2.69E-08	7.16E-07	down	Dihydrodipicolinate synthase
orf07261-5003	-1.7172	2.79E-08	7.41E-07	down	_
orf03522-2387	-1.5628	2.97E-08	7.84E-07	down	P-protein
orf03837-2609	-1.64661	3.24E-08	8.52E-07	down	Aspartate-semialdehyde dehydrogenase
orf01367-939	1.523932	3.29E-08	8.60E-07	up	MFS transporter
orf03199-2166	-1.61691	3.30E-08	8.60E-07	down	Adenylate kinase
orf05126-3497	-1.4631	3.73E-08	9.68E-07	down	_
orf00420-289	1.665881	3.85E-08	9.94E-07	up	_
orf04347-2952	1.542906	4.56E-08	1.17E-06	up	Citrate synthase/methylcitrate synthase
orf01268-869	1.52562	4.89E-08	1.25E-06	up	GTP-binding protein
orf06708-4634	1.663196	4.90E-08	1.25E-06	up	_
orf07023-4856	-1.54584	5.06E-08	1.28E-06	down	NADH-quinone oxidoreductase subunit C/D
orf00096-64	-1.51635	5.68E-08	1.44E-06	down	GTP-binding protein
orf02753-1878	1.517321	5.85E-08	1.47E-06	up	_
orf08127-5574	-1.79061	5.85E-08	1.47E-06	down	_
orf00592-403	-1.72537	6.01E-08	1.50E-06	down	_
orf00571-391	1.612918	6.21E-08	1.54E-06	up	_
orf00165-114	-1.4767	6.56E-08	1.63E-06	down	_
orf04023-2731	1.509023	6.87E-08	1.69E-06	up	_
orf07543-5180	-1.49729	7.28E-08	1.79E-06	down	Alanine--tRNA ligase
orf00506-346	1.491538	7.45E-08	1.82E-06	up	Bcr/CflA family drug resistance efflux transporter
orf00207-138	-1.44485	9.05E-08	2.20E-06	down	PTS system fructose-specific transporter subunits IIBC
orf07989-5471	1.524025	9.62E-08	2.34E-06	up	Membrane protein

orf02882-1960	1.488254	1.02E-07	2.46E-06	up	_
orf07026-4858	-1.58258	1.05E-07	2.52E-06	down	NADH dehydrogenase I subunit F
orf03627-2464	-1.62231	1.07E-07	2.55E-06	down	Glutathione S-transferase
orf06659-4594	1.567353	1.07E-07	2.55E-06	up	_
orf01927-1325	-1.45512	1.08E-07	2.58E-06	down	_
orf06283-4330	1.662309	1.10E-07	2.62E-06	up	Amino acid ABC transporter substrate-binding protein
orf00882-599	-1.43563	1.17E-07	2.76E-06	down	Biotin synthase
orf03546-2403	-1.58432	1.30E-07	3.04E-06	down	Acetyltransferase (isoleucine patch superfamily)
orf07054-4875	1.439946	1.33E-07	3.09E-06	up	Lysine decarboxylase
orf03130-2122	-2.00576	1.33E-07	3.09E-06	down	RNA-binding protein
orf02580-1769	-1.46757	1.33E-07	3.09E-06	down	_
orf02405-1639	1.610782	1.35E-07	3.12E-06	up	_
orf05000-3407	1.424175	1.50E-07	3.45E-06	up	Hypothetical protein RK21_01722
orf08136-5579	-1.42588	1.52E-07	3.49E-06	down	_
orf01682-1159	1.440525	1.57E-07	3.60E-06	up	_
orf03524-2388	-1.44113	1.60E-07	3.65E-06	down	Prephenate dehydratase
orf00209-139	-1.49384	1.71E-07	3.86E-06	down	1-Phosphofructokinase
orf08096-5555	-1.41873	1.71E-07	3.86E-06	down	Phosphoribosylformylglycinamidine synthase, partial
orf04785-3265	-1.41765	1.73E-07	3.90E-06	down	_
orf03854-2619	-1.51119	1.78E-07	3.99E-06	down	Amidophosphoribosyltransferase
orf00214-141	-1.38612	1.84E-07	4.11E-06	down	PTS fructose transporter subunit IIA
orf01296-891	1.511024	1.92E-07	4.29E-06	up	Hypothetical protein YSA_05171
orf07281-5017	-1.64218	1.95E-07	4.33E-06	down	Coproporphyrinogen III oxidase
orf06432-4436	1.428425	1.97E-07	4.33E-06	up	_
orf00387-263	-1.44102	1.97E-07	4.33E-06	down	Energy-dependent translational throttle protein EttA
orf03599-2443	-1.61441	2.02E-07	4.43E-06	down	_

orf05737-3933	-1.73154	2.09E-07	4.58E-06	down	Saccharopine dehydrogenase
orf07282-5018	-1.61705	2.13E-07	4.64E-06	down	_
orf00776-535	-1.59996	2.30E-07	4.99E-06	down	_
orf07993-5475	1.443116	2.34E-07	5.08E-06	up	_
orf05761-3950	-1.34061	2.48E-07	5.34E-06	down	Porphobilinogen synthase
orf00233-158	1.478238	2.53E-07	5.44E-06	up	_
orf00391-266	-1.4312	2.77E-07	5.94E-06	down	Serine hydroxymethyltransferase
orf07782-5333	-1.50884	2.85E-07	6.08E-06	down	Malate:quinone oxidoreductase
orf00131-5809	1.595751	2.98E-07	6.33E-06	up	_
orf06573-4535	1.439618	2.99E-07	6.34E-06	up	_
orf04381-2976	1.51786	3.01E-07	6.36E-06	up	_
orf01551-1069	-1.59575	3.42E-07	7.20E-06	down	_
orf05435-3730	1.421416	3.49E-07	7.32E-06	up	LysR family transcriptional regulator
orf08139-5581	-1.45751	4.08E-07	8.51E-06	down	_
orf01984-1359	-1.43017	4.24E-07	8.79E-06	down	_
orf03526-2389	-1.44018	4.24E-07	8.79E-06	down	_
orf00527-362	1.511599	4.43E-07	9.16E-06	up	Hypothetical protein YSA_06171
orf04289-2916	-1.45188	4.45E-07	9.16E-06	down	_
orf01583-1091	-1.64969	4.59E-07	9.42E-06	down	_
orf02598-1780	-1.44248	4.62E-07	9.45E-06	down	_
orf00378-256	1.410507	4.66E-07	9.49E-06	up	_
orf00206-137	-1.90967	4.82E-07	9.80E-06	down	_
orf00095-63	-1.33517	5.01E-07	1.01E-05	down	Dehydrogenase
orf00820-564	-1.59127	5.69E-07	1.15E-05	down	_
orf00926-629	-1.86386	5.70E-07	1.15E-05	down	_
orf07316-5041	1.39258	6.38E-07	1.28E-05	up	_

orf04288-2915	-1.49315	7.07E-07	1.41E-05	down	_
orf07032-4861	-1.34362	7.38E-07	1.47E-05	down	_
orf07519-5165	1.385437	7.70E-07	1.53E-05	up	_
orf07822-5362	-1.41553	8.12E-07	1.61E-05	down	_
orf07037-4864	-1.42971	8.27E-07	1.63E-05	down	_
orf06302-4347	1.3394	8.63E-07	1.70E-05	up	Putative two-component sensor
orf01702-1171	-1.33882	8.75E-07	1.71E-05	down	_
orf05742-3936	1.393463	9.26E-07	1.80E-05	up	_
orf01062-725	1.369157	9.27E-07	1.80E-05	up	_
orf01325-911	1.354164	1.13E-06	2.20E-05	up	LysR family transcriptional regulator
orf06892-4768	1.361985	1.16E-06	2.24E-05	up	Dihydropyrimidine dehydrogenase subunit B
orf05071-3458	1.431517	1.16E-06	2.24E-05	up	Hypothetical protein
orf08024-5501	-1.3752	1.18E-06	2.28E-05	down	_
orf05710-3914	1.326927	1.29E-06	2.47E-05	up	_
orf03301-2234	1.374824	1.29E-06	2.47E-05	up	_
orf02326-1585	-1.34988	1.31E-06	2.49E-05	down	_
orf01416-970	-1.69452	1.34E-06	2.54E-05	down	_
orf07799-5345	-1.44155	1.46E-06	2.77E-05	down	Dihydrodipicolinate synthase
orf00114-75	-1.40848	1.52E-06	2.86E-05	down	(Fe-S)-cluster assembly protein
orf06766-4681	-1.54124	1.52E-06	2.86E-05	down	Sterol-binding domain protein
orf05003-3410	1.316797	1.70E-06	3.18E-05	up	Hypothetical protein YSA_11298
orf00275-187	-1.37487	1.70E-06	3.18E-05	down	_
orf01158-792	1.317677	1.72E-06	3.20E-05	up	_
orf07036-4863	-1.55807	1.79E-06	3.32E-05	down	NADH-quinone oxidoreductase subunit K
orf01687-1162	-1.40558	1.83E-06	3.39E-05	down	Thioredoxin
orf01621-1119	1.273352	1.98E-06	3.66E-05	up	Mechanosensitive ion channel protein MscS

orf05772-3959	-1.36335	2.00E-06	3.69E-05	down	_
orf03887-2639	1.410632	2.17E-06	3.97E-05	up	_
orf02939-1996	-1.36021	2.21E-06	4.03E-05	down	_
orf05048-3441	1.339899	2.29E-06	4.17E-05	up	Hypothetical protein
orf01383-950	-1.31071	2.38E-06	4.32E-05	down	_
orf05459-3745	1.317499	2.51E-06	4.54E-05	up	_
orf03435-2328	1.307238	2.57E-06	4.64E-05	up	Diguanylate phosphodiesterase
orf08211-5628	-1.23127	2.58E-06	4.64E-05	down	_
orf03476-2356	1.437326	2.59E-06	4.66E-05	up	_
orf01738-1196	1.300207	2.61E-06	4.66E-05	up	Putative adenosylmethionine-8-amino-7-oxononanoate aminotransferase
orf02876-1955	-1.26562	2.65E-06	4.72E-05	down	_
orf07566-5196	1.284111	2.66E-06	4.73E-05	up	Acetyl-coenzyme A synthetase 1
orf03859-2621	-1.20103	2.74E-06	4.86E-05	down	Oxidoreductase
orf06995-4837	1.308417	2.81E-06	4.97E-05	up	_
orf05133-3501	-1.33765	2.85E-06	5.03E-05	down	Transcriptional regulator
orf00824-566	-1.31506	2.94E-06	5.18E-05	down	RNA polymerase sigma factor RpoD
orf04524-3071	1.320022	3.08E-06	5.40E-05	up	Hypothetical protein PPS11_31076
orf05419-3719	-1.30002	3.16E-06	5.53E-05	down	_
orf01619-1118	1.283276	3.26E-06	5.68E-05	up	FAD dependent oxidoreductase
orf03862-2623	1.287942	3.28E-06	5.70E-05	up	AraC family transcriptional regulator
orf04102-2784	1.282645	3.36E-06	5.82E-05	up	_
orf03248-2198	-1.47042	3.73E-06	6.44E-05	down	Ribosome recycling factor
orf03700-2515	-1.27851	3.91E-06	6.73E-05	down	_
orf06857-4747	1.273919	3.97E-06	6.81E-05	up	Isocitrate dehydrogenase
orf02470-1692	1.19068	4.02E-06	6.89E-05	up	_
orf07151-4939	-1.35748	4.09E-06	6.98E-05	down	_

orf04095-2779	-1.23498	4.26E-06	7.26E-05	down	Glyceraldehyde-3-phosphate dehydrogenase
orf02123-1453	-1.35236	4.42E-06	7.51E-05	down	_
orf02235-1526	-1.44745	4.46E-06	7.55E-05	down	RNA-binding protein Hfq
orf03427-2323	1.252527	4.56E-06	7.70E-05	up	Hypothetical protein
orf03256-2204	-1.22943	5.09E-06	8.57E-05	down	Membrane protein
orf04604-3134	1.273768	5.22E-06	8.76E-05	up	_
16s_rRNA-18	-1.52095	5.53E-06	9.26E-05	down	_
orf01437-985	1.249344	5.59E-06	9.34E-05	up	_
orf04323-2939	-1.42798	5.67E-06	9.44E-05	down	Phospho-2-dehydro-3-deoxyheptonate aldolase
orf03141-2130	-1.23556	5.70E-06	9.46E-05	down	Homoserine dehydrogenase
orf01613-1113	1.247296	5.74E-06	9.50E-05	up	LysR family transcriptional regulator
orf05072-3459	-1.44559	7.12E-06	0.000117	down	_
orf02688-1834	1.21744	7.13E-06	0.000117	up	_
orf00961-653	-1.3201	7.40E-06	0.000121	down	_
orf00116-76	-1.46522	7.62E-06	0.000125	down	_
orf07836-5372	-1.19651	7.64E-06	0.000125	down	_
orf06281-4329	1.310078	7.79E-06	0.000127	up	_
orf03273-2215	-1.22086	8.00E-06	0.00013	down	CTP synthetase
orf00210-140	-1.07547	8.02E-06	0.00013	down	1-Phosphofructokinase
orf07262-5004	-1.28665	8.37E-06	0.000135	down	Cytochrome CBB3
orf00363-244	-1.24698	8.49E-06	0.000137	down	_
orf00167-116	-1.61328	8.54E-06	0.000137	down	Cytochrome o ubiquinol oxidase
orf05078-3464	1.204303	8.57E-06	0.000137	up	_
orf03305-2237	1.267639	8.63E-06	0.000138	up	Hypothetical protein
orf02549-1747	-1.21429	8.87E-06	0.000141	down	_
orf04069-2761	-1.24172	9.87E-06	0.000157	down	Fatty acid oxidation complex subunit alpha

orf03547-2404	-1.21787	1.07E-05	0.000169	down	_
orf00626-425	-1.31008	1.12E-05	0.000176	down	_
orf05607-3841	1.240556	1.12E-05	0.000177	up	Hypothetical protein PP4_24180
orf03982-2704	-1.22261	1.15E-05	0.000181	down	Phosphoenolpyruvate synthase
orf00091-61	-1.2254	1.16E-05	0.000181	down	Aminotransferase
orf03983-2705	1.196913	1.16E-05	0.000182	up	Hypothetical protein, conserved
orf02853-1939	1.186876	1.21E-05	0.000189	up	ATPase
orf01554-1071	-1.2069	1.26E-05	0.000197	down	_
orf03206-2171	1.207832	1.29E-05	0.0002	up	Extensin
orf02550-1748	-1.32326	1.40E-05	0.000217	down	_
orf03352-2267	-1.12266	1.44E-05	0.000223	down	Phosphoribosylaminoimidazole synthetase
orf04587-3119	1.190359	1.46E-05	0.000225	up	_
orf01320-907	1.183163	1.49E-05	0.000229	up	_
orf02358-1606	-1.18744	1.54E-05	0.000237	down	Phosphoribosylamine--glycine ligase
orf02392-1631	-1.18818	1.58E-05	0.000241	down	_
orf07137-4929	-1.29022	1.59E-05	0.000242	down	_
orf08187-5616	-1.18478	1.61E-05	0.000244	down	Succinate dehydrogenase, hydrophobic membrane anchor protein
orf04160-2828	-1.29238	1.67E-05	0.000253	down	_
orf02938-1995	1.160985	1.68E-05	0.000253	up	_
orf03406-2307	-1.84679	1.86E-05	0.00028	down	_
orf00098-65	-1.22653	1.87E-05	0.000281	down	Methyl-accepting chemotaxis sensory transducer
orf00049-5739	-1.09377	1.88E-05	0.000282	down	Histidine--tRNA ligase
orf06889-4766	1.180866	1.90E-05	0.000283	up	_
orf07564-5195	1.494197	1.94E-05	0.00029	up	_
orf00248-170	1.177849	2.01E-05	0.000299	up	_
orf07845-5379	-1.12302	2.03E-05	0.000301	down	_

orf02325-1584	-1.17322	2.13E-05	0.000315	down	_
orf00482-331	-1.16187	2.15E-05	0.000318	down	_
orf04320-2937	-1.17285	2.21E-05	0.000326	down	_
orf02937-1994	-1.1826	2.29E-05	0.000336	down	Hypothetical protein
orf06528-4504	1.165483	2.30E-05	0.000338	up	_
orf03995-2713	-1.14978	2.55E-05	0.000373	down	_
orf01207-823	1.158136	2.62E-05	0.000382	up	_
orf07112-4910	-1.19478	2.64E-05	0.000384	down	_
orf03600-2444	-1.06888	2.78E-05	0.000403	down	_
orf01142-780	1.178857	2.83E-05	0.000409	up	_
orf02642-1805	-1.23069	2.90E-05	0.000418	down	Histidine kinase
orf04310-2930	-1.24686	2.90E-05	0.000418	down	_
orf02119-1450	-1.15229	2.94E-05	0.000422	down	_
orf03144-2131	-1.20966	2.94E-05	0.000422	down	Phosphoglycerate kinase
orf02898-1971	-1.15555	3.10E-05	0.000444	down	Threonine synthase, partial
orf03834-2606	-1.22914	3.18E-05	0.000454	down	Unnamed protein product
orf05467-3753	1.193067	3.23E-05	0.000459	up	_
orf07034-4862	-1.13635	3.32E-05	0.000471	down	3-Oxoacid CoA-transferase subunit B
orf03816-2594	-1.16595	3.43E-05	0.000486	down	NADH:ubiquinone oxidoreductase subunit J
orf01787-1227	1.153334	3.52E-05	0.000498	up	_
orf05376-3689	-1.07907	3.72E-05	0.000525	down	Methyl-accepting chemotaxis sensory transducer
orf02400-1636	-1.0978	3.78E-05	0.000532	down	_
orf03246-2197	-1.19843	4.04E-05	0.000567	down	_
orf03482-2361	1.112308	4.05E-05	0.000567	up	_
orf00361-243	-1.08629	4.05E-05	0.000567	down	Hypothetical protein
orf04927-3360	-1.08935	4.07E-05	0.000567	down	_

orf00487-334	-1.39466	4.19E-05	0.000583	down	Cobalamin biosynthesis protein CobW
orf03810-2589	-1.17117	4.25E-05	0.00059	down	_
orf07479-5140	-1.1823	4.30E-05	0.000595	down	_
orf00750-518	-1.10561	4.33E-05	0.000597	down	_
orf03791-2578	-1.11101	4.42E-05	0.000609	down	_
orf01157-791	1.111093	4.65E-05	0.000639	up	_
orf00733-507	-1.20833	4.74E-05	0.00065	down	Peptidyl-prolyl cis-trans isomerase
orf01050-716	-1.19297	4.78E-05	0.000654	down	Transcription termination/antitermination factor NusG
orf03181-2155	-1.04942	5.10E-05	0.000696	down	Porin
orf03235-2189	-1.22957	5.17E-05	0.000705	down	_
orf07838-5373	-1.12796	5.46E-05	0.000742	down	2,3,4,5-Tetrahydropyridine-2,6-dicarboxylate N-succinyltransferase
orf05775-3961	-1.29727	5.49E-05	0.000744	down	Aspartate--tRNA ligase
orf00962-654	-1.22418	5.54E-05	0.00075	down	_
orf03575-2424	-1.04865	5.57E-05	0.000753	down	_
orf02116-1448	-1.10224	5.60E-05	0.000754	down	_
orf03118-2115	-1.13889	5.96E-05	0.000801	down	_
orf04401-2988	-1.19939	6.27E-05	0.000841	down	Phosphoribosylglycinamide formyltransferase 2
orf07390-5087	-1.136	6.84E-05	0.000915	down	Sulfite reductase
orf03832-2604	-1.12683	7.05E-05	0.000941	down	_
orf01372-942	1.081687	7.06E-05	0.000941	up	3-Isopropylmalate dehydratase large subunit
orf07488-5145	-1.16314	7.24E-05	0.000962	down	LysR family transcriptional regulator
orf01550-1068	-1.2589	7.33E-05	0.000972	down	_
orf08133-5577	-1.14748	7.85E-05	0.001038	down	_
orf08103-5560	-1.20892	8.08E-05	0.001067	down	Hypothetical protein RHECNPAF_1360097
orf06288-4335	1.018545	8.24E-05	0.001085	up	GMP synthase
orf06841-4735	-1.13905	8.98E-05	0.001181	down	Hypothetical protein PPUTLS46_000315

orf08273-5667	-1.17038	9.47E-05	0.001241	down	Serine--tRNA ligase
orf00671-459	-1.12293	9.48E-05	0.001241	down	_
orf07824-5364	-1.14569	0.000103	0.00134	down	_
orf02875-1954	-1.17643	0.000107	0.001388	down	_
orf04482-3044	1.163716	0.000107	0.001388	up	Sulfate adenylate transferase, subunit 2
orf03554-2410	-1.0807	0.00011	0.001427	down	_
orf00123-82	1.045729	0.000116	0.001493	up	_
orf05982-4103	1.043285	0.000118	0.001528	up	Integrase/recombinase
orf00552-379	-1.10574	0.000119	0.001537	down	Inositol-1-monophosphatase
orf03259-2206	-1.14886	0.000121	0.001558	down	_
orf06331-4366	-1.15739	0.000126	0.001607	down	_
orf05601-3837	-1.16076	0.000128	0.00163	down	UDP-3-O-(3-hydroxymyristoyl)glucosamine N-acyltransferase
orf01450-997	1.02346	0.00013	0.00165	up	_
orf03134-2126	1.026229	0.000134	0.001706	up	_
orf08106-5561	-1.15224	0.000137	0.001741	down	_
orf07418-5103	-1.04536	0.00014	0.001767	down	_
orf00273-186	-1.02849	0.000142	0.001787	down	Inosine-5'-monophosphate dehydrogenase
orf06839-4734	-1.06932	0.000144	0.001806	down	Chemotaxis protein CheA
orf02114-1446	-1.05354	0.000153	0.001911	down	Malate:quinone oxidoreductase
orf00810-558	1.037606	0.000154	0.001924	up	Siroheme synthase
orf00809-557	1.032292	0.00016	0.001995	up	S-adenosylmethionine synthetase
orf04304-2926	-1.09226	0.000163	0.002029	down	_
orf04383-2977	1.100553	0.000164	0.002035	up	Hypothetical protein
orf03513-2382	1.026248	0.000165	0.002042	up	Chemotaxis protein
orf03319-2249	-1.073	0.000166	0.002054	down	_
orf06992-4835	1.055288	0.000167	0.002058	up	_

orf05672-3886	1.090046	0.000168	0.002062	up	NAD(P)H quinone oxidoreductase
orf04109-2788	-1.85327	0.000168	0.002062	down	_
orf02403-1637	-1.03174	0.000168	0.002062	down	Short-chain dehydrogenase
orf00170-118	-1.13123	0.00017	0.002073	down	_
orf01572-1083	-1.07512	0.00017	0.002073	down	Hypothetical protein AW08_03575
orf02972-2017	-1.12303	0.000174	0.002117	down	Cytochrome ubiquinol oxidase subunit II
orf03080-2092	-1.02341	0.000175	0.002122	down	_
orf03881-2636	-1.13903	0.000179	0.00217	down	Pyruvate kinase
orf07842-5376	-1.08361	0.00018	0.002176	down	Elongation factor 4
orf07705-5288	-1.09284	0.00018	0.002176	down	Hypothetical protein PAMH27_1911
orf00127-85	-1.00332	0.000181	0.002186	down	_
orf07849-5382	-1.0338	0.000183	0.002201	down	_
orf00627-426	-1.03954	0.000185	0.002222	down	Preprotein translocase subunit SecF
orf00234-5889	1.027616	0.000189	0.002269	up	_
orf06280-4328	1.078415	0.00019	0.00227	up	_
orf03597-2441	-1.18175	0.000194	0.002321	down	_
orf07028-4859	-1.06423	0.000199	0.002374	down	_
orf06885-4764	1.054758	0.000205	0.00243	up	_
orf04920-3356	-1.09331	0.000206	0.00244	down	NADH-quinone oxidoreductase
orf03548-2405	-1.16092	0.000206	0.00244	down	Zn-dependent hydrolase
orf02944-2000	-1.04527	0.000207	0.002444	down	_
orf00112-74	-1.08228	0.000209	0.002455	down	_
orf04071-2762	-1.04488	0.000209	0.002455	down	Protein translocase subunit SecA
orf01168-798	-1.00643	0.000221	0.002588	down	_
orf01004-685	-1.10932	0.000221	0.002589	down	_
orf01386-952	-1.09513	0.000225	0.002625	down	_

orf07603-5220	-1.04528	0.000227	0.002642	down	Imidazole glycerol phosphate synthase subunit HisH
orf06866-4752	-1.09987	0.000239	0.002779	down	_
orf00624-424	-1.05025	0.000249	0.002873	down	Spy-related protein
orf07153-4940	-1.05391	0.000251	0.002892	down	_
orf00090-60	-1.10204	0.000255	0.002928	down	N utilization substance protein B homolog
orf02588-1773	-1.00457	0.000259	0.002971	down	Electron transfer flavoprotein-ubiquinone oxidoreductase
orf01006-686	-1.069	0.000284	0.003234	down	_
orf01401-961	-1.01893	0.000292	0.003315	down	Uncharacterised protein
orf03451-2337	1.010414	0.000299	0.003382	up	Imidazoleglycerol-phosphate dehydratase
orf03836-2608	-1.09702	0.000302	0.003408	down	_
orf07420-5104	-1.09333	0.000322	0.003611	down	_
orf04878-3325	1.007504	0.000323	0.003617	up	4-Hydroxybenzoate 3-monooxygenase
orf00479-329	-1.07661	0.000357	0.003937	down	Protein phosphatase CheZ
orf01688-1163	-1.02629	0.00036	0.003957	down	4-Hydroxybenzoate 3-monooxygenase
orf04284-2912	-1.0377	0.000403	0.004389	down	_
orf02578-1768	-1.13471	0.00041	0.00445	down	_
orf01423-974	-1.00251	0.000458	0.004901	down	_
orf05736-3932	-1.1888	0.000475	0.005068	down	_
orf05767-3955	1.004638	0.000477	0.00507	up	_
orf02957-2008	-1.02363	0.000489	0.00519	down	_
orf02379-1622	-1.07106	0.000493	0.005223	down	_
orf02043-1399	-1.0064	0.000524	0.005542	down	Mechanosensitive ion channel protein MscS
orf07457-5127	-1.01551	0.000529	0.005586	down	_
orf08218-5631	-1.02006	0.000561	0.005841	down	_
orf02033-1392	-1.03019	0.00059	0.006114	down	PPE family protein
orf07877-5401	-1.14722	0.000644	0.006619	down	UDP-N-acetylglucosamine 1-carboxyvinyltransferase

orf04861-3313	-1.15376	0.000781	0.007795	down	_
orf00376-255	1.082077	0.000831	0.008232	up	_
orf04066-2759	-1.1002	0.000929	0.00912	down	_
orf03629-2466	-1.56008	0.000992	0.00965	down	Hypothetical protein YSA_06429
orf07895-5412	-1.04473	0.001095	0.010418	down	_
orf07421-5105	-1.15637	0.001112	0.010536	down	_
orf01969-1349	-1.0553	0.001426	0.01311	down	Ribonucleotide-diphosphate reductase subunit beta
orf07460-5129	-1.07385	0.001502	0.013685	down	_
orf05134-3502	-1.00662	0.001695	0.015254	down	_
orf04862-3314	-1.10275	0.001966	0.017256	down	_
orf04117-2794	-1.01427	0.002148	0.018635	down	_
orf03578-2426	-1.24152	0.003444	0.027975	down	Hybrid sensor histidine kinase/response regulator
orf02593-1776	-1.17257	0.006338	0.04701	down	Chromosome segregation ATPase
**In response to FA**					
orf05164-3526	5.31914	1.45E-61	8.71E-58	up	Enoyl-CoA hydratase/aldolase
orf06515-4494	4.552923	3.25E-48	9.75E-45	up	GGDEF domain/EAL domain protein
orf05167-3527	4.364719	5.36E-47	1.07E-43	up	Vanillin dehydrogenase
orf06284-4331	4.248393	6.38E-43	9.57E-40	up	Putative periplasmic binding protein
orf05140-3507	-2.06333	2.58E-41	3.10E-38	down	_
orf06277-4326	3.974344	1.63E-40	1.63E-37	up	_
orf06510-4491	3.93725	2.76E-40	2.36E-37	up	Vanillate O-demethylase subunit
orf06511-4492	3.970246	8.58E-39	6.43E-36	up	Vanillate O-demethylase subunit
orf06276-4325	3.797502	2.76E-37	1.84E-34	up	Aldehyde dehydrogenase
orf08123-5571	-3.64597	6.99E-35	4.19E-32	down	_
orf06514-4493	3.631308	4.24E-34	2.31E-31	up	Vanillate O-demethylase subunit

orf06285-4332	3.506045	2.52E-33	1.26E-30	up	_
orf06286-4333	3.493846	9.23E-33	4.26E-30	up	Quinoprotein ethanol dehydrogenase PedE
orf06283-4330	3.681121	1.63E-32	6.99E-30	up	Amino acid ABC transporter substrate-binding protein
orf05181-3537	3.459803	1.03E-31	4.13E-29	up	Diguanylate cyclase
orf08122-5570	-3.39482	5.06E-31	1.90E-28	down	_
orf08126-5573	-3.33089	2.46E-30	8.66E-28	down	Transcriptional regulator
orf06518-4496	3.554643	1.41E-29	4.68E-27	up	Hypothetical protein RK21_01659
orf05168-3528	3.213286	3.05E-29	9.63E-27	up	Feruloyl-CoA-synthetase
orf06281-4329	3.254607	1.50E-28	4.50E-26	up	_
orf08138-5580	-3.11408	1.70E-27	4.85E-25	down	Sugar ABC transporter substrate-binding protein
orf08124-5572	-3.12153	2.02E-27	5.51E-25	down	_
orf08129-5575	-3.06812	8.03E-27	2.09E-24	down	_
orf01381-949	-3.23529	1.07E-25	2.66E-23	down	Hypothetical protein YSA_05068
orf08145-5585	-2.96544	2.79E-25	6.70E-23	down	Phosphogluconate dehydratase
orf08130-5576	-2.89875	1.16E-24	2.68E-22	down	ABC transporter substrate-binding protein
orf07009-4845	2.874908	1.09E-23	2.43E-21	up	Uncharacterised protein
orf06306-4349	2.81847	1.70E-23	3.65E-21	up	_
orf06280-4328	2.781489	2.35E-22	4.86E-20	up	_
orf05139-3506	-2.76048	2.86E-22	5.72E-20	down	D-glycerate dehydrogenase
orf04482-3044	2.767253	6.43E-21	1.24E-18	up	_
orf00842-577	-2.62481	1.29E-20	2.41E-18	down	_
orf06517-4495	2.83603	2.39E-20	4.35E-18	up	_
orf07010-4846	2.521381	3.19E-19	5.63E-17	up	Hypothetical protein DW66_3957
orf07989-5471	2.519615	1.43E-18	2.44E-16	up	Membrane protein
orf05135-3503	-2.49129	1.82E-18	3.03E-16	down	AP endonuclease
orf05174-3532	2.395215	6.50E-18	1.05E-15	up	Protein of unknown function

orf05177-3534	2.376489	7.62E-18	1.20E-15	up	_
orf07566-5196	2.408451	8.30E-18	1.28E-15	up	Acetyl-coenzyme A synthetase 1
orf03477-2357	2.397755	1.54E-17	2.31E-15	up	_
orf07128-4922	-2.32355	8.36E-17	1.22E-14	down	_
orf07126-4921	-2.30671	1.97E-16	2.81E-14	down	Succinate--CoA ligase subunit alpha
orf03827-2601	2.341902	3.47E-16	4.84E-14	up	_
orf02759-1883	-2.28026	5.11E-16	6.96E-14	down	_
orf05178-3535	2.235964	8.00E-16	1.07E-13	up	_
orf00927-630	-2.22694	1.55E-15	2.03E-13	down	Dihydrolipoamide acetyltransferase
orf05170-3529	2.180483	3.96E-15	5.05E-13	up	Acetyl-CoA acetyltransferase
orf00729-504	-2.20505	4.30E-15	5.37E-13	down	50S ribosomal protein L10
orf06304-4348	2.130931	9.92E-15	1.21E-12	up	_
orf03474-2355	2.157199	1.22E-14	1.46E-12	up	_
orf06275-4324	2.280036	1.70E-14	1.99E-12	up	_
orf00925-628	-2.11758	1.95E-14	2.25E-12	down	_
orf08143-5584	-2.11224	3.17E-14	3.59E-12	down	_
orf08127-5574	-2.1143	4.91E-14	5.45E-12	down	_
orf05131-3500	-2.06326	5.17E-14	5.64E-12	down	_
orf07130-4924	-2.08785	5.56E-14	5.96E-12	down	_
orf00926-629	-2.12831	8.41E-14	8.85E-12	down	_
orf07129-4923	-2.04617	1.50E-13	1.55E-11	down	_
orf08147-5586	-2.05736	2.02E-13	2.05E-11	down	Glyceraldehyde-3-phosphate dehydrogenase
orf00378-256	2.056093	2.31E-13	2.31E-11	up	_
orf05136-3504	-2.0132	2.61E-13	2.55E-11	down	_
orf05036-3431	-2.02051	2.64E-13	2.55E-11	down	_
orf05074-3460	-2.05076	2.82E-13	2.69E-11	down	_

orf06882-4763	2.050464	2.98E-13	2.79E-11	up	_
orf04546-3087	2.021924	3.20E-13	2.95E-11	up	Monosaccharide-transporting ATPase
orf06523-4500	1.939442	7.94E-13	7.21E-11	up	MFS transporter
orf01009-688	2.1077	8.07E-13	7.22E-11	up	Hypothetical protein YSA_05583
orf02760-1884	-1.97444	1.03E-12	9.04E-11	down	Membrane protein
orf00703-484	-2.00202	1.33E-12	1.15E-10	down	50S ribosomal protein L24
orf00702-483	-1.95672	1.53E-12	1.31E-10	down	_
orf00704-485	-1.9777	1.61E-12	1.36E-10	down	_
orf04381-2976	2.030743	1.73E-12	1.44E-10	up	_
orf00732-506	-1.95077	2.31E-12	1.90E-10	down	_
orf06179-4252	1.916152	2.97E-12	2.40E-10	up	Uncharacterised protein
orf00727-503	-1.97955	3.22E-12	2.58E-10	down	_
orf06519-4497	1.879884	3.47E-12	2.74E-10	up	_
orf07132-4925	-1.90494	3.79E-12	2.95E-10	down	Hypothetical protein AW09_001448
orf00843-578	-1.93817	4.33E-12	3.33E-10	down	Carbon-nitrogen hydrolase
orf05173-3531	1.924496	5.44E-12	4.13E-10	up	_
orf00730-505	-1.90025	6.91E-12	5.18E-10	down	_
orf00701-482	-1.94955	7.68E-12	5.68E-10	down	30S ribosomal protein S14
orf03165-2145	2.084754	9.68E-12	7.08E-10	up	_
orf00206-137	-2.11958	1.03E-11	7.42E-10	down	_
orf00705-486	-1.92723	1.15E-11	8.20E-10	down	_
orf07782-5333	-1.85727	1.25E-11	8.83E-10	down	Malate:quinone oxidoreductase
orf00706-487	-1.91912	1.28E-11	8.90E-10	down	50S ribosomal protein L29
orf00707-488	-1.88652	1.93E-11	1.33E-09	down	_
orf01479-1015	-1.83556	2.32E-11	1.58E-09	down	Pyruvate carboxylase
orf06296-4342	1.847839	2.50E-11	1.69E-09	up	Hypothetical protein PPS11_11936

orf00708-489	-1.84019	3.34E-11	2.22E-09	down	_
orf07823-5363	-1.84119	3.98E-11	2.62E-09	down	_
orf00710-491	-1.85235	5.78E-11	3.77E-09	down	_
orf05128-3498	-1.76281	8.08E-11	5.21E-09	down	GMC family oxidoreductase
orf00711-492	-1.76461	1.24E-10	7.88E-09	down	50S ribosomal protein L2
orf08134-5578	-1.83856	2.20E-10	1.38E-08	down	_
orf00709-490	-1.81046	2.22E-10	1.38E-08	down	50S ribosomal protein L22
orf02704-1846	1.740793	2.61E-10	1.61E-08	up	protocatechuate 3,4-dioxygenase subunit beta
orf02972-2017	-1.73766	2.68E-10	1.63E-08	down	Pyruvate kinase
orf08139-5581	-1.71235	2.71E-10	1.63E-08	down	_
orf00311-211	-1.75734	2.73E-10	1.63E-08	down	_
orf07011-4847	1.80627	3.05E-10	1.81E-08	up	Short-chain dehydrogenase
orf02260-1542	-1.72926	3.17E-10	1.87E-08	down	Membrane protein
orf06287-4334	1.725447	3.67E-10	2.14E-08	up	Hypothetical protein
orf00718-497	-1.69685	4.40E-10	2.54E-08	down	Elongation factor Tu
orf05172-3530	1.691532	5.04E-10	2.88E-08	up	_
orf00209-139	-1.70305	6.26E-10	3.54E-08	down	1-Phosphofructokinase
orf06302-4347	1.67348	7.19E-10	4.03E-08	up	Putative two-component sensor
orf02261-1543	-1.71767	7.26E-10	4.03E-08	down	_
orf04557-3094	1.790568	8.62E-10	4.74E-08	up	Hypothetical protein
orf01598-1103	-1.67758	9.19E-10	5.01E-08	down	Uncharacterised protein
orf00018-14	-1.68447	9.35E-10	5.05E-08	down	_
orf02258-1541	-1.75963	1.10E-09	5.89E-08	down	30S ribosomal protein S18
orf00713-494	-1.66984	1.13E-09	6.01E-08	down	_
orf02405-1639	1.914108	1.17E-09	6.13E-08	up	_
orf05129-3499	-1.64558	1.20E-09	6.23E-08	down	_

orf04383-2977	1.719294	1.29E-09	6.64E-08	up	_
orf01477-1014	-1.66336	1.30E-09	6.64E-08	down	_
orf00719-498	-1.63367	1.82E-09	9.24E-08	down	Elongation factor G
orf06857-4747	1.659878	1.84E-09	9.29E-08	up	Isocitrate dehydrogenase
orf02257-1540	-1.65296	1.88E-09	9.38E-08	down	_
orf01230-839	-1.77723	1.90E-09	9.40E-08	down	_
orf00365-246	-1.67732	2.52E-09	1.24E-07	down	Ribosomal protein L21
orf04750-3240	1.664385	2.55E-09	1.24E-07	up	Hypothetical protein
orf00712-493	-1.67092	3.38E-09	1.63E-07	down	_
orf06307-4350	1.63744	3.71E-09	1.78E-07	up	_
orf08136-5579	-1.58717	4.40E-09	2.09E-07	down	_
orf06858-4748	-1.59826	4.97E-09	2.35E-07	down	Isocitrate dehydrogenase
orf04548-3088	1.610185	5.61E-09	2.63E-07	up	ABC transporter related protein
orf00714-495	-1.58802	6.16E-09	2.86E-07	down	_
orf04355-2957	-1.60306	6.48E-09	2.99E-07	down	_
orf03476-2356	1.696261	6.86E-09	3.14E-07	up	_
orf00720-499	-1.58848	7.15E-09	3.25E-07	down	30S ribosomal protein S7
orf02705-1847	1.623189	7.84E-09	3.53E-07	up	Protocatechuate 3,4-dioxygenase subunit alpha
orf04407-2992	1.594194	1.19E-08	5.34E-07	up	_
orf06301-4346	1.607563	1.38E-08	6.14E-07	up	_
orf06708-4634	1.721367	1.77E-08	7.80E-07	up	_
orf03132-2124	-1.55029	1.85E-08	8.11E-07	down	_
orf06292-4338	1.50893	3.08E-08	1.34E-06	up	_
orf05126-3497	-1.4714	3.93E-08	1.70E-06	down	_
orf04508-3061	1.592762	3.96E-08	1.70E-06	up	Hypothetical protein
orf05137-3505	-1.4882	4.02E-08	1.71E-06	down	Putative 2-ketogluconate kinase

orf06297-4343	1.499175	4.93E-08	2.08E-06	up	_
orf06289-4336	1.675525	5.59E-08	2.34E-06	up	Hypothetical protein, conserved
orf00686-469	-1.55202	6.30E-08	2.62E-06	down	_
orf00214-141	-1.45466	6.74E-08	2.79E-06	down	PTS fructose transporter subunit IIA
orf07315-5040	1.478592	6.99E-08	2.87E-06	up	Hypothetical protein ALO46_102909
orf04369-2968	1.471666	7.41E-08	3.02E-06	up	Hypothetical protein, conserved
orf05072-3459	-1.47355	7.92E-08	3.21E-06	down	_
orf00207-138	-1.45452	8.32E-08	3.35E-06	down	PTS system fructose-specific transporter subunits IIBC
orf04378-2974	1.522519	9.99E-08	3.99E-06	up	_
orf06456-4455	1.454221	1.05E-07	4.19E-06	up	Hydroperoxidase, partial
orf05037-3432	-1.67427	1.07E-07	4.20E-06	down	Hypothetical protein, conserved
orf00687-470	-1.43708	1.21E-07	4.75E-06	down	_
orf07794-5340	-1.51618	1.30E-07	5.05E-06	down	_
orf02993-2033	1.423068	1.51E-07	5.84E-06	up	Beta-ketoadipyl CoA thiolase
orf00691-473	-1.443	1.72E-07	6.61E-06	down	_
orf04372-2969	1.450587	1.81E-07	6.92E-06	up	_
orf00690-472	-1.42783	1.93E-07	7.31E-06	down	_
orf00693-475	-1.39729	2.54E-07	9.57E-06	down	_
orf03578-2426	-1.47276	2.65E-07	9.93E-06	down	_
orf08133-5577	-1.3949	2.70E-07	1.01E-05	down	Hypothetical protein RHECNPAF_1360097
orf03242-2194	-1.40234	2.81E-07	1.04E-05	down	_
orf02580-1769	1.376051	3.30E-07	1.22E-05	up	_
orf02615-1790	1.466919	3.41E-07	1.25E-05	up	_
orf00716-496	-1.41192	4.13E-07	1.50E-05	down	30S ribosomal protein S10
orf04777-3259	-1.39442	4.36E-07	1.57E-05	down	Amino acid ABC transporter
orf03133-2125	-1.41562	4.37E-07	1.57E-05	down	_

orf00882-599	-1.36293	5.42E-07	1.94E-05	down	Biotin synthase
orf00189-124	1.401324	6.23E-07	2.20E-05	up	_
orf03244-2196	-1.36621	6.25E-07	2.20E-05	down	Elongation factor Ts
orf02896-1969	-1.37771	6.68E-07	2.34E-05	down	_
orf03511-2381	1.375058	7.89E-07	2.75E-05	up	Pyrroloquinoline quinone (Coenzyme PQQ) biosynthesis protein C-like protein
orf06278-4327	1.312624	8.12E-07	2.81E-05	up	_
orf00724-501	-1.32425	9.17E-07	3.16E-05	down	Uncharacterised protein
orf01981-1357	-1.32596	1.06E-06	3.63E-05	down	Glutamine synthetase
orf00722-500	-1.35986	1.09E-06	3.71E-05	down	Hypothetical protein K151_2921
orf00376-255	1.468297	1.09E-06	3.71E-05	up	Hypothetical protein YSA_06429
orf00449-307	1.312053	1.13E-06	3.80E-05	up	Chaperone protein ClpB
orf00736-509	-1.31303	1.17E-06	3.92E-05	down	Elongation factor Tu
orf06809-4712	1.337422	1.21E-06	4.03E-05	up	3-Oxoadipate CoA-transferase subunit A
orf01063-726	1.360572	1.24E-06	4.10E-05	up	LysM domain/BON superfamily protein
orf05820-3993	1.444055	1.32E-06	4.34E-05	up	_
orf00364-245	-1.40077	1.34E-06	4.39E-05	down	_
orf00007-5703	-1.53091	1.45E-06	4.74E-05	down	_
orf05433-3728	1.358972	1.49E-06	4.84E-05	up	_
orf07877-5401	-1.35239	1.53E-06	4.93E-05	down	_
orf04067-2760	1.357718	1.91E-06	6.13E-05	up	Uncharacterized protein ALO76_04692
orf07312-5038	1.392692	1.93E-06	6.16E-05	up	_
orf05459-3745	1.325164	1.95E-06	6.20E-05	up	_
orf07133-4926	-1.3129	1.97E-06	6.22E-05	down	_
orf00689-471	-1.29163	2.05E-06	6.44E-05	down	_
orf05737-3933	-1.30611	2.10E-06	6.55E-05	down	Saccharopine dehydrogenase
orf05176-3533	1.248297	2.17E-06	6.75E-05	up	_

orf03131-2123	-1.31497	2.19E-06	6.76E-05	down	_
orf07986-5470	1.316019	2.33E-06	7.17E-05	up	_
orf05069-3456	1.445109	3.20E-06	9.78E-05	up	Hypothetical protein T1E_5212
orf04553-3091	1.298261	3.29E-06	1.00E-04	up	_
orf04109-2788	-1.46373	3.55E-06	0.000107	down	_
orf00692-474	-1.34694	3.66E-06	0.00011	down	_
orf02481-1702	1.350502	4.61E-06	0.000138	up	_
orf06458-4456	1.27974	4.65E-06	0.000139	up	Hydroperoxidase, partial
orf04552-3090	1.26681	4.70E-06	0.000139	up	_
orf07134-4927	-1.24107	4.79E-06	0.000141	down	Succinate dehydrogenase flavoprotein subunit
orf03615-2456	-1.37722	5.37E-06	0.000158	down	_
orf02895-1968	-1.24956	5.41E-06	0.000158	down	50S ribosomal protein L13
orf06288-4335	1.222217	5.62E-06	0.000164	up	Hypothetical protein PPUTLS46_000315
orf00694-476	-1.23698	5.68E-06	0.000165	down	_
orf06293-4339	1.197791	6.66E-06	0.000192	up	Branched-chain amino acid ABC transporter substrate-binding protein
orf07864-5392	1.227943	6.74E-06	0.000193	up	_
orf03598-2442	-1.21538	7.33E-06	0.000209	down	_
orf03599-2443	-1.23318	7.97E-06	0.000226	down	_
orf08301-5688	-1.22184	8.23E-06	0.000233	down	Superoxide dismutase
orf04354-2956	-1.19717	8.95E-06	0.000252	down	Bifunctional aconitate hydratase 2/2-methylisocitrate dehydratase
orf02992-2032	1.178893	9.12E-06	0.000256	up	Hypothetical protein PAERUG_P50_London_9_VIM_2_01_ 13_05493
orf05807-3982	1.345016	9.19E-06	0.000256	up	_
orf00697-478	-1.1987	9.59E-06	0.000266	down	_
orf05843-4008	1.206029	9.79E-06	0.00027	up	_
orf00947-643	1.169956	9.87E-06	0.000271	up	_
orf00850-581	1.207371	1.03E-05	0.000283	up	_

orf02275-1552	1.203206	1.07E-05	0.000292	up	_
orf00695-477	-1.22968	1.09E-05	0.000294	down	_
orf07266-5007	1.217698	1.10E-05	0.000297	up	_
orf02055-1406	1.193354	1.12E-05	0.000302	up	_
orf08142-5583	-1.17169	1.14E-05	0.000306	down	_
orf04645-3164	-1.22573	1.15E-05	0.000306	down	Transcriptional regulator
orf03857-2620	-1.20256	1.20E-05	0.000317	down	_
orf00698-479	-1.20928	1.30E-05	0.000344	down	_
orf05449-3739	1.207623	1.31E-05	0.000344	up	_
orf01617-1117	-1.1829	1.33E-05	0.000349	down	Aldehyde dehydrogenase
orf01296-891	1.266608	1.34E-05	0.00035	up	Hypothetical protein YSA_05171
orf03406-2307	-1.36543	1.37E-05	0.000355	down	Methyl-accepting chemotaxis sensory transducer
orf02478-1699	-1.39152	1.47E-05	0.00038	down	_
orf01491-1023	-1.17244	1.56E-05	0.000402	down	_
orf06290-4337	1.170115	1.58E-05	0.000404	up	_
orf01221-833	-1.17388	1.64E-05	0.000419	down	_
orf05742-3936	1.228694	1.67E-05	0.000423	up	_
orf04951-3374	-1.25809	1.78E-05	0.000449	down	_
orf01911-1314	-1.16621	1.79E-05	0.000452	down	_
orf02485-1705	1.14005	2.05E-05	0.000515	up	Hypothetical protein
orf07316-5041	1.172554	2.07E-05	0.000517	up	_
orf07265-5006	1.16344	2.22E-05	0.000553	up	Cytochrome c oxidase, cbb3-type subunit I
orf05641-3863	1.261745	2.24E-05	0.000555	up	Hypothetical protein
orf00726-502	-1.13906	2.25E-05	0.000556	down	DNA-directed RNA polymerase subunit beta
orf06885-4764	1.150759	2.61E-05	0.000643	up	Zn-dependent hydrolase
orf05434-3729	1.155497	2.69E-05	0.000658	up	_

orf03529-2391	-1.12495	2.80E-05	0.000683	down	_
orf06742-4658	-1.14288	3.17E-05	0.00077	down	Hypothetical protein
orf00210-140	-1.17826	3.56E-05	0.00086	down	1-Phosphofructokinase
orf03451-2337	1.117037	3.67E-05	0.000884	up	_
orf03628-2465	-1.32709	3.85E-05	0.000923	down	_
orf04862-3314	-1.12879	4.08E-05	0.000975	down	Hybrid sensor histidine kinase/response regulator
orf03526-2389	-1.10884	4.20E-05	0.001	down	_
orf02606-1785	-1.10624	4.23E-05	0.001003	down	Translation initiation factor IF-2
orf06810-4713	1.144739	4.27E-05	0.001008	up	3-Oxoadipate CoA-transferase subunit B
orf02057-1407	1.153559	4.36E-05	0.001025	up	ATP-dependent protease subunit HslV
orf05447-3738	1.116859	4.85E-05	0.001135	up	_
orf00699-480	-1.09348	5.24E-05	0.001219	down	50S ribosomal protein L6
orf01175-805	-1.14858	5.25E-05	0.001219	down	Cystathionine gamma-synthase
orf02243-1531	-1.102	5.36E-05	0.001241	down	_
orf07461-5130	-1.12773	5.56E-05	0.001282	down	_
orf07564-5195	1.605083	5.77E-05	0.001326	up	_
orf00450-308	1.090909	5.80E-05	0.001328	up	_
orf07041-4866	-1.10298	5.87E-05	0.001339	down	_
orf03629-2466	-1.35037	6.30E-05	0.00143	down	_
orf04555-3092	1.142907	6.54E-05	0.001479	up	_
orf07479-5140	-1.08705	6.58E-05	0.001483	down	_
orf06274-4323	1.095211	6.64E-05	0.001491	up	Alcohol dehydrogenase
orf03683-2504	-1.09652	6.84E-05	0.001531	down	_
orf00150-5826	1.226015	7.25E-05	0.001615	up	_
orf08161-5596	1.081596	7.50E-05	0.001666	up	_
orf00873-593	-1.06612	7.56E-05	0.001672	down	Acyl-CoA dehydrogenase

orf05817-3991	1.094338	7.63E-05	0.001682	up	_
orf07475-5138	-1.05471	8.16E-05	0.001792	down	_
orf06568-4530	-1.18182	8.28E-05	0.001812	down	_
orf05819-3992	1.197653	8.48E-05	0.001847	up	Hypothetical protein
orf04323-2939	-1.08347	8.50E-05	0.001847	down	Phospho-2-dehydro-3-deoxyheptonate aldolase
orf02611-1788	-1.11589	8.62E-05	0.001862	down	_
orf02997-2036	1.087555	8.63E-05	0.001862	up	3-Oxoadipate enol-lactonase
orf07541-5179	-1.07655	8.80E-05	0.001891	down	Aspartate kinase
orf02876-1955	-1.06041	8.90E-05	0.001906	down	_
orf08121-5569	1.196209	8.94E-05	0.001907	up	Hypothetical protein
orf03130-2122	-1.13432	9.25E-05	0.001967	down	RNA-binding protein
orf06713-4638	1.232261	9.36E-05	0.001984	up	_
orf06915-4780	1.112448	9.40E-05	0.001984	up	Hypothetical protein T1E_3306
orf02276-1553	1.213123	9.64E-05	0.002027	up	_
orf07036-4863	-1.14983	0.000105	0.002193	down	NADH-quinone oxidoreductase subunit K
orf07040-4865	-1.05871	0.000105	0.002201	down	NADH:ubiquinone oxidoreductase subunit M
orf04481-3043	1.029114	0.000106	0.002209	up	_
orf06856-4746	1.207076	0.000107	0.002221	up	_
orf03522-2387	-1.05611	0.000119	0.002456	down	P-protein
orf01726-1187	1.099746	0.00013	0.002681	up	Hypothetical protein
orf07119-4915	1.02686	0.000131	0.002684	up	Molecular chaperone HtpG
orf07567-5197	-1.10609	0.000133	0.002731	down	_
orf03524-2388	-1.04975	0.000136	0.002774	down	Prephenate dehydratase
orf00275-187	-1.1257	0.000137	0.002776	down	_
orf03097-2101	1.027346	0.00014	0.002827	up	Lon protease
orf07488-5145	-1.05024	0.000146	0.002945	down	_

orf07983-5468	1.074611	0.000146	0.002945	up	_
orf00169-117	-1.02129	0.000156	0.003127	down	Cytochrome ubiquinol oxidase subunit I
orf03159-2141	1.018055	0.00017	0.003406	up	_
orf03837-2609	-1.02265	0.000177	0.003522	down	Aspartate-semialdehyde dehydrogenase
orf02578-1768	1.030573	0.000182	0.003605	up	_
orf02967-2014	1.054493	0.000188	0.003716	up	_
orf07138-4930	-1.00761	0.00019	0.003745	down	Type II citrate synthase
orf05163-3525	0.984996	0.000191	0.003745	up	_
orf06938-4796	1.121161	0.000191	0.003745	up	_
orf01136-777	-1.01524	0.000196	0.00382	down	5-aminovalerate aminotransferase DavT
orf04310-2930	-1.09645	0.000199	0.003878	down	_
orf07154-4941	-1.11336	0.000202	0.00391	down	_
orf05441-3734	1.02516	0.000204	0.003949	up	_
orf04379-2975	1.015829	0.000208	0.004007	up	_
orf00167-116	-1.04376	0.000209	0.004016	down	Cytochrome o ubiquinol oxidase
orf05435-3730	1.012634	0.00021	0.004016	up	LysR family transcriptional regulator
orf03854-2619	-1.00966	0.00021	0.004016	down	Amidophosphoribosyltransferase
orf07292-5025	-1.12638	0.000223	0.004223	down	_
orf07026-4858	-1.00579	0.00023	0.004331	down	NADH dehydrogenase I subunit F
orf04357-2959	-1.08432	0.00023	0.004331	down	_
orf04705-3204	-1.10756	0.000232	0.004354	down	_
orf02404-1638	1.016108	0.000241	0.004485	up	_
orf07799-5345	-1.00737	0.000255	0.004728	down	Dihydrodipicolinate synthase
orf06750-4665	1.009641	0.000255	0.004728	up	_
orf01075-735	1.002182	0.000269	0.00495	up	Phosphoesterase
orf03158-2140	1.051552	0.00027	0.004955	up	DNA repair protein HhH-GPD

orf03785-2573	-1.12245	0.000271	0.004955	down	50S ribosomal protein L32
orf01908-1312	-1.03458	0.000303	0.00548	down	Ribosomal protein L31
orf01575-1085	-1.06734	0.00034	0.006033	down	Hypothetical protein
orf07135-4928	-1.01044	0.000344	0.006081	down	Succinate dehydrogenase
orf03597-2441	-1.00042	0.000364	0.006335	down	_
orf07267-5008	1.035438	0.000384	0.006614	up	_
orf00166-115	-1.02383	0.000389	0.00666	down	_
orf05779-3964	-1.05873	0.000394	0.006734	down	Hypothetical protein
orf07695-5280	-1.02088	0.000407	0.006879	down	_
orf06751-4666	1.014384	0.000419	0.007042	up	_
orf05672-3886	1.034134	0.003402	0.039999	up	Short-chain dehydrogenase

log_2FC, log_2 fold change. FDR, false discovery rate.

TABLE S6 Sequences of *sRNA* genes

Gene name	Sequence (5'-3')
sRNA 8	CGGGTTCTGGGGAGGAATCCGGGTTAAGACCATTAGGAGTAAAACAAAGGTACGCGGTCCCTGAGCACC TTTATCGGCGCGCCACTGGGGGGATGCGCCGCGCCAACACTTCAAGTATTGGTCAGGTTTGGCGCAGTGC CAGTGCATATTGGACGTTTTTTAAACAGATTTGGAATACGCCAACGTTTCGTT
sRNA 11	TGCCCTGAAATGGCACATCTACGGCAGATTTGCTGCATATCGGGGCAATCCATTATCCGCTATTTCCCGGGT AAAGGGCTATCTCCGGTGCGCCGTGTTTTATTTTTTACGGAGGTGTTGTGTAAAAAAATGAACAAGGCTAA GCTCGGTTTCAACCCAAAACACAATAATACGAGGGT
sRNA 14	TCCGGGCAAAAAAAACCCCGGACTTCGTATGGGGAGGGGGAAGTTCGGGGTCCAAGTCCGGACCGCTAG GGCGGGGTCCAGATATCTGCCAACACTTAACACAACATAGGAGCAT
sRNA 20	TGACTGCCAAATGATAACGATTATTATTGCACTCAGCTGATCGCGAGATCCGCTGGATAACCTGAAAGCTT AGGTCGCTCTCAGATTATCTCCTCATCAGGCTAATCACGGTTTTTGACCCGGCTTTTTTGCCGGGTCTTTTT TTT
sRNA 60	ATGGTGGCAGCTGTACGCGGGTTGACAGACCGAGACGTTGGCACTCGGCGCACCACAGGGTGCCCACAC GTACAGCCGCCATGAAA

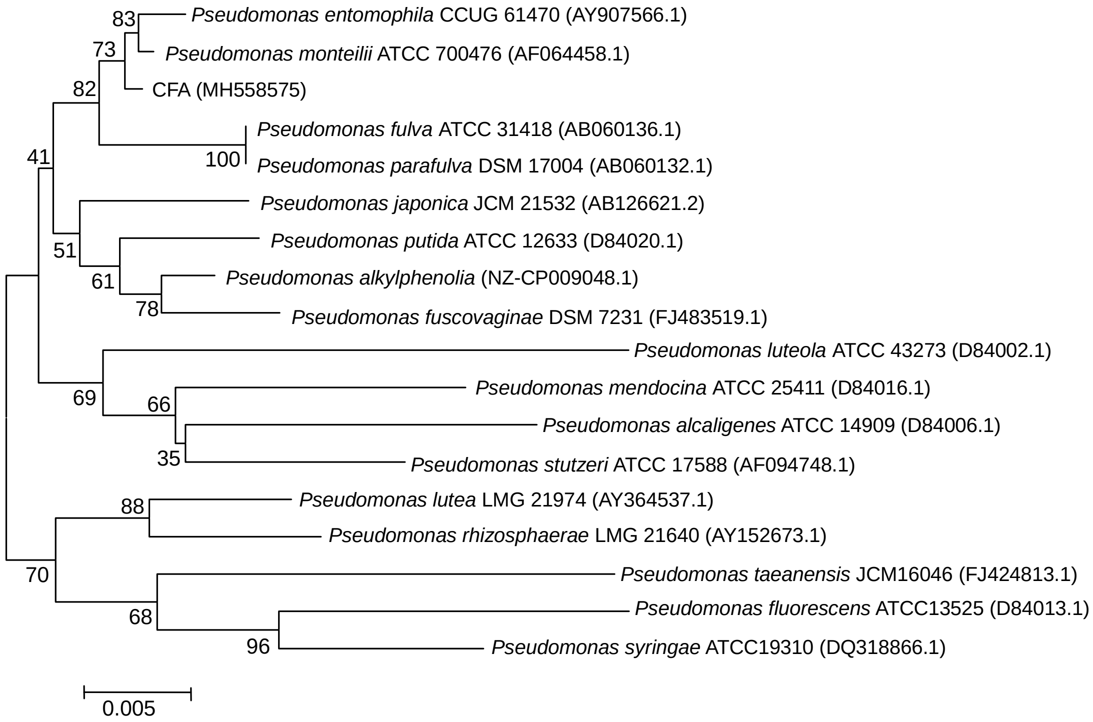

FIGURE S1 Phylogenetic tree obtained by neighbor-joining analysis of 16S rRNA gene sequences. Bootstrap values of > 30% from 1000 bootstrap replicates are indicated above the branch nodes, and the scale bar represents 0.005 substitutions per nucleotide position.

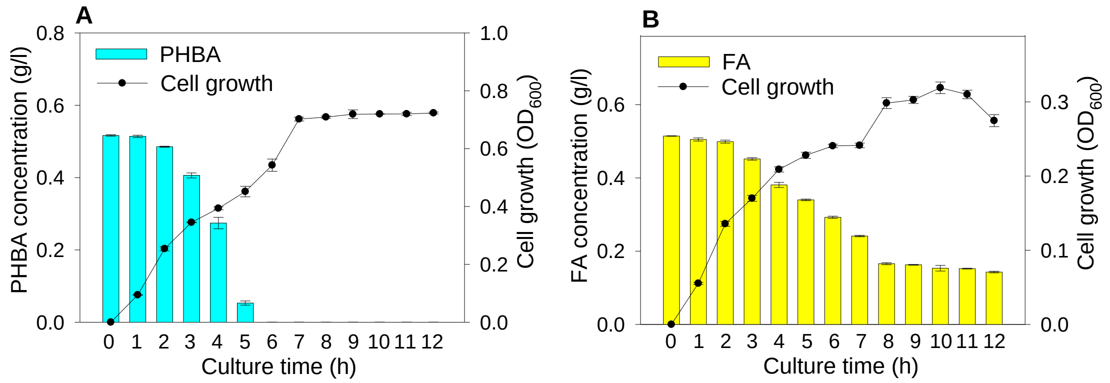

FIGURE S2 PHBA (**A**) and FA (**B**) degradation by CFA in liquid M-9 medium with 0.5 g/l PHBA or FA as the sole carbon source, respectively. Bars represent standard errors of triplicate experiments.

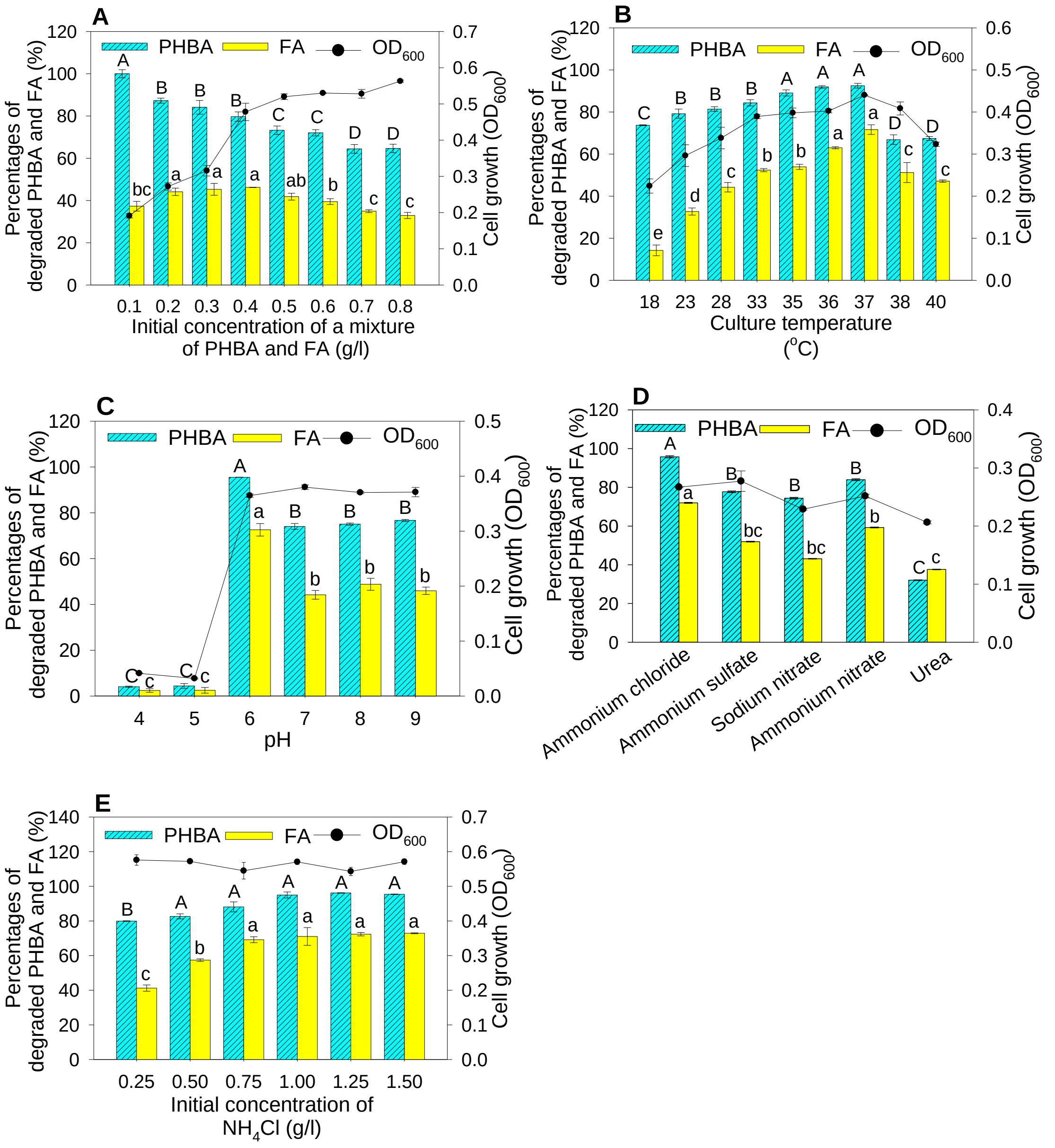

FIGURE S3 Effects of initial concentration of the mixture of PHBA and FA (**A**), culture temperature (**B**), pH (**C**), and nitrogen source (**D**, **E**) on PHBA and FA degradation by CFA. For percentages of degraded PHBA, values with the different upper case letters are significantly different at $P < 0.05$, while values with the different lower case letters are significantly different at $P < 0.05$ for percentages of degraded FA.

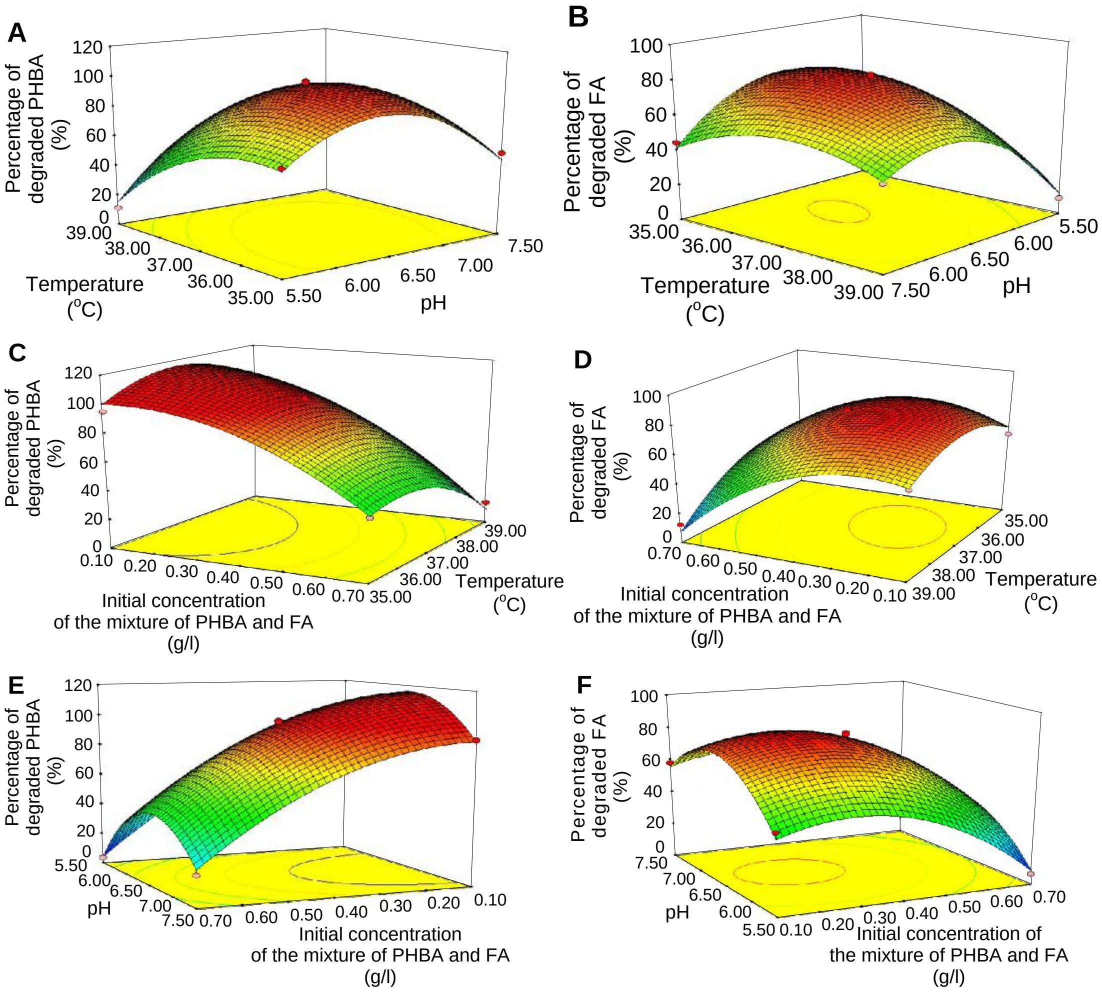

FIGURE S4 Response surface plot for PHBA and FA degradation by CFA in terms of the effects of temperature and pH (**A**, **B**), temperature and concentration of the mixture of PHBA and FA (**C**, **D**), and pH and concentration of the mixture of PHBA and FA (**E**, **F**). Factors that were not included in the axes were fixed at their respective optimum levels.

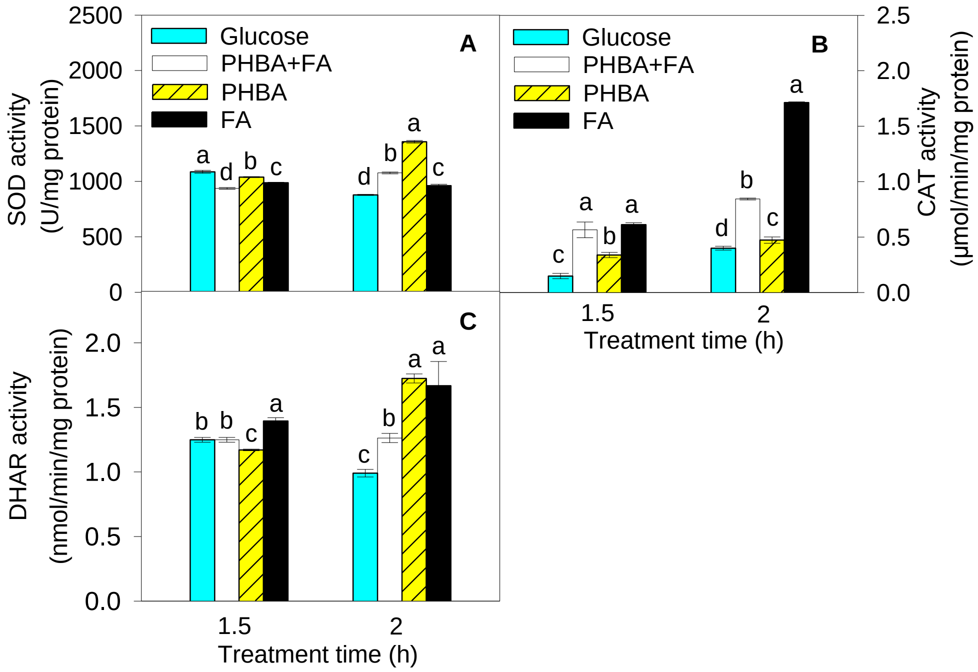

FIGURE S5 Activities of SOD (**A**), CAT (**B**), and DHAR (**C**) in CFA. Glucose, cultured in glucose; PHBA+FA, cultured in the mixture of PHBA and FA; PHBA, cultured in PHBA; FA, cultured in FA. Bars represent standard errors of triplicate experiments. At each treatment time, values with the different letters are significantly different at $P < 0.05$.

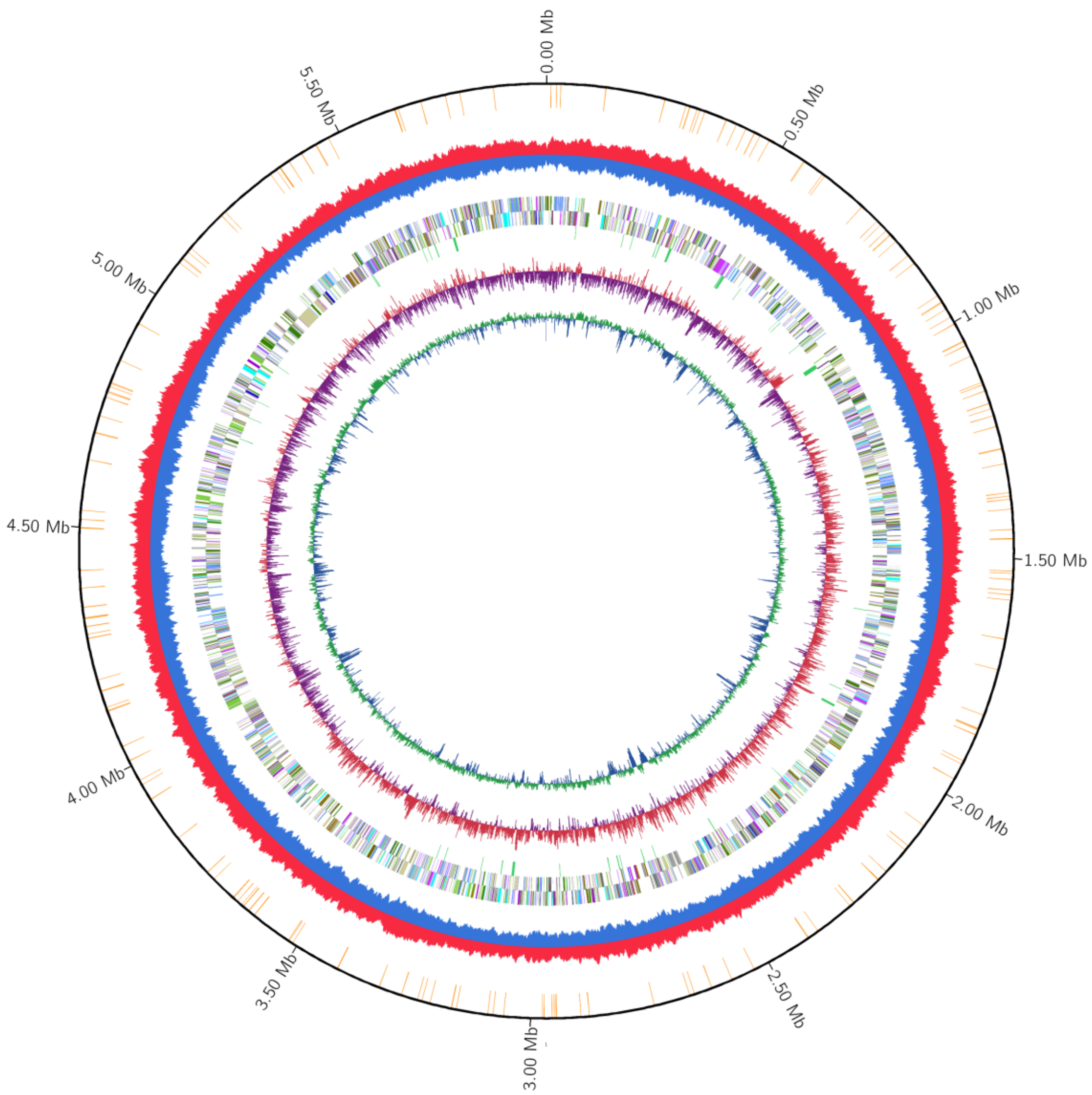

FIGURE S6 Schematic representation of the circular chromosome. The scales indicate location in Mbp, starting with the initial coding region. From inner to outer circles: the first circle shows the GC content, and the values are plotted as the deviation from the average GC content of the entire chromosome sequence; the values toward the outside are above the average GC content, and the ones toward the inside are below the average GC content. The bars in the second circle represent the GC skew; the bars toward the inside are below zero, and the ones toward the outside are above zero. Positions of ncRNA, tRNA, and rRNA are marked by bars in the third circle. Bars in the fourth and fifth circle are marked according to COG function

categories of CDS; the fourth is a backward strand and the fifth is a forward strand. The sixth and seventh circles indicate m4C and m6A sites in CDS/rRNA/tRNA regions; the sixth circle is a backward strand, and the seventh circle is a forward strand. In the eighth circle, the bars show the genes involved in restriction-modification systems.

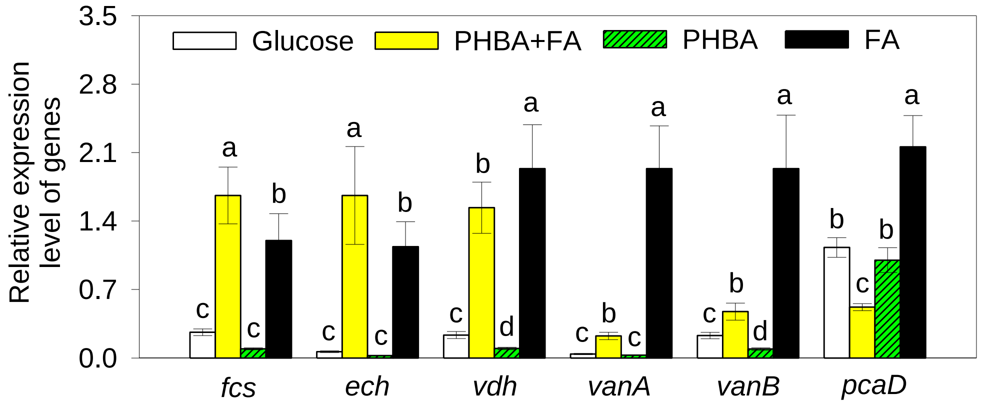

FIGURE S7 Relative expression levels of *fcs*, *ech*, *vdh*, *vanA*, *vanB*, and *pcaD* in CFA after 1.5 hours of cultivation. Glucose, cultured in glucose; PHBA+FA, cultured in the mixture of PHBA and FA; PHBA, cultured in PHBA; FA, cultured in FA. Bars represent standard errors of triplicate experiments. For each gene, values with the different letters are significantly different at $P < 0.05$.

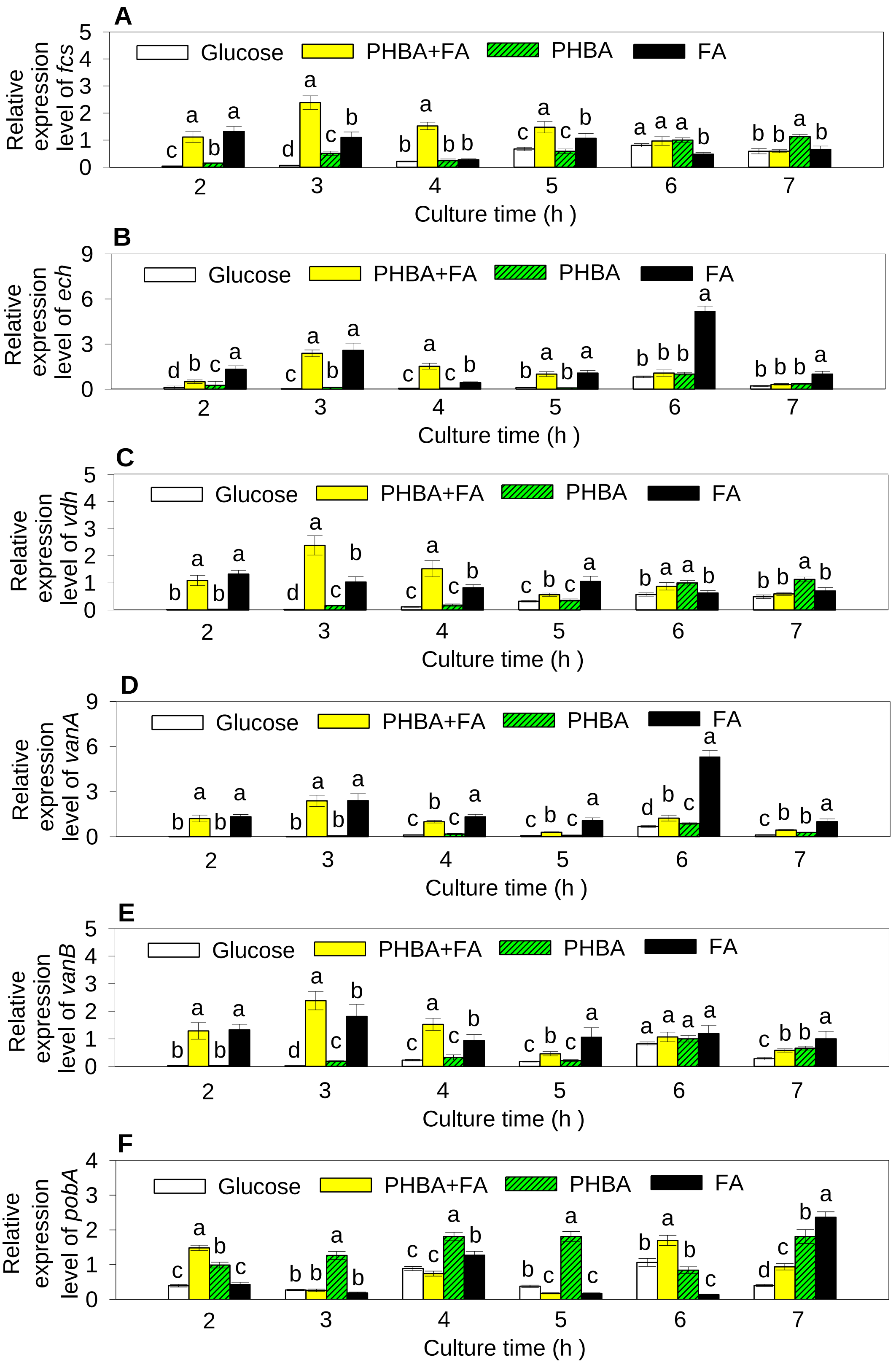

FIGURE S8 Relative expression levels of *fcs* (**A**), *ech* (**B**), *vdh* (**C**), *vanA* (**D**), *vanB* (**E**), and *pobA* (**F**) in CFA at 2-7 h. At each treatment time, values with the different letters are significantly different at $P < 0.05$.

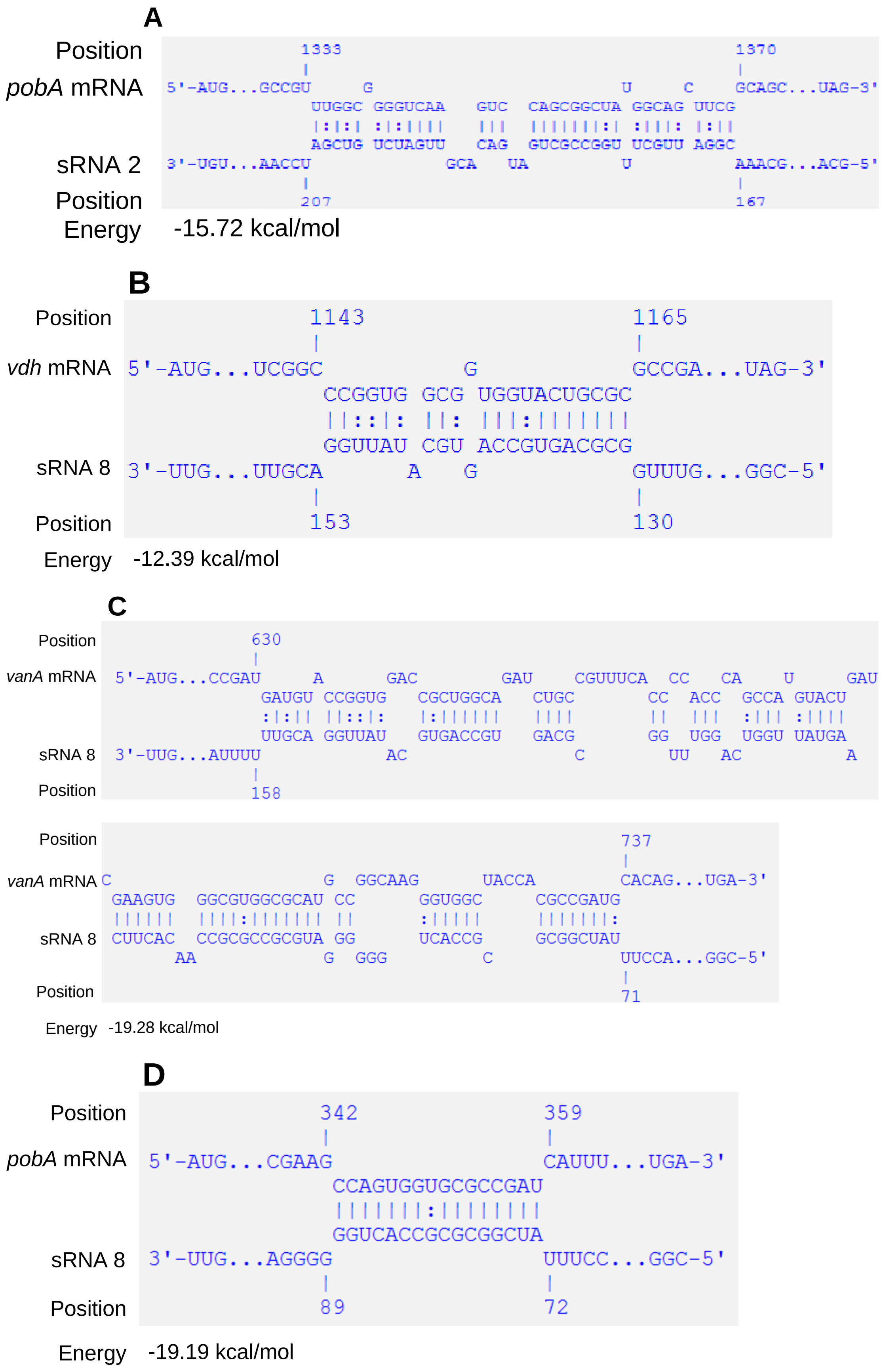
A
Position 1333 1370
pobA mRNA 5'-AUG...GCCGU G U C GCAGC...UAG-3'
UUGGC GGGUCAA GUC CAGCGGCUA GGCAG UUCG
|:|:| :|:|||| ||| |||||||:| :|||: |:||
AGCUG UCUAGUU CAG GUCGCCGGU UCGUU AGGC
sRNA 2 3'-UGU...AACCU GCA UA U AAACG...ACG-5'
Position 207 167
Energy -15.72 kcal/mol
B
Position 1143 1165
vdh mRNA 5'-AUG...UCGGC G GCCGA...UAG-3'
CCGGUG GCG UGGUACUGCGC
||::|: ||: |||:|||||||
GGUUAU CGU ACCGUGACGCG
sRNA 8 3'-UUG...UUGCA A G GUUUG...GGC-5'
Position 153 130
Energy -12.39 kcal/mol
C
Position 630
vanA mRNA 5'-AUG...CCGAU A GAC GAU CGUUUCA CC CA U GAU
GAUGU CCGGUG CGCUGGCA CUGC CC ACC GCCA GUACU
:|:|| ||::|: |:|||||| |||| || ||| :||| :||||
UUGCA GGUUAU GUGACCGU GACG GG UGG UGGU UAUGA
sRNA 8 3'-UUG...AUUUU AC C UU AC A
Position 158
Position 737
vanA mRNA C G GGCAAG UACCA CACAG...UGA-3'
GAAGUG GGCGUGGCGCAU CC GGUGGC CGCCGAUG
|||||| ||||:||||||| || :||||| |||||||:
sRNA 8 CUUCAC CCGCGCCGCGUA GG UCACCG GCGGCUAU
AA G GGG C UUCCA...GGC-5'
Position 71
Energy -19.28 kcal/mol
D
Position 342 359
pobA mRNA 5'-AUG...CGAAG CAUUU...UGA-3'
CCAGUGGUGCGCCGAU
|||||||:||||||||
GGUCACCGCGCGGCUA
sRNA 8 3'-UUG...AGGGG UUUCC...GGC-5'
Position 89 72
Energy -19.19 kcal/mol

E

```
Position                31                                          75
                        |                                           |
pcaH mRNA   5'-AUG...CGUGA    UG  C  A        CCUAAA   CUUAC         CAAGA...UGA-3'
                          UCCG  AU GC ACUGGCAC      GCC     GCCUGACUA
                          |||:  || || ||||||||      |||     :||||||:|
                          AGGU  UA CG UGACCGUG      CGG     UGGACUGGU
sRNA 8      3'-UUG...UUUGC    UA              ACG       UU           UAUGA...GGC-5'
                        |                                           |
Position                154                                         118
```

Energy -10.97 kcal/mol

F

```
Position                356           370
                        |             |
pcaG mRNA   5'-AUG...GCUGC             GCGCC...UGA-3'
                          UGGCGUGCCGAUG
                          |||||:||||||:
                          ACCGCGCGGCUAU
sRNA 8      3'-UUG...GGGUC             UUCCA...GGC-5'
                        |             |
Position                85            71
```

Energy -9.99 kcal/mol

G

```
Position                845                                                    897
                        |                                                      |
pcaB mRNA   5'-AUG...CACAA     A          G   C     UGU   UC      GC      C       GGGCC...UGA-3'
                          GCGCA    CCCGGUG GUG CGCCG   UGA  GGUGCU    GACC GCGUGCC
                          |||||    |||:||| |:| |||||   |:|  ||||||    |||| ||||:||
                          CGCGU    GGGUCAC CGC GCGGC   AUU  CCACGA    CUGG CGCAUGG
sRNA 8      3'-UUG...CGCGC     AGGG                 U     U       GUCC            AAACA...GGC-5'
                        |                                                      |
Position                99                                                     48
```

Energy -14.63 kcal/mol

H

```
Position                710
                        |
pcaD mRNA   5'-GUG...GUCGA        C     ACCUGUCCAAC     GAUUGGC    CC     CC          C
                          CUUCCCGG GGCGC           GUCGA       GAGG  UUCAG  GCCGCGUGCU
                          |::|||:| |||||           |:|||       :|||  :||||  :||||||:|:
                          GGGGGGUC CCGCG           CGGCU       UUCC  GAGUC  UGGCGCAUGG
sRNA 8      3'-UUG...GCGUA        A                     AU         AC     CC          A
                        |
Position                93

Position                         788
                                 |
pcaD mRNA   GAU           C   C  ACUGA-3'
                    UUCCUG UGG UC
                    :||||: ||| ||
                    GAGGAU ACC AG
sRNA 8      AACAAAAU      U      AAUUG...GGC-5'
                                 |
Position                         27
```

Energy -15.69 kcal/mol

I

```
Position      	             495
              	             |
ACAT mRNA     	5'-UUG...UGGAC   AU           GA          C    CUA          C        GAAA       A
              	              AUG  C GGUACUGCG  AAACCUGGCC GUGC         UGGC UGGCGCGU     CGGUGG CG
              	              |||  | ||:||||||  |||||||:|| :|:|         |||| :|||||:|     |:|||| ||
              	              UAC  G CCGUGACGC  UUUGGACUGG UAUG         ACCG GCCGCGUA     GUCACC GC
sRNA 8        	3'-UUG...GGUUA   GU A         GG          U    AACUUCACA    C        GGGGG
              	             |
Position      	             148
```

```
Position      	      563
              	      |
ACAT mRNA     	   G  CCCUG...UGA-3'
              	CGU GG
              	||: |:
              	GCG CU
sRNA 8        	   G  AUUUC...GGC-5'
              	      |
Position      	      73
```

Energy -15.69 kcal/mol

J

```
Position      	             839           853
              	             |             |
vdh mRNA      	5'-AUG...AACCA G           AUGUC...UAG-3'
              	              G GGCAGAUCUGC
              	              | :|||:||||||
              	              C UCGUUUAGACG
sRNA 11       	3'-UGG...CUAUA G           GCAUC...CGU-5'
              	             |             |
Position      	             37            23
```

Energy -10.08 kcal/mol

K

```
Position      	             728                           758
              	             |                             |
pobA mRNA     	5'-AUG...GAACU  A     G        CC          CGGAC...UGA-3'
              	              GA AGCCC UUUGCCCG  GAGGUGGCGG
              	              || ||||| |||:||||  ||::|:||||
              	              CU UCGGG AAAUGGGC  CUUUAUCGCC
sRNA 11       	3'-UGG...GGCCU  A              C           UAUUA...CGU-5'
              	             |                             |
Position      	             84                            56
```

Energy -11.11 kcal/mol

L

```
Position      	             24                41
              	             |                 |
pcaJ mRNA     	5'-AUG...UCUCC                 AACGC...UGA-3'
              	              CGCACCG AGAUGGCCC
              	              ||||||| ||||:||||
              	              GCGUGGC UCUAUCGGG
sRNA 11       	3'-UGG...GUGCC       C         AAAUG...CGU-5'
              	             |                 |
Position      	             93                75
```

Energy -12.18 kcal/mol

M

```
Position                951           965
                        |             |
pcaF mRNA   5'-AUG...UCGAC U            ACGAA...UGA-3'
                          G GAUCGAGCUUA
                          | :|:||||||||
                          C UUGGCUCGAAU
sRNA 11     3'-UGG...CCCAA U            CGGAA...CGU-5'
                         |             |
Position                 154           140
```

Energy -10.46 kcal/mol

N

```
Position               992                       1018
                       |                         |
fcs mRNA    5'-GUG...GUCAA    CUGGAG A         G   CGCGA...UGA-3'
                          GGCA      C GGACCCCGC CUG
                          ||||      | ||||||||| ||:
                          CCGU      G CCUGGGGCG GAU
sRNA 14     3'-UAC...CACAA    CUAUA  A         G   CGCCA...CCU-5'
                         |                         |
Position                 91                        66
```

Energy -11.83 kcal/mol

O

```
Position          4                                            33
                  |                                            |
ech mRNA    5'-AUGA  AAA    AA                CG           A    GAAGG...UGA-3'
                   GC   UAUG                  GGC      CUGGACC CCGU
                   ||   ||||                  |||      ||||||| |||:
                   CG   AUAC                  CCG      GACCUGG GGCG
sRNA 14       3'-UA  AGG    AACACAAUUCACAA   UCUAUA            GGAUC...CCU-5'
                  |                                            |
Position          114                                          70
```

Energy -9.08 kcal/mol

P

```
Position                 167               183
                         |                 |
vdh mRNA    5'-AUG...CCGGC                 ACCGG...UAG-3'
                          CUGGGCC  GCCCUGGC
                          ||||:||  |||||:||
                          GACCUGG  CGGGAUCG
sRNA 14     3'-UAC...CUAUA       GG        CCAGG...CCU-5'
                         |                 |
Position                 82                64
```

Energy -10.11 kcal/mol

Q

```
Position                 253                          282
                         |                            |
pobA mRNA   5'-AUG...CCAGC           AAA     GACC     CAAGA...UGA-3'
                          GUCUGGAUCUC   GCCCU    GGCGG
                          :||||||:|:|   |||||    :||||
                          UAGACCUGGGG   CGGGA    UCGCC
sRNA 14     3'-UAC...GUCUA                            AGGCC...CCU-5'
                         |                            |
Position                 84                           62
```

Energy -10.11 kcal/mol

R

```
Position                 476                              507
                         |                                |
pcaH mRNA   5'-AUG...CGCCC    C     C          AUC        AUCGA...UGA-3'
                          GGCG AUAUC   ACUUCGCC   AGCGGUCC
                          |||: |||||   ||::||||   ||||||||
                          CCGU UAUAG   UGGGGCGG   UCGCCAGG
sRNA 14     3'-UAC...CACAA    C     ACC        GA         CCUGA...CCU-5'
                         |                                |
Position                 91                               59
```

Energy -11.62 kcal/mol

S

```
Position                 46                                            74
                         |                                             |
pcaG mRNA   5'-AUG...CGGCC    C                   C            G       AAGCC...UGA-3'
                          CCUA GU              GCA   AUC GG CCU GCCCUGG
                          |||| ||              |||   ||| || ||: |||||:|
                          GGAU CA              CGU   UAG CC GGG CGGGAUC
sRNA 14           3'-UACGA    A  ACACAAUUCACAAC   CUA   A  U   G       GCCAG...CCU-5'
                         |                                             |
Position                 111                                           65
```

Energy -11.60 kcal/mol

T

```
Position                 953
                         |
pcaB mRNA   5'-AUG...CUGGG      GCA  CC         AAACCCUGC          CU
                          CCUAUG   UG  GAGUG  GG         CGGAUAUCUG
                          ||||||   ||  :||||  ||         |:||||||||
                          GGAUAC   AC  UUCAC  CC         GUCUAUAGAC
sRNA 14           3'-UACGA      AAC  AA     AA                     C
                         |
Position                 111
```

```
Position                   1010
                           |
                G   U      CCCUG...UGA-3'
pcaB mRNA   GCCU GUC CUGGCG
            :||: |:| ||:|||
sRNA 14     UGGG CGG GAUCGC
                G          CAGGC...CCU-5'
                           |
Position                   63
```

Energy -13.04 kcal/mol

U

```
Position                 138                    159
                         |                      |
CMD1 mRNA    5'-AUG...CCUGG      C              GGGGC...UAA-3'
                           GGU GA AUCUGGACCC GCCC
                           ||: || |||||||||| ||||
                           CCG CU UAGACCUGGG CGGG
sRNA 14      3'-UAC...CACAA   U  A          G   AUCGC...CCU-5'
                          |                      |
Position                  91                     68
```

Energy -11.62 kcal/mol

V

```
Position                 119           133
                         |             |
pcaD mRNA    5'-GUG...ACCCA             AGCCA...UGA-3'
                           GAUUCCGCUCUGG
                           ||::||||:||:|
                           CUGGGGCGGGAUC
sRNA 14      3'-UAC...UAGAC             GCCAG...CCU-5'
                          |              |
Position                  79             65
```

Energy -7.85 kcal/mol

W

```
Position                 390                          419
                         |                            |
pcaJ mRNA    5'-AUG...CCGUC     UGCAA       G   A C    CCCGC...UGA-3'
                           GGCGG     UGGACCU GCC C GGCG
                           |||:|     ||||||: ||| | :|||
                           CCGUC     ACCUGGG CGG G UCGC
sRNA 14      3'-UAC...CACAA     UAUAG       G     A    CAGGC...CCU-5'
                          |                            |
Position                  91                           63
```

Energy -11.00 kcal/mol

X

```
Position                 1146                                 1181
                         |                                    |
pcaF mRNA    5'-AUG...GCCUG  GA C                C   CAAG      CGUUG...UGA-3'
                           GC  C AUG UGUG UGGGUGU GGC    GUCUGG
                           ||  | ||| |||| |::|||| |||    :|||||
                           CG  G UAC ACAC AUUCACA CCG    UAGACC
sRNA 14               3'-UA  AG A   A    A       A   UCUA      UGGGG...CCU-5'
                          |                                    |
Position                  114                                  77
```

Energy -13.09 kcal/mol

Y

```
Position                 526                                              574
                         |                                                |
ACAT mRNA   5'-UUG...CCGUG      GCC  GC C   AAAC      CGCG          G     CAUCA...UGA-3'
                          CCUAUG   UG  G GUG    GGUGGA    UGGGCCCUGC C AGC
                          ||||||   ||  : |||    ||::||    |||:|||:|| | |||
                          GGAUAC   AC  U CAC    CCGUCU    ACCUGGGGCG G UCG
sRNA 14           3'-UACGA      AAC  AA U   AA        AUAG          G A   CCAGG...CCU-5'
                         |                                                |
Position                 111                                              64
```

Energy -11.73 kcal/mol

Z

```
Position                 546                 566
                         |                   |
ech mRNA    5'-AUG...AGGCU    A   G          ACGAC...UGA-3'
                          GCCG GAU GGCCUGGUGA
                          :||| ||| :|||||:|||
                          UGGC CUA UCGGACUACU
sRNA 20     3'-UUU...GUUUU    A   A          CCUCU...AGU-5'
                         |                   |
Position                 113                 93
```

Energy -13.69 kcal/mol

A1

```
Position                 195                 214
                         |                   |
pobA mRNA   5'-AUG...GCAUG        A          CACGA...UGA-3'
                          GACCGUGA  GGCCUGGUG
                          :|||||||  :|||||:||
                          UUGGCACU  UCGGACUAC
sRNA 20     3'-UUU...AGUUU        AA         UCCUC...AGU-5'
                         |                   |
Position                 114                 94
```

Energy -15.43 kcal/mol

B1

```
Position                 751               769
                         |                 |
pcaB mRNA   5'-AUG...GUUCG        AUC      CUGAU...UGA-3'
                          GCCGUGAU   AGCUUG
                          :|||||||   |||:||
                          UGGCACUA   UCGGAC
sRNA 20     3'-UUU...GUUUU        A        UACUC...AGU-5'
                         |                 |
Position                 113               97
```

Energy -10.58 kcal/mol

C1

```
Position                 17           28
                         |            |
pcaF mRNA   5'-AUG...UUCAU            AUCCG...UGA-3'
                          CUGUGAU  GCC
                          |:|||||  |||
                          GGCACUA  CGG
sRNA 20     3'-UUU...UUUUU       AU   ACUAC...AGU-5'
                         |            |
Position                 112          99
```

Energy -8.97 kcal/mol

D1

```
Position                   1218                    1242
                           |                       |
ACAT mRNA     5'-UUG...UGAUC       CUCAUUUCGUU      -3'
                            GAAAACC           GCCUGA
                            :||||||           ||||||
                            UUUUUGG           CGGACU
sRNA 20       3'-UUU...CCCAG       CACUAAU          ACUCC...AGU-5'
                           |                        |
Position                   117                      96
```

Energy -7.45 kcal/mol

E1

```
Position                   1218                    1242
                           |                       |
fcs mRNA      5'-UUG...UGAUC       CUCAUUUCGUU      -3'
                            GAAAACC           GCCUGA
                            :||||||           ||||||
                            UUUUUGG           CGGACU
sRNA 60       3'-UUU...CCCAG       CACUAAU          ACUCC...AGU-5'
                           |                        |
Position                   117                      96
```

Energy -7.45 kcal/mol

F1

```
Position                   1190                              1222
                           |                                 |
vdh mRNA      5'-AUG...CUCCA              AC  GGAGU          UCGGC...UAG-3'
                            GCUG   GCCAACG UC      UCGGUCUGUCA
                            ||:|   ||||||| ||      |||||||||||
                            CGGC   CGGUUGC AG      AGCCAGACAGU
sRNA 60       3'-AAA...CCACG    UCA                          UGGGC...GUA-5'
                           |                                 |
Position                   50                                22
```

Energy -10.75 kcal/mol

G1

```
Position                   747     755
                           |       |
vanB mRNA     5'-AUG...GGACC       AGGUG...UGA-3'
                            GUGGUGC
                            |||||||
                            CACCACG
sRNA 60       3'-AAA...UGGGA       CGGCU...GUA-5'
                           |       |
Position                   57      49
```

Energy -10.03 kcal/mol

H1

```
Position                561                                                611
                        |                                                  |
pcaH mRNA    5'-AUG...UGAUC    U     CUAU    AAG   A  GC         AAGCC       AGUUG...UGA-3'
                           CCGA GUGCC    CGUC   UCG UC   CAACCCGC     GUGCAGC
                           |||| |||||    ||||   ||| ||   ||||||||     ||:||||
                           GGCU CACGG    GCAG   AGC AG   GUUGGGCG     CAUGUCG
sRNA 60      3'-AAA...CACGC          UU            C  ACA                    ACGGU...GUA-5'
                        |                                                  |
Position                49                                                 9
```

Energy -13.68 kcal/mol

I1

```
Position                187      196
                        |        |
pcaG mRNA    5'-AUG...CCACC        GACUC...UGA-3'
                           UGGUGCGC
                           ||||||||
                           ACCACGCG
sRNA 60      3'-AAA...GGGAC        GCUCA...GUA-5'
                        |        |
Position                56       47
```

Energy -9.87 kcal/mol

J1

```
Position                847                                                    896
                        |                                                      |
pcaB mRNA    5'-AUG...CAAGC  A    G           CC                G                CGGGC...UGA-3'
                           GC ACCC GUGG GUGCCG  GUGUUGA    UCGGU CUG CGACCCGCGUGC
                           || |||| |||| |:||||  |||:::|    ||||| ||| |:||||||||:|
                           CG UGGG CACC CGCGGC  CACGGUU    AGCCA GAC GUUGGGCGCAUG
sRNA 60      3'-AAA...ACACC       A    A      U        GCAG         A            UCGAC...GUA-5'
                        |                                                      |
Position                64                                                     12
```

Energy -13.44 kcal/mol

K1

```
Position                63      71
                        |       |
CMD2 mRNA    5'-AUG...ACUAC       GCUCG...UAA-3'
                           GUCAACC
                           |||||||
                           CAGUUGG
sRNA 60      3'-AAA...CCAGA       GCGCA...GUA-5'
                        |       |
Position                27      19
```

Energy -6.67 kcal/mol

L1

```
Position                727         739
                        |           |
pcaD mRNA    5'-GUG...GCACC  U        GAGAU...UGA-3'
                           UG CCAACGUC
                           || ||||||||
                           AC GGUUGCAG
sRNA 60      3'-AAA...GGCUC           AGCCA...GUA-5'
                        |           |
Position                44          33
```

Energy -6.67 kcal/mol

M1

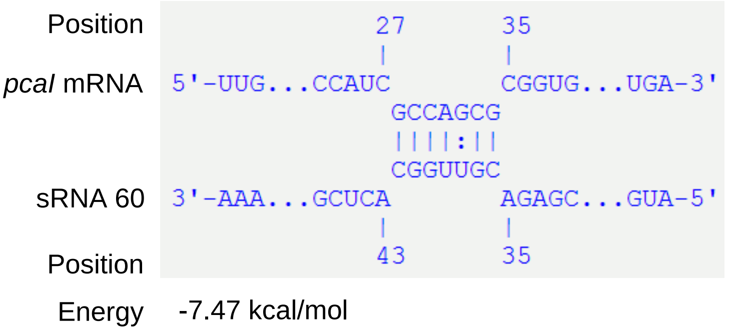

FIGURE S9 Interaction prediction of sRNA 8-*ech* mRNA (**A**), sRNA 8-*vdh* mRNA (**B**), sRNA 8-*vanA* mRNA (**C**), sRNA 8-*pobA* mRNA (**D**), sRNA 8-*pcaH* mRNA (**E**), sRNA 8-*pcaG* mRNA (**F**), sRNA 8-*pcaB* mRNA (**G**), sRNA 8-*pcaD* mRNA (**H**), sRNA 8-*ACAT* mRNA (**I**), sRNA 11-*vdh* mRNA (**J**), sRNA 11-*pobA* mRNA (**K**), sRNA 11-*pcaJ* mRNA (**L**), sRNA 11-*pcaF* mRNA (**M**), sRNA 14-*fcs* mRNA (**N**), sRNA 14-*ech* mRNA (**O**), sRNA 14-*vdh* mRNA (**P**), sRNA 14-*pobA* mRNA (**Q**), sRNA 14-*pcaH* mRNA (**R**), sRNA 14-*pcaG* mRNA (**S**), sRNA 14-*pcaB* mRNA (**T**), sRNA 14-*CMD1* mRNA (**U**), sRNA 14-*pcaD* mRNA (**V**), sRNA 14-*pcaJ* mRNA (**W**), sRNA 14-*pcaF* mRNA (**X**), and sRNA 14-*ACAT* mRNA (**Y**), sRNA 20-*ech* mRNA (**Z**), sRNA 20-*pobA* mRNA (**A1**), sRNA 20-*pcaB* mRNA (**B1**), sRNA 20-*pcaF* mRNA (**C1**), sRNA 20-*ACAT* mRNA (**D1**), sRNA 60-*fcs* mRNA (**E1**), sRNA 60-*vdh* mRNA (**F1**), sRNA 60-*vanB* mRNA (**G1**), sRNA 60-*pcaH* mRNA (**H1**), sRNA 60-*pcaG* mRNA (**I1**), sRNA 60-*pcaB* mRNA (**J1**), sRNA 60-*CMD2* mRNA (**K1**), sRNA 60-*pcaD* mRNA (**L1**), and sRNA 60-*pcaI* mRNA (**M1**)
